# Comparative Effectiveness of Multiple Exercise Interventions in the Treatment of Mental Health Disorders: A Systematic Review and Network Meta-Analysis

**DOI:** 10.1186/s40798-022-00529-5

**Published:** 2022-10-29

**Authors:** Qian Yu, Ka-Kit Wong, On-Kei Lei, Jinlei Nie, Qingde Shi, Liye Zou, Zhaowei Kong

**Affiliations:** 1 Faculty of Education, University of Macau, Macao, China; 2 Faculty of Health Sciences and Sports, Macao Polytechnic University, Macao, China; 3 Body-Brain-Mind Laboratory, The Shenzhen Humanities & Social Sciences Key Research Bases of the Center for Mental Health, School of Psychology, Shenzhen University, Shenzhen, 518060 China

**Keywords:** Depression, Anxiety disorder, Post-traumatic stress disorder, Schizophrenia, Exercise, Training

## Abstract

**Background:**

The efficacy of exercise interventions in the treatment of mental health disorders is well known, but research is lacking on the most efficient exercise type for specific mental health disorders.

**Objective:**

The present study aimed to compare and rank the effectiveness of various exercise types in the treatment of mental health disorders.

**Methods:**

The PubMed, Web of Science, PsycINFO, SPORTDiscus, CINAHL databases, and the Cochrane Central Register of Controlled Trials as well as Google Scholar were searched up to December 2021. We performed pairwise and network meta-analyses as well as meta-regression analyses for mental health disorders in general and each type of mental health disorder, with alterations in symptom severity as the primary outcome.

**Results:**

A total of 6456 participants from 117 randomized controlled trials were surveyed. The multimodal exercise (71%) had the highest probability of being the most efficient exercise for relieving depressive symptoms. While resistance exercise (60%) was more likely to be the most effective treatment for anxiety disorder, patients with post-traumatic stress disorder (PTSD) benefited more from mind–body exercise (52%). Furthermore, resistance exercise (31%) and multimodal exercise (37%) had more beneficial effects in the treatment of the positive and negative symptoms of schizophrenia, respectively. The length of intervention and exercise frequency independently moderated the effects of mind–body exercise on depressive (coefficient = 0.14, *p* = .03) and negative schizophrenia (coefficient = 0.96, *p* = .04) symptoms.

**Conclusion:**

Multimodal exercise ranked best for treating depressive and negative schizophrenic symptoms, while resistance exercise seemed to be more beneficial for those with anxiety-related and positive schizophrenic symptoms. Mind–body exercise was recommended as the most promising exercise type in the treatment of PTSD. However, the findings should be treated with caution due to potential risk of bias in at least one dimension of assessment and low-to-moderate certainty of evidence.

*Trial Registration* This systematic review was registered in the PROSPERO international prospective register of systematic reviews (CRD42022310237).

**Supplementary Information:**

The online version contains supplementary material available at 10.1186/s40798-022-00529-5.

## Key Points

- The multimodal exercise had the highest probability of being the most effective exercise for relieving depression and negative symptoms of schizophrenia.
- The resistance exercise had more benefits in the treatment of anxiety disorder and positive symptoms of schizophrenia.
- The mind–body exercise was most likely to be the most efficient exercise for relieving post-traumatic stress syndrome.

## Introduction

Mental health disorder (also termed mental disorder, mental illness, or psychiatric disorder) refers to a syndrome generated by clinical disturbances in cognition, emotion regulation, thinking, and behavior [1]. Prevalent mental health disorders include depression, anxiety disorder, post-traumatic stress disorder (PTSD), and schizophrenia [2, 3]. Approximately 29% of the population may suffer from at least one type of mental health disorder during their lifetime, and nearly half of the people with severe mental health disorders experience complications (e.g., cardiorespiratory diseases, stomatognathic diseases, and obesity-related cancers) [4], shortened life expectancy of 10–20 years, and excess mortality rates [5]. The substantial social and economic costs of mental health disorders pose significant burdens on individuals, families, and society [6]. During the coronavirus disease 2019 pandemic, lockdowns and socioeconomic stress not only led to less provision of medical care for psychiatric patients but also increased the morbidity of mental health disorders [7–9], which dealt an unprecedentedly severe blow to the global health care system [10–12]. Economic, practical, and effective treatment of mental health disorders is urgently needed.

Due to the uneven distribution of resources, side effects, and low patient compliance, currently pharmacological therapies lack stability and significant efficacy [13]. By contrast, evidence that non-pharmacological approaches (including exercise intervention) can improve mental health is extensive and growing [14]. According to the literature, exercise interventions benefit patients with mental health disorders through physiological, immune, neurobiological, and psycho-behavioral mechanisms [15, 16]. Exercise improves mental health via various pathways, for example, by increasing endocannabinoid levels [17], up-regulating mitochondrial numbers and related oxygenation capabilities [18], enhancing the mammalian target of rapamycin signaling [19], and improving the hypothalamic pituitary-adrenal axis function [20]. From an immune system perspective, exercise programs improve psychiatric symptoms principally by lowering inflammation, for example, by systemically increasing cytokines [21], decreasing visceral fat mass [22], down-regulating toll-like receptors [23], and increasing vagal tone [24]. Neurobiologically, exercise interventions promote neurogenesis, angiogenesis, synaptic plasticity, and cerebrovascular function, each of which contributes to enhanced connectivity within large-scale brain networks [16]. Psycho-behaviorally, exercise distracts patients with mental health disorders from negative moods and boosts their self-esteem through self-efficacy and mastery [25]. Notably, different types of exercise interventions generate mental health benefits through the above mechanisms disproportionately and may have different effects on the same mental health disorder [15, 16]. Moreover, mental health disorders with distinct pathologies and pathogenesis respond to the same type of exercise program in different ways [15, 16]. However, studies comparing the simultaneous mental health benefits of various exercises are lacking, and traditional pairwise meta-analyses cannot pool evidence from multiple interventions. Thus, the most efficient type of exercise for a particular mental health disorder has not been established, and this hinders clinical decision-making.

Network meta-analysis, a promising synthesis methodology in the field of sports science and health promotion, outperforms conventional pairwise meta-analysis by integrating and quantifying both direct and indirect evidence, estimating the relative effectiveness of multiple interventions simultaneously, and ranking treatments as part of the process of clinical decision-making [26]. Thus, we evaluated and compared the effectiveness of various exercise types in the improvement of primary mental health symptoms among populations diagnosed with mental health disorders (i.e., depression, anxiety disorder, PTSD, and schizophrenia) via network meta-analysis of relevant randomized controlled trials (RCTs). We also examined the moderating role of other exercise characteristics (frequency, intensity, session duration, and the length of intervention) in the association between exercise intervention and symptom severity.

## Methods

We registered our systematic review and network meta-analysis with PROSPERO (CRD42022310237) and used the Preferred Reporting Items for Systematic Reviews and Meta-Analyses-Network Meta-Analyses (PRISMA-NMA) checklist (Additional file 2: Appendix S1) [27].

### Searching Strategy

The PubMed, Web of Science, PsycINFO (via EBSCOhost), SPORTDiscus, and Cumulative Index to Nursing and Allied Health Literature (CINAHL) databases; the Cochrane Central Register of Controlled Trials (CENTRAL); and Google Scholar were searched online for the period up until December 2021. The search strategy was reviewed by experts from the fields of mental health and sports science (Additional file 3: Appendix S2). The reference lists of relevant systematic reviews published in the last three years were also cross-checked.

### Study Selection and Eligibility Criteria

Duplicated references were removed using the deduplication function of EndNote 20 software (Clarivate Analytics, Philadelphia, PA, USA) and manually. Two investigators (QY and KW) independently screened titles and abstracts to identify all relevant studies and then assessed the full texts according to the pre-set criteria. Disagreements were resolved through discussion or consultation with a third expert (ZK).

To evaluate and compare the effectiveness of various exercise types in the improvement of primary mental health symptoms among patients with mental health disorders (i.e., depression, anxiety disorder, PTSD, and schizophrenia), the inclusion criteria were as follows: (1) participants: full diagnosis of depression, anxiety disorder, PTSD, or schizophrenia by professionals according to accepted criteria (i.e., *Diagnostic and Statistical Manual of Mental Disorders* [*DSM*; 4th and 5th eds.] and *International Statistical Classification of Diseases and Relating Health Problems* [*ICD;* 9th and 10th eds.]); (2) interventions: any type of exercise; (3) comparator: usual care, health education, or other type of exercise not applied in the experimental group; (4) outcomes: mental health symptom severity assessed by clinical assessment scales (i.e., depressive, anxiety, post-traumatic stress, and overall, positive, and negative schizophrenia symptoms); and (5) study design: RCTs comparing an exercise intervention with either a non-exercise intervention or other forms of exercise intervention, with a total sample size of more than 20 participants to reduce the risk of publication bias (Inheriting most methodological practices of pairwise meta-analysis, the network meta-analysis has greater complexity due to multiple comparisons [28].) The effective sample size refers to the number of participants that could provide the same degree and evidence strength in both direct and indirect comparisons [28, 29]. Considering the direct comparison, the indirect comparison, the interactions between direct and indirect comparisons, the study type and the characteristics of research topic, we followed the practice of Owen et al. [28–30]. All studies had to have been published in English in peer-reviewed journals. If multiple studies were determined to have used data from the same cohort, the study with the longest follow-up was included. When the studies had the same follow-up period, the one with the largest sample size was included.

To ensure that the included studies were homogeneous for statistical comparisons, the exclusion criteria were as follows: (1) participants diagnosed with more than one of the mental health disorders referred to in the inclusion criteria; (2) participants diagnosed with other type of physical or mental diseases (i.e., cancer, diabetes, hypertension, infection, osteoporosis, obsessive–compulsive disorder, autism, and mild cognitive impairment); (3) participants who had adopted an intervention (e.g., cognitive-behavior therapy, music therapy) other than exercise and daily medication; and (4) the study examined the effects of acute exercise.

### Data Extraction

Two investigators (OL and JN) independently extracted the data from a self-designed statistical form. They enlisted the aid of a third investigator (ZK) when needed. The statistical form included study characteristics (author’s name and publication year); participant characteristics (disorder type, sample size and age); intervention (exercise type, frequency, intensity, session duration, length of intervention, and comparator information); and outcomes (the relevant pre-intervention and post-intervention statistics or post–pre-changed values for estimating effect size, and assessment tools). When the relevant statistics were not reported in the original article, the mean and standard deviation were estimated based on the sample size, median, range (the minimum and maximum values), and interquartile range [31, 32]. The ImageJ processing program [33] (V.1.50i, https://imagej.nih.gov/ij/) was used to calculate pixel value statistics of defined selections and to extract numerical data from figures in six of the studies [34–39]. We contacted the authors of five of the studies [40–44] via email and the ResearchGate network to request data twice a month; two responded positively [43, 44].

The *DSM*s and the *ICD*s were referred to for diagnosis, classification, and evaluation as part of the process of collecting data. If multiple assessment tools were applied in one study, the outcome from the most frequently used scale among all the studies was reported. The *Physical Activity Guidelines for Americans* [45] and previous systematic reviews [46, 47] for the classification of exercise interventions were referred to for information about exercise classification. We divided exercise interventions into the following broad categories [30, 46, 47] to compare their effects: (1) aerobic exercise (AE), which is used to improve cardiorespiratory fitness through walking, jogging, running, and cycling; (2) resistance exercise (RE), which is used to increase muscle strength and endurance through weight machines and resistance bands; (3) mind–body exercise (MBE), which is used to focus inwardly through, for instance, Tai Chi, yoga, Yijinjing, and dance [48]; (4) stretching (a static or dynamic exercise that increases muscle control, flexibility, and range of motion); (5) multimodal exercise (ME), wherein at least two types of exercise, such as AE and RE, are combined; and (6) other types of exercise (i.e., an exercise intervention that could not be placed into the above categories), for example, balance training or high-intensity interval exercise [HIIE].

### Risk of Bias Assessment and GRADE

The risk of bias (RoB) for all studies was independently evaluated by two investigators (QS and LZ) using the Cochrane Collaboration’s risk-of-bias tool (RoB version 2.0) [49]. Bias arising from randomization; deviations from intended interventions; missing outcome data; outcome measurement; and selected reports were assessed. The Grading of Recommendations Assessment, Development and Evaluation (GRADE) approach was used to assess the certainty of the evidence [50]. The GRADE certainty was rated down one (serious concern) or two grades (very serious concern) for reasons including risk of bias, inconsistency, indirectness, imprecision, and publication bias [50]. The effect estimate with sufficient, moderate, limited, or very little confidence was rated as high, moderate, low, and very low certainty, respectively [50]. Similarities between assessments of RoB and certainty of evidence between the two investigators were calculated, and the third investigator (ZK) was consulted in the event of disagreement.

### Data Synthesis and Analysis

Pairwise meta-analyses were conducted to compare the effect of one exercise type with another or the control condition if there was a minimum of two studies [51]. Effect size was estimated using standardized mean difference (SMD) and 95% confidence interval (95% CI) using post–pre-mean change and standard deviation (SD) difference by the random effects model. Effect size was considered small (SMD < 0.40); moderate (SMD = 0.40–0.70); or large (SMD > 0.70), in accordance with the Cochrane handbook [49]. Heterogeneity, as assessed by the I^2^ statistic, was classified as very low (*I*^2^ < 25%); low (*I*^2^ = 25–50%); moderate (*I*^2^ = 50–75%); or high (*I*^2^ ≥ 75%).

Bayesian network analysis was conducted using Aggregate Data Drug Information System software (ADDIS version 1.16.8, Drugis, Groningen, NL); the Markov chain Monte Carlo (MCMC) methods were used to estimate the models [52]. When the consistency assessed by node-splitting analysis was shown between direct and indirect comparisons (*p* > 0.05), the random effects and consistency model were implemented based on the following parameters: (1) 4 chains; (2) 20,000 tuning iterations; (3) 50,000 simulation iterations; (4) thinning interval of 10; (5) 10,000 inference samples; and (6) a variance scaling factor of 2.5 [53]. The rank probabilities were then calculated for the treatment effectiveness of each intervention type to provide the basis of alternative selection [52]. The Brooks–Gelman–Rubin diagnosis was used to estimate convergence, with the potential scale reduction factor close to 1 indicating approximate convergence [54]. When there was inconsistency between direct and indirect comparisons, the inconsistency model was used [53]. For the open-loop comparisons, the node-splitting analysis was not applicable as there was no direct evidence [52]. A 95% credible interval (CrI) was allowed for probability distribution. A network diagram was generated according to the network meta-analysis, where each node referred to one intervention type (i.e., AE, RE, MBE, stretching, ME, others, and the control) and the line referred to studies in which interventions were compared directly [55]. Node size was weighted by the number of participants receiving the specific intervention, while the line’s thickness was weighted by the number of RCTs [55]. In addition, the outcomes of network meta-analyses were retested by WinBUGS [56] (version 1.4, Medical Research Council, Imperial College of Science, Technology and Medicine, University of Cambridge, UK) and the relevant BUGS codes for network meta-analyses were provided (Additional file 4: Appendix S3).

We performed the analyses above both for the mental health disorders in general and each type of mental health disorder (i.e., subgroup analyses for depression, anxiety disorder, PTSD, and schizophrenia [overall, positive, and negative symptoms]), because the type of disorder may have influenced the intervention effects. To explore the causes of heterogeneity further, meta-regression analyses were conducted when there were more than 10 relevant studies, with participants’ age, exercise frequency, intensity, session duration, and length of interventions as covariates. Due to incomplete information and a limited number of studies, meta-regression analyses were conducted for aerobic and mind–body exercise for overall mental health disorders, depression, and negative schizophrenic symptoms, without consideration of exercise intensity.

## Results

### Study Selection and Characteristics

**Fig. 1:**
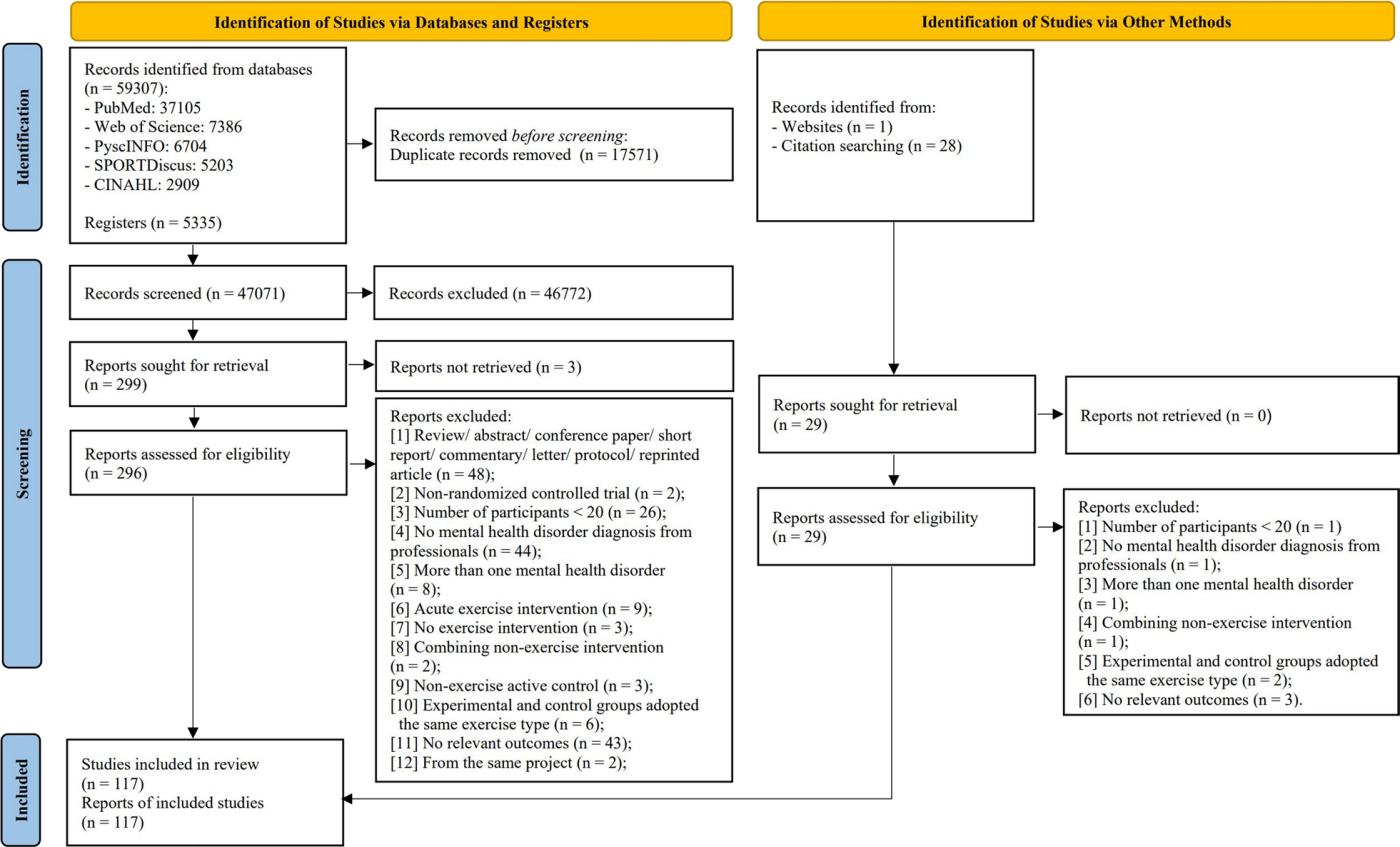
Flow diagram for systematic reviews which included searches of databases, registers, and other sources [27]

Possible studies for inclusion (*n* = 64,671) were identified in the databases (*n* = 59,307), the registers (*n* = 5335), and other sources (websites: *n* = 1; citation searching: *n* = 28) based on the requirements of the PRISMA 2020 flow diagram for new systematic review and meta-analysis. The search and selection process is shown in Fig. 1. A total of 6456 participants were finally included from 117 RCTs (depression: *n* = 70 [34–39, 57–120]; anxiety disorder: *n* = 5 [121–125]; PTSD: *n* = 11 [43, 126–135]; and schizophrenia: *n* = 31 [136–166] [overall symptoms: *n* = 16; positive symptoms: *n* = 26; negative symptoms: *n* = 28]) published between 1987 and 2021 (Additional files 5 and 6: Appendixes S4 and S5). The sample size of RCTs ranged from 20 to 244 participants. The age of participants ranged between 19 and 76 years old. Fifty-six studies (1510 participants) examined the effects of AE [34, 36–38, 57–59, 61, 64, 66, 67, 69–72, 74–78, 85, 86, 88–96, 98, 101, 102, 106, 107, 109, 111, 112, 114, 118, 121, 122, 124, 136–138, 141, 147, 150, 155–158, 160, 164], while 9 studies (161 participants) evaluated RE [57, 60, 63, 70, 122, 125, 134, 149, 166], 49 studies (1530 participants) evaluated MBE [35, 43, 62, 73, 79–84, 87, 93, 97, 99, 100, 103–105, 110, 115–117, 119, 120, 123, 126–131, 136, 138, 139, 141–144, 146, 148, 151–154, 159, 161–163, 165], 9 studies (212 participants) for stretching [37, 66, 68, 78, 92, 109, 111, 155, 157], 10 studies (276 participants) evaluated ME [39, 65, 108, 113, 132, 133, 135, 140, 145, 151], 3 studies (71 participants) evaluated other exercise types [38, 102, 164], and 98 studies (2696 participants) evaluated control conditions [34–36, 39, 43, 58–65, 67–69, 71–77, 79–91, 94–101, 103–108, 110, 112–121, 123–135, 137, 139, 140, 142–150, 152–154, 156, 158–163, 165, 166].

### Risk of Bias and GRADE

**Fig. 2:**
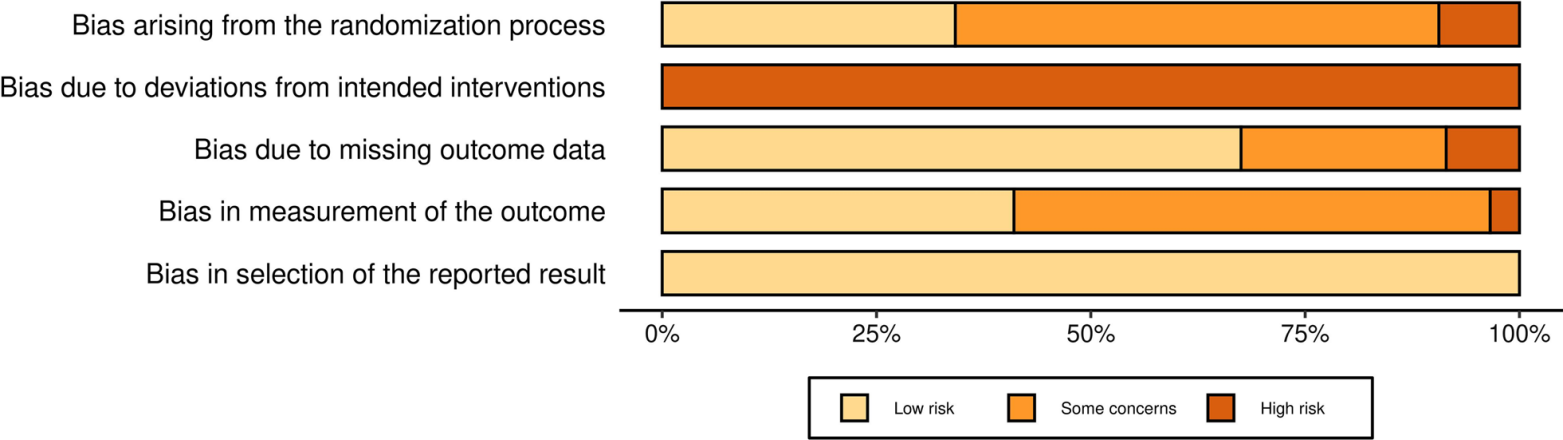
Risk of Bias Assessment. *Notes*: Domain 1: Bias from randomization process; Domain 2: Bias due to deviations from intended interventions; Domain 3: Bias due to missing outcome data; Domain 4: Bias in measurement of outcome; and Domain 5: Bias in selection of reported result

The outcome of the RoB assessment (Additional file 7: Appendix S6 and Fig. 2) was as follows: (1) bias from randomization (high risk: 9.40%, some concerns: 56.41%, low risk: 34.19%); (2) bias due to deviations from intended interventions (high risk: 100%); (3) bias due to missing outcome data (high risk: 8.55%, some concerns: 23.93%, low risk: 67.52%); (4) bias in measurement of outcome (high risk: 3.42%, some concerns: 55.56%, low risk: 41.03%); and (5) bias in selection of reported result (low risk: 100%). The certainty of evidence for outcomes ranged from very low-to-moderate levels (Additional file 8: Appendix S7). The similarities in RoB and GRADE assessments between the two investigators were 80.68% and 82.91%, respectively.

### Treatment for Mental Health Disorders in General

**Fig. 3:**
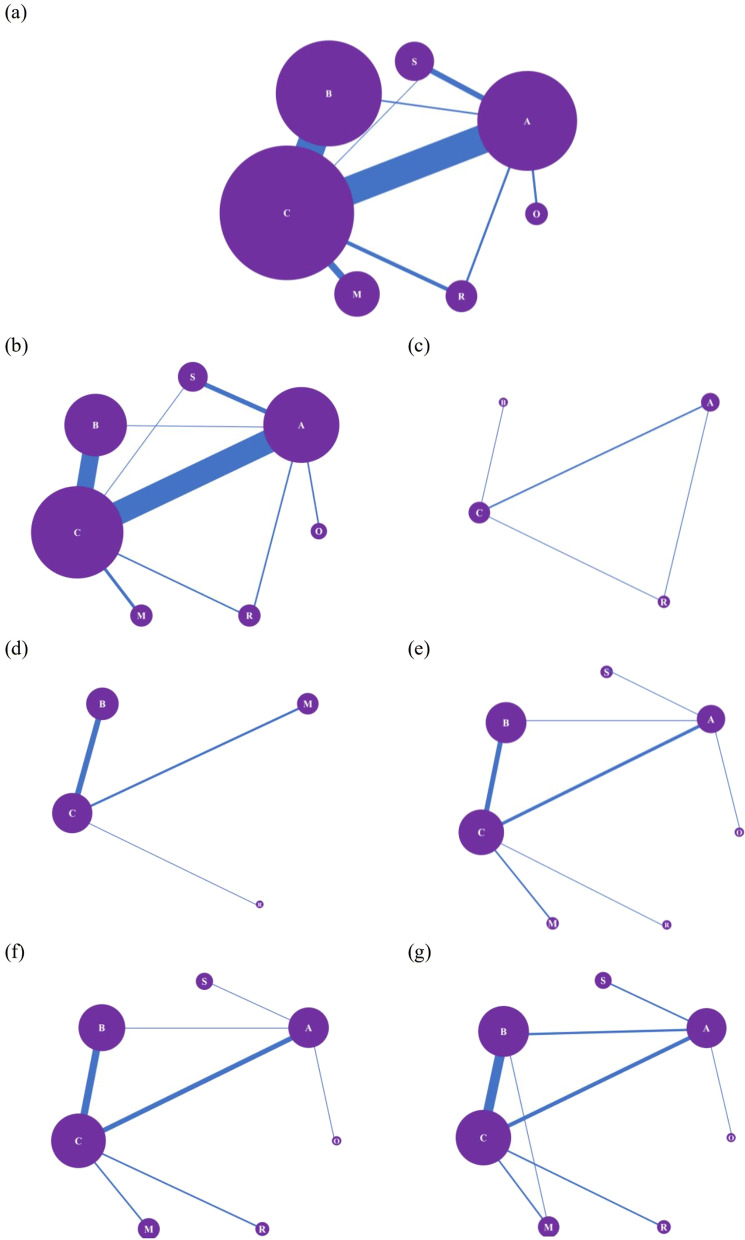
Network meta-analyses of eligible comparisons for **a** mental health disorders in general, **b** depression, **c** anxiety disorder, **d** post-traumatic stress disorder, **e** overall, **f** positive and **g** negative schizophrenic symptoms. The network diagram was generated according to network meta-analyses, where each node referred to intervention type and the line referred to studies in which interventions were compared directly. Node size was weighted by the number of participants receiving the specific intervention, while the line’s thickness was weighted by the number of RCTs. Abbreviations: A, aerobic exercise; R, resistance exercise; B, mind–body exercise; S, stretching; M, multimodal exercise; O, other types of exercise; and C, control

A total of 36 studies directly investigated the treatment effects on mental health symptoms of AE vs. control condition [34, 36, 58, 59, 61, 64, 67, 69, 71, 72, 74–77, 85, 86, 88–91, 94–96, 98, 101, 106, 107, 112, 114, 118, 121, 124, 137, 156, 158, 160], while 36 studies evaluated MBE vs. the control [35, 43, 62, 73, 79–84, 87, 97, 99, 100, 103–105, 110, 115–117, 119, 120, 123, 126–131, 142, 144, 152, 153, 161, 162], 5 studies evaluated RE vs. the control [60, 63, 125, 134, 149], 9 studies evaluated ME vs. the control [39, 65, 108, 113, 132, 133, 135, 140, 145], 1 study evaluated stretching vs. the control [68], 3 studies evaluated AE vs. RE [57, 70, 122], 2 studies evaluated AE vs. MBE [93, 141], 7 studies evaluated AE vs. stretching [37, 66, 78, 92, 109, 111, 157], and 3 studies evaluated AE vs. others [38, 102, 164] (Fig. 3a).

Pairwise meta-analyses revealed significant differences in the six comparisons of AE vs. the control (SMD, 0.91; 95% CI 0.48–1.34), MBE versus the control (SMD, 0.81; 95% CI 0.56, 1.05), RE versus the control (SMD, 1.72; 95% CI 0.49, 2.95), ME versus the control (SMD, 1.62; 95% CI 0.89, 2.36), AE versus RE (SMD, − 0.49; 95% CI − 0.82, − 0.16), and AE versus stretching (SMD, 0.55; 95% CI 0.11, 0.99) (Additional file 9: Appendix S8.1.1). The remaining two direct comparisons—AE versus MBE (SMD, − 0.14; 95% CI − 1.23, 0.94) and AE versus others (HIIE) (SMD, 0.55; 95% CI − 0.53, 1.63)—were not significantly different (Additional file 9: Appendix S8.1.1).

**Fig. 4:**
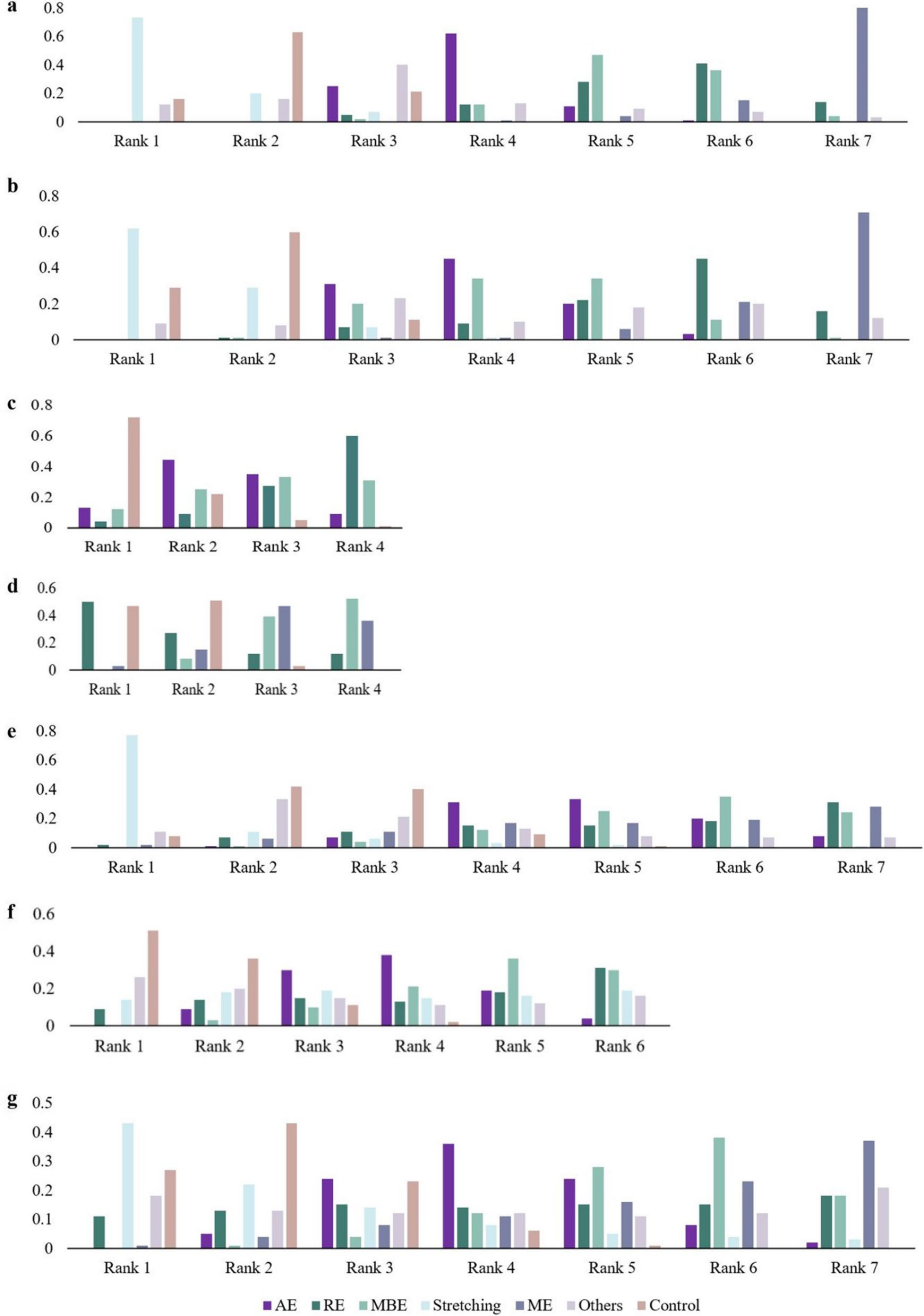
Rank probabilities for treatment of **a** mental health disorders in general, **b** depression, **c** anxiety disorder, **d** post-traumatic stress disorder, **e** overall schizophrenic symptom, **f** positive schizophrenic symptom, and **g** negative schizophrenic symptom. *Notes*: Rank 1 is the worst, and rank last is the best. AE, aerobic exercise; RE, resistant exercise; MBE, mind–body exercise; and ME, multimodal exercise

As node-splitting analysis revealed no significant inconsistency (all the *p* values were > 0.05), a network meta-analysis based on the consistency model was conducted (Additional file 9: Appendix S8.1.2). The pooled results from the network meta-analysis suggested that ME was superior to AE (SMD, − 4.14; 95% CrI − 7.59, − 0.77) and stretching was inferior to all the other exercise interventions (Additional file 9: Appendix S8.1.2). According to the rank probabilities (Fig. 4a and Additional file 9: Appendix S8.1.2), ME was most likely to rank best, followed by RE, MBE, AE, and stretching. Meta-regressions were conducted separately for AE and MBE, with participants’ age, exercise frequency, session duration, and length of the intervention as covariates (Additional file 9: Appendix S8.1.3). A meta-regression analysis of AE indicated that the length of intervention could be one of the moderators affecting treatment effects on mental health symptoms in general, whereas no covariate significantly influenced the efficacy of MBE (coefficient, 0.07; *p* = 0.04) (Additional file 9: Appendix S8.1.3).

### Treatment for Depression

A total of 30 studies directly examined the efficacy on depressive symptoms of AE vs. control condition [34, 36, 58, 59, 61, 64, 67, 69, 71, 72, 74–77, 85, 86, 88–91, 94–96, 98, 101, 106, 107, 112, 114, 118], while 2 studies evaluated RE versus the control [60, 63], 22 studies evaluated MBE versus the control [35, 62, 73, 79–84, 87, 97, 99, 100, 103–105, 110, 115–117, 119, 120], 4 studies evaluated ME versus the control [39, 65, 108, 113], 1 study evaluated stretching versus the control [68], 2 studies evaluated AE versus RE [57, 70], 1 study evaluated AE versus MBE [93], 6 studies evaluated AE versus stretching [37, 66, 78, 92, 109, 111], and 2 studies evaluated AE versus others [38, 102] (Fig. 3b). Pairwise meta-analyses revealed significant differences in the following five comparisons: AE versus the control (SMD, 0.98; 95% CI 0.46–1.50), MBE vs. the control (SMD, 0.78; 95% CI 0.46, 1.11), ME vs. the control (SMD, 2.66; 95% CI 0.96, 4.37), AE versus. RE (SMD, − 0.56; 95% CI − 0.92, − 0.20), and AE versus stretching (SMD, 0.49; 95% CI 0.00, 0.99). There was no significant difference between AE versus other types of exercise (SMD, − 0.06; 95% CI − 0.43, 0.31) (Additional file 9: Appendix S8.2.1). A network meta-analysis based on the consistency model indicated that ME led to more improvement in depressive symptoms than AE (Additional file 9: Appendix S8.2.2). The rank probabilities of all exercise interventions in terms of depressive symptom improvement were generated as follows (from the best to the worst): ME, RE, MBE, AE, and stretching (Fig. 4b and Additional file 9: Appendix S8.2.2). The meta-regression for MBE suggested that the length of intervention positively moderated the effectiveness of MBE on depressive symptoms (coefficient, 0.14; *p* = 0.03) (Additional file 9: Appendix S8.2.3).

### Treatment for Anxiety Disorder

Comparisons between the effects of different protocols on anxiety disorder [121–125] are shown in Fig. 3c. Both the pairwise analysis and network analysis based on the consistency model revealed no significant differences among AE, RE, MBE, and the control (Additional file 9: Appendix S8.3.1 and 8.3.2). The ranking of exercise interventions suggested that RE had the highest probability (60%) of being the most effective form of exercise in treating anxiety-related symptoms (Fig. 4c).

### Treatment for Post‐traumatic Stress Disorder

For PTSD, a total of 11 studies with 576 participants were included [43, 126–135] **(**Fig. 3d**)**. In the pairwise meta-analyses, MBE (SMD, 0.80; 95% CI 0.47, 1.13) and ME (SMD, 1.25; 95% CI 0.78, 1.72) were both directly compared to the control and had significant positive effects on post-traumatic stress symptoms (Additional file 9: Appendix S8.4.1). As the closed loop was not constructed in the network meta-analysis for PTSD, the node-splitting analysis was not applicable. Network meta-analysis based on the consistency model did not reveal any significant differences between RE, MBE, and ME (Additional file 9: Appendix S8.4.2). The ranking probability plot indicated that the MBE was most likely to rank best, followed by ME and RE (Fig. 4d).

### Treatment for Schizophrenia

Among the 31 studies that targeted schizophrenia [136–166], 16 reported results associated with overall schizophrenic symptoms [137, 140–142, 144, 145, 149, 152, 153, 156–158, 160–162, 164], 26 associated with positive symptoms [136–138, 140–152, 155–158, 160, 162–166], and 28 associated with negative symptoms [136, 138–152, 154–160, 162–166]. The evidence structure of comparisons in overall, positive, and negative schizophrenic symptoms is provided in Fig. 3e–g. The pairwise meta-analyses for overall, positive, and negative schizophrenic symptoms are shown in Additional file 9: Appendix S8.5.1, S8.6.1, and S8.7.1. The results of the node-splitting analyses indicate inconsistencies between direct and indirect comparisons in the model for positive symptoms, but not in the models for overall and negative symptoms. After adjusting the model for positive symptoms by removing arms connected with ME, all network meta-analyses were conducted using the consistency model (Additional file 9: Appendix S8.5.2, S8.6.2, and S8.7.2). The ranking probability plots suggest that RE was most likely to rank best in the treatment of overall and positive symptoms, whereas ME had the highest probability to rank best in the case of negative symptoms (Fig. 4e–g). MBE ranked second in the treatment of overall, positive, and negative schizophrenia symptoms. For negative symptoms, meta-regression revealed that the effectiveness of MBE was moderated by exercise frequency (coefficient, 0.96; *p* = 0.04) (Additional file 9: Appendix S8.7.3).

## Discussion

Network meta-analysis was conducted to evaluate and compare the effectiveness of various exercise interventions on mental health disorders (depression, anxiety disorder, PTSD, and schizophrenia). Pairwise and network analyses were conducted separately for overall, positive, and negative schizophrenia symptoms. The present review included 6456 participants from 117 RCTs published in the last 35 years. It is the first to examine the efficacy of distinct exercise types (AE, RE, MBE, ME, stretching, and others) in the treatment of four common mental health disorders. The findings demonstrate that the response to exercise interventions differed according to the condition: ME ranked best in the treatment of overall mental health, depressive, and negative schizophrenia symptoms; RE, anxiety-related and positive schizophrenia symptoms; and MBE, PTSD symptoms. The length of intervention positively moderated the effects of AE on overall mental health disorders and the effects of MBE on depression, while exercise frequency moderated the effects of MBE on negative schizophrenia symptoms.

Aerobic exercise, MBE, and ME all showed significant effects on depressive symptoms when compared with control conditions, with moderate-to-large effect sizes—all SMDs > 0.78. This suggested that an array of distinct exercise modalities may have clinically meaningful benefits in the treatment of depression and that clinicians working with depressive patients should consider exercise as complement to daily medication. According to the present network meta-analysis, ME had the highest probability of being the optimal exercise for improving depressive symptoms of all the interventions (i.e., AE, RE, MBE, ME, stretching, and other types of exercise [such as HIIE]), which went further than a previous network meta-analysis that examined the effects of AE, RE, and MBE on depression [167]. Hence, it is reasonable to infer that a combination of various exercise types was more likely to contribute to greater improvements in depression. Additionally, the outcome of the meta-regression indicated that the length of intervention (4–12 weeks) rather than exercise frequency and session duration positively moderated the effectiveness of MBE. This was partly consistent with previous meta-analyses in which the length of exercise was the only characteristic related to the effect size for depression treatment [168, 169], and where 9–12 weeks were associated with the largest reduction. Future researchers might retest these findings when studies with longer intervention periods are available; those involving extended MBE programs for depression (i.e., > 12 weeks) are currently lacking.

For anxiety disorder, direct and indirect comparisons between AE, RE, MBE, and controls did not show significant differences in treatment effects, but a reduction in anxiety-related symptoms could be observed in every case. Previous meta-analyses also demonstrated little to no benefits in exercise interventions for anxiety disorder [170, 171]. Based on the ranking probability plot, RE had the highest probability to be the most effective form of exercise, partly because it increases IGF-1 levels [172] and reduces inflammation [173] during training. Meanwhile, psychosocial factors such as improved self-efficacy [174], goal achievement [175], and mastery [176] also contribute to a relief in symptoms. It should be noted, however, that the above trials involved limited sample sizes, so the findings should be treated with caution.

Both pairwise and network meta-analyses suggested the MBE might ameliorate the symptoms of PTSD. Correspondingly, MBE was recommended as the most promising form of exercise based on the current ranking probability plot. In line with our findings, existing pairwise meta-analyses have demonstrated MBE to be an efficient adjuvant therapy in the improvement of PTSD-related symptoms, with effect sizes ranging from − 0.41 to − 0.39 [177, 178]. Subgroup analyses in a previous meta-analysis indicated that 60–150 min of MBE 1–3 times per week over 8–16 weeks resulted in benefits to those experiencing PTSD symptoms [178]. The possible psychophysiological mechanisms underlying the efficacy of MBE may include the down-regulation of inflammatory markers [179]; corticotropin-releasing hormone concentration [180]; the up-regulation of cortisol level [181]; and desensitization to inner arousal [182]. In addition, a closed loop was not generated in the present network meta-analysis for PTSD because distinct exercise types were not compared directly. Future researchers might evaluate and compare directly the efficacy of various exercise programs in the treatment of PTSD.

Schizophrenia is a psychiatric disorder characterized by positive symptoms of delusion, hallucination, and disorganized speech and by negative symptoms of blunted affect, alogia, anhedonia, asociality, avolition, among others [183]. For positive schizophrenic symptoms, RE was considered the preferred exercise intervention based on the present ranking probabilities. One explanation for this effect was that RE had more competitive advantages in attention control facilitation than other exercise interventions [184] because schizophrenia patients with positive symptoms have significant disturbances in sensory-motor gating, shifting, spatial focus, and cue detection [185]. According to the findings, ME outperformed the other exercise types for negative symptoms. Given the fact that schizophrenia patients tended to be more sedentary than healthy controls [186], this conclusion may encourage this population to engage in exercise by offering them multiple daily exercise program choices. It was also observed that MBE ranked the second most efficient exercise intervention for treating positive and negative symptoms, with exercise frequency moderating the effects on negative symptoms. Previous meta-analyses [187, 188] have shown that MBE, which is based on the exercise trinity (i.e., body, mind, and breathing), is a well-established complementary therapy for people with schizophrenia. In addition, the meditation nature of MBE alleviates psychotic symptoms by improving autonomic nerve function and reconstructing large-scale brain networks [189].

### Future Recommendations for Reporting of Outcomes

Our Bayesian network meta-analysis has implications for further clinical practice and research in the field of mental health disorders. First, and in general, exercise interventions—regardless of type, frequency, or duration—help relieve psychiatric symptoms and should therefore be recommended as a routine complementary therapy for patients suffering from mental health disorders. Second, responses to intervention vary according to exercise type. While ME seems to be more suitable for patients with depression than other exercise interventions, those with anxiety disorder and PTSD might benefit more from RE and MBE, respectively. For schizophrenia, the selection of exercise program should be determined according to the specific symptoms: RE appears to be more efficacious in relieving positive symptoms, while ME is preferred for negative symptoms. Third, given the general effectiveness of MBE in curing mental health disorders, the combination of psychotherapeutic techniques (i.e., meditation and breathing) and exercise intervention should be further investigated. Fourth, the length of intervention positively moderates the efficacy of certain exercise interventions (i.e., AE and MBE). Thus, clinicians who prescribe exercise should take the interests of different populations and the practicability of regimens into consideration to encourage consistency. Fifth, multidimensional factors relating to exercise adherence should be explored further. Finally, the subsyndromal mental health symptoms (e.g., subsyndromal depression, subsyndromal generalized anxiety disorder, subsyndromal PTSD), failing to meet the full diagnostic criteria of mental health disorders, are broadly regarded as risk factors for developing clinically significant mental health disorders [190–192]. However, the population with subsyndromal mental health symptoms is underdiagnosed and undertreated globally, which results in public health hazards [190–192]. Further studies the mental health field are awaited to establish the recognized and unified diagnosis and evaluation criteria of subsyndromal mental health symptoms and further explore the potential therapeutic effects of exercise interventions.

### Limitations

The present study has several limitations. First, some previous researchers have measured and described exercise intensity using multiple approaches, while others did not report exercise intensity numerically. Thus, exercise intensity has not been used as a moderator in the conduct of meta-regression analyses. As a result, the present study could not fully apply the frequency, intensity, type, and time (FITT) principle [193] and consider every exercise characteristic, which may have influenced the transitivity in the indirect comparisons. The standard for assessing the intensity of various exercise types awaits to be proposed. Second, for ethical and scientific reasons, the studies that were included in the present analysis allowed patients with psychiatric symptoms to take daily medications. Perhaps unsurprisingly, their regimens varied to a considerable degree. Hence, interactions between symptom, exercise, and medication were not explored in the present study. Third, a large proportion of the studies were evaluated with a high RoB in at least one dimension and low-to-moderate certainty of evidence. In light of these reservations, the outcomes of the present network meta-analysis should be interpreted with caution. Fourth, considering the publication bias and the effective sample size in multiple direct and indirect comparisons of network meta-analysis, the studies with a total sample size of less than 20 participants were not included in the current research [30]. The issue of effective sample size and power of network meta-analysis may be a hindrance to resolving uncertainty by synthetizing all available evidence and exploring causes of between-study heterogeneity [194]. Fifth, the protocol of this current systematic review and network meta-analysis was not published a prior, which may have resulted in publication bias and low reproducibility.

## Conclusion

The results indicate the significance of exercise interventions as a complementary therapy in patients with mental health disorders. The selection of exercise program should be determined by disorder type. Multimodal exercise has the highest probability of being the most efficacious exercise for relieving depressive symptoms; RE for anxiety disorder; MBE for PTSD; and RE and ME for the positive and negative symptoms of schizophrenia, respectively. Finally, it was discovered that the length of intervention and exercise frequency positively moderated the effects of MBE on depressive and negative schizophrenia symptoms, respectively. The current findings should be treated with caution due to potential risk of bias in at least one dimension of assessment and low-to-moderate certainty of evidence.

## Supplementary Information

**Additional file 1:** Appendix Outline.

**Additional file 2: Appendix 1.** PRISMA Network Meta-Analysis Checklist.

**Additional file 3: Appendix 2.** Searching Strategy.

**Additional file 4: Appendix 3.** WinBUGS Code for Network Meta-Analyses.

**Additional file 5: Appendix 4.** Included Studies List.

**Additional file 6: Appendix 5.** Basic Information of Included Studies.

**Additional file 7: Appendix 6.** Risk of Bias Assessment.

**Additional file 8: Appendix 7.** Grading of Recommendations, Assessment, Development and Evaluations (GRADE).

**Additional file 9: Appendix 8.** Outcomes of Pairwise Meta-Analysis, Network Meta-Analysis and Meta Regression.

## Data Availability

All data and material reported in this systematic review are from peer-reviewed publications. The WinBUGS code used are available in Additional file 4: Appendix S3.

## Abbreviations

ADDIS: Aggregate Data Drug Information System Software
AE: Aerobic exercise
CI: Confidence interval
CrI: Credible interval
DSM: Diagnostic and Statistical Manual of Mental Disorders
HIIE: High-intensity interval exercise
FITT: Frequency, intensity, type, and time
GRADE: Grading of Recommendations Assessment, Development and Evaluation
ICD: International Statistical Classification of Diseases and Relating Health Problems
MBE: Mind–body exercise
MCMC: Markov chain Monte Carlo
ME: Multimodal exercise
PRISMA-NMA: Preferred Reporting Items for Systematic Reviews and Meta-Analyses-Network Meta-Analyses
PTSD: Post-traumatic stress disorder
ROB: Risk of bias
RCT: Randomized controlled trials
RE: Resistance exercise
SD: Standard deviation
SMD: Standardized mean difference
