# Supplementary material for: Comparative Effectiveness of Multiple Exercise Interventions in the Treatment of Mental Health Disorders: A Systematic Review and Network Meta-Analysis
Source: Sports Med Open. 2022 Oct 29;8:135. doi: 10.1186/s40798-022-00529-5 (PMC9617247; doi:10.1186/s40798-022-00529-5)
Supplement: Supplementary file 1 — Additional file 1: Appendix Outline. [file 40798_2022_529_MOESM1_ESM.docx]

**Appendix Outline**

1. **Appendix 1. PRISMA Network Meta-Analysis Checklist**

**2. Appendix 2. Searching Strategy**

**3. Appendix 3. WinBUGS Code for Network Meta-Analyses**

*3.1 WinBUGS Code for Network Meta-Analysis of Mental Health Disorders in General*

*3.2 WinBUGS Code for Network Meta-Analysis of Depression*

*3.3 WinBUGS Code for Network Meta-Analysis of Anxiety Disorder*

*3.4 WinBUGS Code for Network Meta-Analysis of Post-Traumatic Stress Disorder*

*3.5 WinBUGS Code for Network Meta-Analysis of Overall Schizophrenic Symptom*

*3.6 WinBUGS Code for Network Meta-Analysis of Positive Schizophrenic Symptom*

*3.7 WinBUGS Code for Network Meta-Analysis of Negative Schizophrenic Symptom*

**4. Appendix 4. Included Studies List**

**5. Appendix 5. Basic Information of Included Studies**

**6. Appendix 6. Risk of Bias Assessment**

**7. Appendix 7. Grading of Recommendations, Assessment, Development and Evaluations (GRADE)**

**8. Appendix 8. Outcomes of Pairwise Meta-Analysis, Network Meta-Analysis and Meta Regression**

*8.1 Pairwise Meta-Analysis, Network Meta-Analysis and Meta Regression for Mental Health Disorders in General*

8.1.1 Pairwise Meta-Analysis for Mental Health Disorders in General

8.1.2 Network Meta-Analysis for Mental Health Disorders in General

8.1.3 Meta Regression for Mental Health Disorders in General

*8.2 Pairwise Meta-Analysis, Network Meta-Analysis and Meta Regression for Depression*

8.2.1 Pairwise Meta-Analysis for Depression

8.2.2 Network Meta-Analysis for Depression

8.2.3 Meta Regression for Depression

*8.3 Pairwise Meta-Analysis and Network Meta-Analysis for Anxiety Disorder*

8.3.1 Pairwise Meta-Analysis for Anxiety Disorder

8.3.2 Network Meta-Analysis for Anxiety Disorder

*8.4 Pairwise Meta-Analysis and Network Meta-Analysis for Post‐Traumatic Stress Disorder*

8.4.1 Pairwise Meta-Analysis for Post‐Traumatic Stress Disorder

8.4.2 Network Meta-Analysis for Post‐Traumatic Stress Disorder

*8.5 Pairwise Meta-Analysis and Network Meta-Analysis for Overall Symptom of Schizophrenia*

8.5.1 Pairwise Meta-Analysis for Overall Symptom of Schizophrenia

8.5.2 Network Meta-Analysis for Overall Symptom of Schizophrenia

*8.6 Pairwise Meta-Analysis and Network Meta-Analysis for Positive Symptom of Schizophrenia*

8.6.1 Pairwise Meta-Analysis for Positive Symptom of Schizophrenia

8.6.2 Network Meta-Analysis for Positive Symptom of Schizophrenia (Adjusted)

*8.7 Pairwise Meta-Analysis, Network Meta-Analysis and Meta-Regression for Negative Symptom of Schizophrenia*

8.7.1 Pairwise Meta-Analysis for Negative Symptom of Schizophrenia

8.7.2 Network Meta-Analysis for Negative Symptom of Schizophrenia

8.7.3 Meta Regression for Negative Symptom of Schizophrenia
