# Supplementary material for: Comparative Effectiveness of Multiple Exercise Interventions in the Treatment of Mental Health Disorders: A Systematic Review and Network Meta-Analysis
Source: Sports Med Open. 2022 Oct 29;8:135. doi: 10.1186/s40798-022-00529-5 (PMC9617247; doi:10.1186/s40798-022-00529-5)
Supplement: Supplementary file 3 — Additional file 3: Appendix 2. Searching Strategy. [file 40798_2022_529_MOESM3_ESM.docx]

**Appendix 2.** Searching Strategy for PubMed Database

| #1 | (exercise [mh]) OR (exercise* [tiab]) OR (exercising [tiab]) OR (exercise therapy [mh]) OR (exercise movement techniques [mh]) OR (physical exercise [tiab]) |
| --- | --- |
| #2 | (physical fitness [mh]) OR (fitness [tiab]) |
| #3 | physical activity [tiab] |
| #4 | physical training [tiab] |
| #5 | training [tiab] |
| #6 | sport* [tiab] |
| #7 | exertion* [tiab] |
| #8 | movement* [tiab] |
| #9 | aerobic* [tiab] |
| #10 | anaerobic* [tiab] |
| #11 | resistance [tiab] |
| #12 | endurance [tiab] |
| #13 | isokinetic [tiab] |
| #14 | isometric [tiab] |
| #15 | isotonic [tiab] |
| #16 | dynamic [tiab] |
| #17 | (weight lifting [tiab]) OR (weight-lifting [tiab]) OR (weight training [tiab]) OR (weight bearing [tiab]) OR (weight-bearing [tiab]) |
| #18 | strength* [tiab] |
| #19 | balance* [tiab] |
| #20 | run* [tiab] |
| #21 | jog* [tiab] |
| #22 | treadmill* [tiab] |
| #23 | walk* [tiab] |
| #24 | climbing [tiab] |
| #25 | stair [tiab] |
| #26 | swim* [tiab] |
| #27 | (bicycl* [tiab]) OR (cycle* [tiab]) OR (cycling [tiab]) OR (circuit training [tiab]) |
| #28 | (row [tiab]) OR (rows [tiab]) OR (rowing [tiab]) |
| #29 | (skate* [tiab]) OR (skating [tiab]) |
| #30 | danc* [tiab] |
| #31 | (mind-body exercise [tiab]) OR (mind body exercise [tiab]) OR (meditative movement [tiab])  (Tai Ji [mh]) OR (Tai Ji [tiab]) OR (Taiji [tiab]) OR (taiji [tiab]) OR (Tai-Ji [tiab]) OR (Taijiquan [tiab]) OR (Tai Chi [tiab]) OR (taichi [tiab]) OR (tai chi [tiab]) OR (T’ai Chi [tiab]) |
| #32 | (Qigong [mh]) OR (Qigong [tiab]) OR (Qi-gong [tiab]) OR (qigong [tiab]) OR (qi gong [tiab]) OR (chi gong [tiab]) OR (ch’i kung [tiab]) |
| #33 | (Baduanjin [tiab]) OR (baduanjin [tiab]) |
| #34 | (Wuqinxi [tiab]) OR (wuqinxi [tiab]) |
| #35 | (Yijinjing [tiab]) OR (yijinjing [tiab]) |
| #36 | (Yoga [mh]) OR (Yoga [tiab]) OR (yoga [tiab]) OR (pliability [tiab]) OR (pilate [tiab]) OR (pilates [tiab]) |
| **#37** | **#1 OR #2 OR #3 OR #4 OR #5 OR #6 OR #7 OR #8 OR #9 OR #10 OR #11 OR #12 OR #13 OR #14 OR #15 OR #16 OR #17 OR #18 OR #19 OR #20 OR #21 OR #22 OR #23 OR #24 OR #25 OR #26 OR #27 OR #28 OR #29 OR #30 OR #31 OR #32 OR #33 OR #34 OR #35 OR #36** |
| #38 | (mental health [mh]) OR (mental health [tiab]) OR (mental illness [tiab]) OR (mental disorder* [tiab]) OR (mental state [tiab]) OR (mentally ill [tiab]) OR (mental well-being [tiab]) |
| #39 | (depress* [tiab]) OR (depression [mh]) |
| #40 | (anxiety [tiab]) OR (anxious [tiab]) OR (panic [tiab]) OR (phobic [tiab]) OR (phobia* [tiab]) |
| #41 | (post-traumatic stress [tiab]) OR (post traumatic stress [tiab]) OR (trauma and stressor related disorders [mh]) OR (PTSD [tiab]) |
| #42 | (mood [tiab]) OR (bipolar [tiab]) |
| #43 | (psychosis [tiab]) OR (psychotic [tiab]) OR (psychos* [tiab]) OR (psychot* [tiab]) OR (psychiat* [tiab]) OR (schizo* [tiab]) OR (schizophre* [tiab]) OR (schizophrenia [tiab]) |
| **#44** | **#38 OR #39 OR #40 OR #41 OR #42 OR #43** |
| #45 | randomized controlled trial [pt] |
| #46 | controlled clinical trial [pt] |
| #47 | controlled trial [tiab] |
| #48 | controlled study [tiab] |
| #49 | controlled clinical study [tiab] |
| #50 | RCT [tiab] |
| #51 | randomiz* [tiab] |
| #52 | randomis* [tiab] |
| #53 | placebo [tiab] |
| #54 | drug therapy [tiab] |
| #55 | randomly [tiab] |
| #56 | trial [tiab] |
| #57 | groups [tiab] |
| **#58** | **#45 OR #46 OR #47 OR #48 OR #49 OR #50 OR #51 OR #52 OR #53 OR #54 OR #55 OR #56 OR #57** |
| **#59** | **#37 AND #44 AND #58** |
| #60 | limit #59 to “humans” and “English” |

**PubMed: 37105**

(Notes. [pt]: Publication Type; [tiab]: Title or Abstract; [mh]: MeSH Major Topic)
