# Supplementary material for: Comparative Effectiveness of Multiple Exercise Interventions in the Treatment of Mental Health Disorders: A Systematic Review and Network Meta-Analysis
Source: Sports Med Open. 2022 Oct 29;8:135. doi: 10.1186/s40798-022-00529-5 (PMC9617247; doi:10.1186/s40798-022-00529-5)
Supplement: Supplementary file 4 — Additional file 4: Appendix 3. WinBUGS Code for Network Meta-Analyses. [file 40798_2022_529_MOESM4_ESM.docx]

**Appendix 3. WinBUGS Code for Network Meta-Analyses**

**3.1 WinBUGS Code for Network Meta-Analysis of Mental Health Disorders in General**

| model {  for (i in 1:ns) {  # Likelihood for each arm  for (k in 1:na[i]) {  m[i, k] ~ dnorm(theta[i, k], prec[i, k])  theta[i, k] <- mu[i] + delta[i, k]  prec[i, k] <- pow(e[i, k], -2)  }  # Study-level relative effects  # The arms are given in the order (arm_1, arm_2, ..., arm_{n_a-1}, arm_{n_a}).  # The relative effects are parameterized as d[arm_1, arm_k].  w[i, 1] <- 0  delta[i, 1] <- 0  for (k in 2:na[i]) { # parameterize multi-arm trials using a trick to avoid dmnorm  delta[i, k] ~ dnorm(md[i, k], taud[i, k])  md[i, k] <- d[t[i, 1], t[i, k]] + sw[i, k]  taud[i, k] <- tau.d * 2 * (k - 1) / k  w[i, k] <- delta[i, k] - d[t[i, 1], t[i, k]]  sw[i, k] <- sum(w[i, 1:k-1]) / (k - 1)  }  }  # Relative effect matrix  d[1,1] <- 0  d[1,2] <- d.AE.Control  d[1,3] <- d.AE.MBE  d[1,4] <- d.AE.Control + d.Control.ME  d[1,5] <- d.AE.Others  d[1,6] <- d.AE.RE  d[1,7] <- d.AE.Stretching  d[2,1] <- -d.AE.Control  d[2,2] <- 0  d[2,3] <- -d.AE.Control + d.AE.MBE  d[2,4] <- d.Control.ME  d[2,5] <- -d.AE.Control + d.AE.Others  d[2,6] <- -d.AE.Control + d.AE.RE  d[2,7] <- -d.AE.Control + d.AE.Stretching  d[3,1] <- -d.AE.MBE  d[3,2] <- d.AE.Control + -d.AE.MBE  d[3,3] <- 0  d[3,4] <- d.AE.Control + -d.AE.MBE + d.Control.ME  d[3,5] <- -d.AE.MBE + d.AE.Others  d[3,6] <- -d.AE.MBE + d.AE.RE  d[3,7] <- -d.AE.MBE + d.AE.Stretching  d[4,1] <- -d.AE.Control + -d.Control.ME  d[4,2] <- -d.Control.ME  d[4,3] <- -d.AE.Control + d.AE.MBE + -d.Control.ME  d[4,4] <- 0  d[4,5] <- -d.AE.Control + d.AE.Others + -d.Control.ME  d[4,6] <- -d.AE.Control + d.AE.RE + -d.Control.ME  d[4,7] <- -d.AE.Control + d.AE.Stretching + -d.Control.ME  d[5,1] <- -d.AE.Others  d[5,2] <- d.AE.Control + -d.AE.Others  d[5,3] <- d.AE.MBE + -d.AE.Others  d[5,4] <- d.AE.Control + -d.AE.Others + d.Control.ME  d[5,5] <- 0  d[5,6] <- -d.AE.Others + d.AE.RE  d[5,7] <- -d.AE.Others + d.AE.Stretching  d[6,1] <- -d.AE.RE  d[6,2] <- d.AE.Control + -d.AE.RE  d[6,3] <- d.AE.MBE + -d.AE.RE  d[6,4] <- d.AE.Control + -d.AE.RE + d.Control.ME  d[6,5] <- d.AE.Others + -d.AE.RE  d[6,6] <- 0  d[6,7] <- -d.AE.RE + d.AE.Stretching  d[7,1] <- -d.AE.Stretching  d[7,2] <- d.AE.Control + -d.AE.Stretching  d[7,3] <- d.AE.MBE + -d.AE.Stretching  d[7,4] <- d.AE.Control + -d.AE.Stretching + d.Control.ME  d[7,5] <- d.AE.Others + -d.AE.Stretching  d[7,6] <- d.AE.RE + -d.AE.Stretching  d[7,7] <- 0  # Study baseline priors  for (i in 1:ns) {  mu[i] ~ dnorm(0, 8.889E-6)  }  # Variance prior  sd.d ~ dunif(0, 2.236E1)  tau.d <- pow(sd.d, -2)  # Effect parameter priors  d.AE.Control ~ dnorm(0, 8.889E-6)  d.AE.MBE ~ dnorm(0, 8.889E-6)  d.AE.Others ~ dnorm(0, 8.889E-6)  d.AE.RE ~ dnorm(0, 8.889E-6)  d.AE.Stretching ~ dnorm(0, 8.889E-6)  d.Control.ME ~ dnorm(0, 8.889E-6)  } |
| --- |

**3.2 WinBUGS Code for Network Meta-Analysis of Depression**

| model {  for (i in 1:ns) {  # Likelihood for each arm  for (k in 1:na[i]) {  m[i, k] ~ dnorm(theta[i, k], prec[i, k])  theta[i, k] <- mu[i] + delta[i, k]  prec[i, k] <- pow(e[i, k], -2)  }  # Study-level relative effects  # The arms are given in the order (arm_1, arm_2, ..., arm_{n_a-1}, arm_{n_a}).  # The relative effects are parameterized as d[arm_1, arm_k].  w[i, 1] <- 0  delta[i, 1] <- 0  for (k in 2:na[i]) { # parameterize multi-arm trials using a trick to avoid dmnorm  delta[i, k] ~ dnorm(md[i, k], taud[i, k])  md[i, k] <- d[t[i, 1], t[i, k]] + sw[i, k]  taud[i, k] <- tau.d * 2 * (k - 1) / k  w[i, k] <- delta[i, k] - d[t[i, 1], t[i, k]]  sw[i, k] <- sum(w[i, 1:k-1]) / (k - 1)  }  }  # Relative effect matrix  d[1,1] <- 0  d[1,2] <- d.AE.Control  d[1,3] <- d.AE.MBE  d[1,4] <- d.AE.Control + d.Control.ME  d[1,5] <- d.AE.Others  d[1,6] <- d.AE.RE  d[1,7] <- d.AE.Stretching  d[2,1] <- -d.AE.Control  d[2,2] <- 0  d[2,3] <- -d.AE.Control + d.AE.MBE  d[2,4] <- d.Control.ME  d[2,5] <- -d.AE.Control + d.AE.Others  d[2,6] <- -d.AE.Control + d.AE.RE  d[2,7] <- -d.AE.Control + d.AE.Stretching  d[3,1] <- -d.AE.MBE  d[3,2] <- d.AE.Control + -d.AE.MBE  d[3,3] <- 0  d[3,4] <- d.AE.Control + -d.AE.MBE + d.Control.ME  d[3,5] <- -d.AE.MBE + d.AE.Others  d[3,6] <- -d.AE.MBE + d.AE.RE  d[3,7] <- -d.AE.MBE + d.AE.Stretching  d[4,1] <- -d.AE.Control + -d.Control.ME  d[4,2] <- -d.Control.ME  d[4,3] <- -d.AE.Control + d.AE.MBE + -d.Control.ME  d[4,4] <- 0  d[4,5] <- -d.AE.Control + d.AE.Others + -d.Control.ME  d[4,6] <- -d.AE.Control + d.AE.RE + -d.Control.ME  d[4,7] <- -d.AE.Control + d.AE.Stretching + -d.Control.ME  d[5,1] <- -d.AE.Others  d[5,2] <- d.AE.Control + -d.AE.Others  d[5,3] <- d.AE.MBE + -d.AE.Others  d[5,4] <- d.AE.Control + -d.AE.Others + d.Control.ME  d[5,5] <- 0  d[5,6] <- -d.AE.Others + d.AE.RE  d[5,7] <- -d.AE.Others + d.AE.Stretching  d[6,1] <- -d.AE.RE  d[6,2] <- d.AE.Control + -d.AE.RE  d[6,3] <- d.AE.MBE + -d.AE.RE  d[6,4] <- d.AE.Control + -d.AE.RE + d.Control.ME  d[6,5] <- d.AE.Others + -d.AE.RE  d[6,6] <- 0  d[6,7] <- -d.AE.RE + d.AE.Stretching  d[7,1] <- -d.AE.Stretching  d[7,2] <- d.AE.Control + -d.AE.Stretching  d[7,3] <- d.AE.MBE + -d.AE.Stretching  d[7,4] <- d.AE.Control + -d.AE.Stretching + d.Control.ME  d[7,5] <- d.AE.Others + -d.AE.Stretching  d[7,6] <- d.AE.RE + -d.AE.Stretching  d[7,7] <- 0  # Study baseline priors  for (i in 1:ns) {  mu[i] ~ dnorm(0, 8.889E-6)  }  # Variance prior  sd.d ~ dunif(0, 2.236E1)  tau.d <- pow(sd.d, -2)  # Effect parameter priors  d.AE.Control ~ dnorm(0, 8.889E-6)  d.AE.MBE ~ dnorm(0, 8.889E-6)  d.AE.Others ~ dnorm(0, 8.889E-6)  d.AE.RE ~ dnorm(0, 8.889E-6)  d.AE.Stretching ~ dnorm(0, 8.889E-6)  d.Control.ME ~ dnorm(0, 8.889E-6)  } |
| --- |

**3.3 WinBUGS Code for Network Meta-Analysis of Anxiety Disorder**

| model {  for (i in 1:ns) {  # Likelihood for each arm  for (k in 1:na[i]) {  m[i, k] ~ dnorm(theta[i, k], prec[i, k])  theta[i, k] <- mu[i] + delta[i, k]  prec[i, k] <- pow(e[i, k], -2)  }  # Study-level relative effects  # The arms are given in the order (arm_1, arm_2, ..., arm_{n_a-1}, arm_{n_a}).  # The relative effects are parameterized as d[arm_1, arm_k].  w[i, 1] <- 0  delta[i, 1] <- 0  for (k in 2:na[i]) { # parameterize multi-arm trials using a trick to avoid dmnorm  delta[i, k] ~ dnorm(md[i, k], taud[i, k])  md[i, k] <- d[t[i, 1], t[i, k]] + sw[i, k]  taud[i, k] <- tau.d * 2 * (k - 1) / k  w[i, k] <- delta[i, k] - d[t[i, 1], t[i, k]]  sw[i, k] <- sum(w[i, 1:k-1]) / (k - 1)  }  }  # Relative effect matrix  d[1,1] <- 0  d[1,2] <- -d.Control.AE  d[1,3] <- -d.Control.AE + d.Control.MBE  d[1,4] <- -d.Control.AE + d.Control.RE  d[2,1] <- d.Control.AE  d[2,2] <- 0  d[2,3] <- d.Control.MBE  d[2,4] <- d.Control.RE  d[3,1] <- d.Control.AE + -d.Control.MBE  d[3,2] <- -d.Control.MBE  d[3,3] <- 0  d[3,4] <- -d.Control.MBE + d.Control.RE  d[4,1] <- d.Control.AE + -d.Control.RE  d[4,2] <- -d.Control.RE  d[4,3] <- d.Control.MBE + -d.Control.RE  d[4,4] <- 0  # Study baseline priors  for (i in 1:ns) {  mu[i] ~ dnorm(0, 8.573E-5)  }  # Variance prior  sd.d ~ dunif(0, 7.2E0)  tau.d <- pow(sd.d, -2)  # Effect parameter priors  d.Control.AE ~ dnorm(0, 8.573E-5)  d.Control.MBE ~ dnorm(0, 8.573E-5)  d.Control.RE ~ dnorm(0, 8.573E-5)  } |
| --- |

**3.4 WinBUGS Code for Network Meta-Analysis of Post-Traumatic Stress Disorder**

| model {  for (i in 1:ns) {  # Likelihood for each arm  for (k in 1:na[i]) {  m[i, k] ~ dnorm(theta[i, k], prec[i, k])  theta[i, k] <- mu[i] + delta[i, k]  prec[i, k] <- pow(e[i, k], -2)  }  # Study-level relative effects  # The arms are given in the order (arm_1, arm_2, ..., arm_{n_a-1}, arm_{n_a}).  # The relative effects are parameterized as d[arm_1, arm_k].  w[i, 1] <- 0  delta[i, 1] <- 0  for (k in 2:na[i]) { # parameterize multi-arm trials using a trick to avoid dmnorm  delta[i, k] ~ dnorm(md[i, k], taud[i, k])  md[i, k] <- d[t[i, 1], t[i, k]] + sw[i, k]  taud[i, k] <- tau.d * 2 * (k - 1) / k  w[i, k] <- delta[i, k] - d[t[i, 1], t[i, k]]  sw[i, k] <- sum(w[i, 1:k-1]) / (k - 1)  }  }  # Relative effect matrix  d[1,1] <- 0  d[1,2] <- d.Control.MBE  d[1,3] <- d.Control.ME  d[1,4] <- d.Control.RE  d[2,1] <- -d.Control.MBE  d[2,2] <- 0  d[2,3] <- -d.Control.MBE + d.Control.ME  d[2,4] <- -d.Control.MBE + d.Control.RE  d[3,1] <- -d.Control.ME  d[3,2] <- d.Control.MBE + -d.Control.ME  d[3,3] <- 0  d[3,4] <- -d.Control.ME + d.Control.RE  d[4,1] <- -d.Control.RE  d[4,2] <- d.Control.MBE + -d.Control.RE  d[4,3] <- d.Control.ME + -d.Control.RE  d[4,4] <- 0  # Study baseline priors  for (i in 1:ns) {  mu[i] ~ dnorm(0, 1.372E-5)  }  # Variance prior  sd.d ~ dunif(0, 1.8E1)  tau.d <- pow(sd.d, -2)  # Effect parameter priors  d.Control.MBE ~ dnorm(0, 1.372E-5)  d.Control.ME ~ dnorm(0, 1.372E-5)  d.Control.RE ~ dnorm(0, 1.372E-5)  } |
| --- |

**3.5 WinBUGS Code for Network Meta-Analysis of Overall Schizophrenic Symptom**

| model {  for (i in 1:ns) {  # Likelihood for each arm  for (k in 1:na[i]) {  m[i, k] ~ dnorm(theta[i, k], prec[i, k])  theta[i, k] <- mu[i] + delta[i, k]  prec[i, k] <- pow(e[i, k], -2)  }  # Study-level relative effects  # The arms are given in the order (arm_1, arm_2, ..., arm_{n_a-1}, arm_{n_a}).  # The relative effects are parameterized as d[arm_1, arm_k].  w[i, 1] <- 0  delta[i, 1] <- 0  for (k in 2:na[i]) { # parameterize multi-arm trials using a trick to avoid dmnorm  delta[i, k] ~ dnorm(md[i, k], taud[i, k])  md[i, k] <- d[t[i, 1], t[i, k]] + sw[i, k]  taud[i, k] <- tau.d * 2 * (k - 1) / k  w[i, k] <- delta[i, k] - d[t[i, 1], t[i, k]]  sw[i, k] <- sum(w[i, 1:k-1]) / (k - 1)  }  }  # Relative effect matrix  d[1,1] <- 0  d[1,2] <- d.AE.Control  d[1,3] <- d.AE.MBE  d[1,4] <- d.AE.Control + d.Control.ME  d[1,5] <- d.AE.Others  d[1,6] <- d.AE.Control + d.Control.RE  d[1,7] <- d.AE.Stretching  d[2,1] <- -d.AE.Control  d[2,2] <- 0  d[2,3] <- -d.AE.Control + d.AE.MBE  d[2,4] <- d.Control.ME  d[2,5] <- -d.AE.Control + d.AE.Others  d[2,6] <- d.Control.RE  d[2,7] <- -d.AE.Control + d.AE.Stretching  d[3,1] <- -d.AE.MBE  d[3,2] <- d.AE.Control + -d.AE.MBE  d[3,3] <- 0  d[3,4] <- d.AE.Control + -d.AE.MBE + d.Control.ME  d[3,5] <- -d.AE.MBE + d.AE.Others  d[3,6] <- d.AE.Control + -d.AE.MBE + d.Control.RE  d[3,7] <- -d.AE.MBE + d.AE.Stretching  d[4,1] <- -d.AE.Control + -d.Control.ME  d[4,2] <- -d.Control.ME  d[4,3] <- -d.AE.Control + d.AE.MBE + -d.Control.ME  d[4,4] <- 0  d[4,5] <- -d.AE.Control + d.AE.Others + -d.Control.ME  d[4,6] <- -d.Control.ME + d.Control.RE  d[4,7] <- -d.AE.Control + d.AE.Stretching + -d.Control.ME  d[5,1] <- -d.AE.Others  d[5,2] <- d.AE.Control + -d.AE.Others  d[5,3] <- d.AE.MBE + -d.AE.Others  d[5,4] <- d.AE.Control + -d.AE.Others + d.Control.ME  d[5,5] <- 0  d[5,6] <- d.AE.Control + -d.AE.Others + d.Control.RE  d[5,7] <- -d.AE.Others + d.AE.Stretching  d[6,1] <- -d.AE.Control + -d.Control.RE  d[6,2] <- -d.Control.RE  d[6,3] <- -d.AE.Control + d.AE.MBE + -d.Control.RE  d[6,4] <- d.Control.ME + -d.Control.RE  d[6,5] <- -d.AE.Control + d.AE.Others + -d.Control.RE  d[6,6] <- 0  d[6,7] <- -d.AE.Control + d.AE.Stretching + -d.Control.RE  d[7,1] <- -d.AE.Stretching  d[7,2] <- d.AE.Control + -d.AE.Stretching  d[7,3] <- d.AE.MBE + -d.AE.Stretching  d[7,4] <- d.AE.Control + -d.AE.Stretching + d.Control.ME  d[7,5] <- d.AE.Others + -d.AE.Stretching  d[7,6] <- d.AE.Control + -d.AE.Stretching + d.Control.RE  d[7,7] <- 0  # Study baseline priors  for (i in 1:ns) {  mu[i] ~ dnorm(0, 2.872E-5)  }  # Variance prior  sd.d ~ dunif(0, 1.244E1)  tau.d <- pow(sd.d, -2)  # Effect parameter priors  d.AE.Control ~ dnorm(0, 2.872E-5)  d.AE.MBE ~ dnorm(0, 2.872E-5)  d.AE.Others ~ dnorm(0, 2.872E-5)  d.AE.Stretching ~ dnorm(0, 2.872E-5)  d.Control.ME ~ dnorm(0, 2.872E-5)  d.Control.RE ~ dnorm(0, 2.872E-5)  } |
| --- |

**3.6 WinBUGS Code for Network Meta-Analysis of Positive Schizophrenic Symptom**

| model {  for (i in 1:ns) {  # Likelihood for each arm  for (k in 1:na[i]) {  m[i, k] ~ dnorm(theta[i, k], prec[i, k])  theta[i, k] <- mu[i] + delta[i, k]  prec[i, k] <- pow(e[i, k], -2)  }  # Study-level relative effects  # The arms are given in the order (arm_1, arm_2, ..., arm_{n_a-1}, arm_{n_a}).  # The relative effects are parameterized as d[arm_1, arm_k].  w[i, 1] <- 0  delta[i, 1] <- 0  for (k in 2:na[i]) { # parameterize multi-arm trials using a trick to avoid dmnorm  delta[i, k] ~ dnorm(md[i, k], taud[i, k])  md[i, k] <- d[t[i, 1], t[i, k]] + sw[i, k]  taud[i, k] <- tau.d * 2 * (k - 1) / k  w[i, k] <- delta[i, k] - d[t[i, 1], t[i, k]]  sw[i, k] <- sum(w[i, 1:k-1]) / (k - 1)  }  }  # Relative effect matrix  d[1,1] <- 0  d[1,2] <- d.AE.Control  d[1,3] <- d.AE.MBE  d[1,4] <- d.AE.Others  d[1,5] <- d.AE.Control + d.Control.RE  d[1,6] <- d.AE.Stretching  d[2,1] <- -d.AE.Control  d[2,2] <- 0  d[2,3] <- -d.AE.Control + d.AE.MBE  d[2,4] <- -d.AE.Control + d.AE.Others  d[2,5] <- d.Control.RE  d[2,6] <- -d.AE.Control + d.AE.Stretching  d[3,1] <- -d.AE.MBE  d[3,2] <- d.AE.Control + -d.AE.MBE  d[3,3] <- 0  d[3,4] <- -d.AE.MBE + d.AE.Others  d[3,5] <- d.AE.Control + -d.AE.MBE + d.Control.RE  d[3,6] <- -d.AE.MBE + d.AE.Stretching  d[4,1] <- -d.AE.Others  d[4,2] <- d.AE.Control + -d.AE.Others  d[4,3] <- d.AE.MBE + -d.AE.Others  d[4,4] <- 0  d[4,5] <- d.AE.Control + -d.AE.Others + d.Control.RE  d[4,6] <- -d.AE.Others + d.AE.Stretching  d[5,1] <- -d.AE.Control + -d.Control.RE  d[5,2] <- -d.Control.RE  d[5,3] <- -d.AE.Control + d.AE.MBE + -d.Control.RE  d[5,4] <- -d.AE.Control + d.AE.Others + -d.Control.RE  d[5,5] <- 0  d[5,6] <- -d.AE.Control + d.AE.Stretching + -d.Control.RE  d[6,1] <- -d.AE.Stretching  d[6,2] <- d.AE.Control + -d.AE.Stretching  d[6,3] <- d.AE.MBE + -d.AE.Stretching  d[6,4] <- d.AE.Others + -d.AE.Stretching  d[6,5] <- d.AE.Control + -d.AE.Stretching + d.Control.RE  d[6,6] <- 0  # Study baseline priors  for (i in 1:ns) {  mu[i] ~ dnorm(0, 2.602E-5)  }  # Variance prior  sd.d ~ dunif(0, 1.307E1)  tau.d <- pow(sd.d, -2)  # Effect parameter priors  d.AE.Control ~ dnorm(0, 2.602E-5)  d.AE.MBE ~ dnorm(0, 2.602E-5)  d.AE.Others ~ dnorm(0, 2.602E-5)  d.AE.Stretching ~ dnorm(0, 2.602E-5)  d.Control.RE ~ dnorm(0, 2.602E-5)  } |
| --- |

**3.7 WinBUGS Code for Network Meta-Analysis of Negative Schizophrenic Symptom**

| model {  for (i in 1:ns) {  # Likelihood for each arm  for (k in 1:na[i]) {  m[i, k] ~ dnorm(theta[i, k], prec[i, k])  theta[i, k] <- mu[i] + delta[i, k]  prec[i, k] <- pow(e[i, k], -2)  }  # Study-level relative effects  # The arms are given in the order (arm_1, arm_2, ..., arm_{n_a-1}, arm_{n_a}).  # The relative effects are parameterized as d[arm_1, arm_k].  w[i, 1] <- 0  delta[i, 1] <- 0  for (k in 2:na[i]) { # parameterize multi-arm trials using a trick to avoid dmnorm  delta[i, k] ~ dnorm(md[i, k], taud[i, k])  md[i, k] <- d[t[i, 1], t[i, k]] + sw[i, k]  taud[i, k] <- tau.d * 2 * (k - 1) / k  w[i, k] <- delta[i, k] - d[t[i, 1], t[i, k]]  sw[i, k] <- sum(w[i, 1:k-1]) / (k - 1)  }  }  # Relative effect matrix  d[1,1] <- 0  d[1,2] <- d.AE.Control  d[1,3] <- d.AE.MBE  d[1,4] <- d.AE.Control + d.Control.ME  d[1,5] <- d.AE.Others  d[1,6] <- d.AE.Control + d.Control.RE  d[1,7] <- d.AE.Stretching  d[2,1] <- -d.AE.Control  d[2,2] <- 0  d[2,3] <- -d.AE.Control + d.AE.MBE  d[2,4] <- d.Control.ME  d[2,5] <- -d.AE.Control + d.AE.Others  d[2,6] <- d.Control.RE  d[2,7] <- -d.AE.Control + d.AE.Stretching  d[3,1] <- -d.AE.MBE  d[3,2] <- d.AE.Control + -d.AE.MBE  d[3,3] <- 0  d[3,4] <- d.AE.Control + -d.AE.MBE + d.Control.ME  d[3,5] <- -d.AE.MBE + d.AE.Others  d[3,6] <- d.AE.Control + -d.AE.MBE + d.Control.RE  d[3,7] <- -d.AE.MBE + d.AE.Stretching  d[4,1] <- -d.AE.Control + -d.Control.ME  d[4,2] <- -d.Control.ME  d[4,3] <- -d.AE.Control + d.AE.MBE + -d.Control.ME  d[4,4] <- 0  d[4,5] <- -d.AE.Control + d.AE.Others + -d.Control.ME  d[4,6] <- -d.Control.ME + d.Control.RE  d[4,7] <- -d.AE.Control + d.AE.Stretching + -d.Control.ME  d[5,1] <- -d.AE.Others  d[5,2] <- d.AE.Control + -d.AE.Others  d[5,3] <- d.AE.MBE + -d.AE.Others  d[5,4] <- d.AE.Control + -d.AE.Others + d.Control.ME  d[5,5] <- 0  d[5,6] <- d.AE.Control + -d.AE.Others + d.Control.RE  d[5,7] <- -d.AE.Others + d.AE.Stretching  d[6,1] <- -d.AE.Control + -d.Control.RE  d[6,2] <- -d.Control.RE  d[6,3] <- -d.AE.Control + d.AE.MBE + -d.Control.RE  d[6,4] <- d.Control.ME + -d.Control.RE  d[6,5] <- -d.AE.Control + d.AE.Others + -d.Control.RE  d[6,6] <- 0  d[6,7] <- -d.AE.Control + d.AE.Stretching + -d.Control.RE  d[7,1] <- -d.AE.Stretching  d[7,2] <- d.AE.Control + -d.AE.Stretching  d[7,3] <- d.AE.MBE + -d.AE.Stretching  d[7,4] <- d.AE.Control + -d.AE.Stretching + d.Control.ME  d[7,5] <- d.AE.Others + -d.AE.Stretching  d[7,6] <- d.AE.Control + -d.AE.Stretching + d.Control.RE  d[7,7] <- 0  # Study baseline priors  for (i in 1:ns) {  mu[i] ~ dnorm(0, 7.748E-6)  }  # Variance prior  sd.d ~ dunif(0, 2.395E1)  tau.d <- pow(sd.d, -2)  # Effect parameter priors  d.AE.Control ~ dnorm(0, 7.748E-6)  d.AE.MBE ~ dnorm(0, 7.748E-6)  d.AE.Others ~ dnorm(0, 7.748E-6)  d.AE.Stretching ~ dnorm(0, 7.748E-6)  d.Control.ME ~ dnorm(0, 7.748E-6)  d.Control.RE ~ dnorm(0, 7.748E-6)  } |
| --- |
