# Supplementary material for: Comparative Effectiveness of Multiple Exercise Interventions in the Treatment of Mental Health Disorders: A Systematic Review and Network Meta-Analysis
Source: Sports Med Open. 2022 Oct 29;8:135. doi: 10.1186/s40798-022-00529-5 (PMC9617247; doi:10.1186/s40798-022-00529-5)
Supplement: Supplementary file 5 — Additional file 5: Appendix 4. Included Studies List. [file 40798_2022_529_MOESM5_ESM.docx]

**Appendix 4.** Included Studies List

**[Notes.** depression[1-70], anxiety disorder [71-75], post‐traumatic stress disorder [76-86], schizophrenia [87-117]]

**References:**

1. Doyne, E.J., et al., Running versus weight lifting in the treatment of depression. J Consult Clin Psychol, 1987. **55**(5): p. 748-54.

2. McNeil, J.K., E.M. LeBlanc, and M. Joyner, The effect of exercise on depressive symptoms in the moderately depressed elderly. Psychol Aging, 1991. **6**(3): p. 487-8.

3. Veale, D., et al., Aerobic exercise in the adjunctive treatment of depression: a randomized controlled trial. J R Soc Med, 1992. **85**(9): p. 541-4.

4. Singh, N.A., K.M. Clements, and M.A. Fiatarone, A randomized controlled trial of progressive resistance training in depressed elders. J Gerontol A Biol Sci Med Sci, 1997. **52**(1): p. M27-35.

5. Blumenthal, J.A., et al., Effects of exercise training on older patients with major depression. Archives of Internal Medicine, 1999. **159**(19): p. 2349-2356.

6. Armstrong, K. and H. Edwards, The effects of exercise and social support on mothers reporting depressive symptoms: a pilot randomized controlled trial. Int J Ment Health Nurs, 2003. **12**(2): p. 130-8.

7. Sharma, V.K., et al., Effect of Sahaj Yoga on depressive disorders. Indian J Physiol Pharmacol, 2005. **49**(4): p. 462-8.

8. Singh, N.A., et al., A randomized controlled trial of high versus low intensity weight training versus general practitioner care for clinical depression in older adults. Journals of Gerontology Series A: Biological Sciences & Medical Sciences, 2005. **60**(6): p. 768-776.

9. Blumenthal, J.A., et al., Exercise and pharmacotherapy in the treatment of major depressive disorder. PSYCHOSOMATIC MEDICINE, 2007. **69**(7): p. 587-596.

10. Brenes, G.A., et al., Treatment of minor depression in older adults: A pilot study comparing sertraline and exercise. Aging & Mental Health, 2007. **11**(1): p. 61-68.

11. Knubben, K., et al., A randomised, controlled study on the effects of a short-term endurance training programme in patients with major depression. Br J Sports Med, 2007. **41**(1): p. 29-33.

12. Daley, A., et al., Feasibility of an exercise intervention for women with postnatal depression: a pilot randomised controlled trial. Br J Gen Pract, 2008. **58**(548): p. 178-83.

13. Heh, S., et al., Effectiveness of an exercise support program in reducing the severity of postnatal depression in Taiwanese women. Birth: Issues in Perinatal Care, 2008. **35**(1): p. 60-65.

14. Da Costa, D., et al., A randomized clinical trial of exercise to alleviate postpartum depressed mood. J Psychosom Obstet Gynaecol, 2009. **30**(3): p. 191-200.

15. Krogh, J., et al., The DEMO trial: a randomized, parallel-group, observer-blinded clinical trial of strength versus aerobic versus relaxation training for patients with mild to moderate depression. J Clin Psychiatry, 2009. **70**(6): p. 790-800.

16. Callaghan, P., et al., Pragmatic randomised controlled trial of preferred intensity exercise in women living with depression. BMC Public Health, 2011. **11**: p. 465.

17. de la Cerda, P., et al., Effect of an aerobic training program as complementary therapy in patients with moderate depression. Percept Mot Skills, 2011. **112**(3): p. 761-9.

18. Lavretsky, H., et al., Complementary use of tai chi chih augments escitalopram treatment of geriatric depression: a randomized controlled trial. Am J Geriatr Psychiatry, 2011. **19**(10): p. 839-50.

19. Mota-Pereira, J., et al., Moderate exercise improves depression parameters in treatment-resistant patients with major depressive disorder. J Psychiatr Res, 2011. **45**(8): p. 1005-11.

20. Roshan, V.D., M. Pourasghar, and Z. Mohammadian, The efficacy of intermittent walking in water on the rate of MHPG sulfate and the severity of depression. Iranian journal of psychiatry and behavioral sciences, 2011. **5**(2): p. 26‐31.

21. Schuch, F.B., et al., Exercise and severe depression: preliminary results of an add-on study. J Affect Disord, 2011. **133**(3): p. 615-8.

22. Hemat-Far, A., A. Shahsavari, and S.R. Mousavi, Effects of selected aerobic exercises on the depression and concentrations of plasma serotonin in the depressed female students aged 18 to 25. Journal of Applied Research, 2012. **12**(1): p. 47-52.

23. Krogh, J., et al., DEMO-II trial. Aerobic exercise versus stretching exercise in patients with major depression-a randomised clinical trial. PLoS One, 2012. **7**(10): p. e48316.

24. Mitchell, J., et al., Yoga reduces prenatal depression symptoms. Psychology, 2012. **3**(9A): p. 782-786.

25. Yeung, A., et al., Tai chi treatment for depression in Chinese Americans: a pilot study. Am J Phys Med Rehabil, 2012. **91**(10): p. 863-70.

26. Field, T., et al., Tai chi/yoga reduces prenatal depression, anxiety and sleep disturbances. Complement Ther Clin Pract, 2013. **19**(1): p. 6-10.

27. Field, T., et al., Yoga and social support reduce prenatal depression, anxiety and cortisol. J Bodyw Mov Ther, 2013. **17**(4): p. 397-403.

28. Gangadhar, B.N., et al., Positive antidepressant effects of generic yoga in depressive out-patients: A comparative study. Indian Journal of Psychiatry, 2013. **55**(7): p. S369-S373.

29. Naveen, G.H., et al., Positive therapeutic and neurotropic effects of yoga in depression: A comparative study. Indian Journal of Psychiatry, 2013. **55**(7): p. S400-S404.

30. Ho, C.W.H., et al., Effect of Aerobic Exercise Training on Chinese Population with Mild to Moderate Depression in Hong Kong. Rehabilitation Research & Practice, 2014: p. 1-8.

31. Sarubin, N., et al., The influence of Hatha yoga as an add-on treatment in major depression on hypothalamic-pituitary-adrenal-axis activity: A randomized trial. Journal of Psychiatric Research, 2014. **53**: p. 76-83.

32. Belvederi Murri, M., et al., Physical exercise for late-life major depression. Br J Psychiatry, 2015. **207**(3): p. 235-42.

33. Buttner, M.M., et al., Efficacy of yoga for depressed postpartum women: A randomized controlled trial. Complement Ther Clin Pract, 2015. **21**(2): p. 94-100.

34. Carter, T., et al., Preferred intensity exercise for adolescents receiving treatment for depression: a pragmatic randomised controlled trial. BMC Psychiatry, 2015. **15**: p. 247.

35. Doose, M., et al., Self-selected intensity exercise in the treatment of major depression: A pragmatic RCT. Int J Psychiatry Clin Pract, 2015. **19**(4): p. 266-75.

36. Kerling, A., et al., Effects of adjunctive exercise on physiological and psychological parameters in depression: a randomized pilot trial. Journal of affective disorders, 2015. **177**: p. 1‐6.

37. Majumder, P., et al., The effect of aerobic exercise in the maintenance treatment of depression. Bjpsych international, 2015. **12**(S1): p. S3‐S6.

38. Schuch, F.B., et al., Exercise and severe major depression: effect on symptom severity and quality of life at discharge in an inpatient cohort. J Psychiatr Res, 2015. **61**: p. 25-32.

39. Legrand, F.D. and E.M. Neff, Efficacy of exercise as an adjunct treatment for clinically depressed inpatients during the initial stages of antidepressant pharmacotherapy: An open randomized controlled trial. J Affect Disord, 2016. **191**: p. 139-44.

40. Schuver, K.J. and B.A. Lewis, Mindfulness-based yoga intervention for women with depression. Complement Ther Med, 2016. **26**: p. 85-91.

41. Siqueira, C.C., et al., Antidepressant Efficacy of Adjunctive Aerobic Activity and Associated Biomarkers in Major Depression: A 4-Week, Randomized, Single-Blind, Controlled Clinical Trial. PLoS One, 2016. **11**(5): p. e0154195.

42. Toni, G., et al., Physical Exercise for Late-Life Depression: effects on Heart Rate Variability. American journal of geriatric psychiatry, 2016. **24**(11): p. 989‐997.

43. Forsyth, J., et al., Exercise as an adjunct treatment for postpartum depression for women living in an inner city-A pilot study. Health Care Women Int, 2017. **38**(6): p. 635-639.

44. Olson, R.L., et al., A randomized trial of aerobic exercise on cognitive control in major depression. Clin Neurophysiol, 2017. **128**(6): p. 903-913.

45. Prathikanti, S., et al., Treating major depression with yoga: A prospective, randomized, controlled pilot trial. PLoS One, 2017. **12**(3): p. e0173869.

46. Turner, D., et al., Cost-effectiveness of a preferred intensity exercise programme for young people with depression compared with treatment as usual: an economic evaluation alongside a clinical trial in the UK. BMJ Open, 2017. **7**(11): p. e016211.

47. Uebelacker, L.A., et al., Adjunctive yoga v. health education for persistent major depression: a randomized controlled trial. Psychol Med, 2017. **47**(12): p. 2130-2142.

48. Yeung, A.S., et al., A Pilot, Randomized Controlled Study of Tai Chi With Passive and Active Controls in the Treatment of Depressed Chinese Americans. J Clin Psychiatry, 2017. **78**(5): p. e522-e528.

49. Cheung, L.K. and S. Lee, A randomized controlled trial on an aerobic exercise programme for depression outpatients. Sport Sciences for Health, 2018. **14**(1): p. 173-181.

50. Gerber, M., et al., Sprint Interval Training and Continuous Aerobic Exercise Training Have Similar Effects on Exercise Motivation and Affective Responses to Exercise in Patients With Major Depressive Disorders: A Randomized Controlled Trial. Frontiers in Psychiatry, 2018. **9**.

51. Minghetti, A., et al., Sprint interval training (SIT) substantially reduces depressive symptoms in major depressive disorder (MDD): A randomized controlled trial. Psychiatry Res, 2018. **265**: p. 292-297.

52. Tolahunase, M.R., R. Sagar, and R. Dada, 5-HTTLPR and MTHFR 677C > T polymorphisms and response to yoga-based lifestyle intervention in major depressive disorder: Arandomized active-controlled trial. Indian Journal of Psychiatry, 2018. **60**(4): p. 410-426.

53. Bressington, D., et al., Feasibility of a group-based laughter yoga intervention as an adjunctive treatment for residual symptoms of depression, anxiety and stress in people with depression. J Affect Disord, 2019. **248**: p. 42-51.

54. Kumar, S., et al., Effect of adjunct yoga therapy in depressive disorders: Findings from a randomized controlled study. Indian Journal of Psychiatry, 2019. **61**(6): p. 592-597.

55. Tasci, G., et al., Effect of exercise on therapeutic response in depression treatment. Psychiatry and Clinical Psychopharmacology, 2019. **29**(2): p. 137-143.

56. Zhang, J.L. and T.X. Chen, Effect of Aerobic Exercise on Cognitive Function and Symptoms in Patients with Depression. National Academy Science Letters-India, 2019. **42**(5): p. 419-421.

57. Chau, R.M.W., et al., Effectiveness of a structured physical rehabilitation program on the physical fitness, mental health and pain for Chinese patients with major depressive disorders in Hong Kong - a randomized controlled trial with 9-month follow-up outcomes. Disability and Rehabilitation, 2020.

58. Gerber, M., et al., Effects of Aerobic Exercise on Cortisol Stress Reactivity in Response to the Trier Social Stress Test in Inpatients with Major Depressive Disorders: A Randomized Controlled Trial. Journal of Clinical Medicine, 2020. **9**(5).

59. Hyvonen, K., et al., The Effects of Dance Movement Therapy in the Treatment of Depression: A Multicenter, Randomized Controlled Trial in Finland. Frontiers in Psychology, 2020. **11**.

60. Imboden, C., et al., Aerobic exercise or stretching as add-on to inpatient treatment of depression: Similar antidepressant effects on depressive symptoms and larger effects on working memory for aerobic exercise alone. J Affect Disord, 2020. **276**: p. 866-876.

61. Ozkan, S.A., et al., The effectiveness of an exercise intervention in reducing the severity of postpartum depression: A randomized controlled trial. Perspectives in Psychiatric Care, 2020. **56**(4): p. 844-850.

62. Rao, U.T., J.A. Noronha, and K. Adiga, Effect of aerobic exercises on depressive symptoms, anxiety, self-esteem, and quality of life among adults with depression. Clinical Epidemiology and Global Health, 2020. **8**(4): p. 1147-1151.

63. Adagide, S. and N. Karatas, The effects of physical exercise on the depressive symptoms and quality of life of individuals diagnosed with depression. Journal of Psychiatric Nursing, 2021. **12**(2): p. 122-131.

64. Bieber, M., et al., Effects of body-oriented yoga: a RCT study for patients with major depressive disorder. European Archives of Psychiatry and Clinical Neuroscience, 2021. **271**(7): p. 1217-1229.

65. Bruchle, W., et al., Physical Activity Reduces Clinical Symptoms and Restores Neuroplasticity in Major Depression. Frontiers in Psychiatry, 2021. **12**.

66. Kang, H. and S. Jang, Effect of Mindfulness Yoga on Depression Severity, Self-Esteem, and Quality of Life in Middle-Aged Men. Iranian Journal of Public Health, 2021. **50**(7): p. 1334-1342.

67. Lavretsky, H., et al., A randomized controlled trial of tai chi chih or health education for geriatric depression. The American Journal of Geriatric Psychiatry, 2021.

68. Lewis, B.A., et al., Randomized trial examining the effect of exercise and wellness interventions on preventing postpartum depression and perceived stress. BMC Pregnancy Childbirth, 2021. **21**(1): p. 785.

69. Ravindran, A.V., et al., Breathing-focused Yoga as Augmentation for Unipolar and Bipolar Depression: A Randomized Controlled Trial: Le yoga axe sur la respiration comme traitement d'appoint pour la depression unipolaire et bipolaire: Un essai randomise controle. Canadian Journal of Psychiatry-Revue Canadienne De Psychiatrie, 2021. **66**(2): p. 159-169.

70. Srivastava, A., et al., Kriya Yoga in Patients with Depressive Disorders: A Pilot Study. Journal of Neurosciences in Rural Practice, 2021. **12**(02): p. 362-367.

71. Broocks, A., et al., Comparison of aerobic exercise, clomipramine, and placebo in the treatment of panic disorder. Am J Psychiatry, 1998. **155**(5): p. 603-9.

72. Herring, M.P., et al., Effects of short-term exercise training on signs and symptoms of generalized anxiety disorder. Mental Health and Physical Activity, 2011. **4**(2): p. 71-77.

73. Song, Q.H., et al., Effect of Tai Chi exercise on the physical and mental health of the elder patients suffered from anxiety disorder. International journal of physiology, pathophysiology and pharmacology, 2014. **6**(1): p. 55‐60.

74. Ma, W.-F., et al., The Effects of an Exercise Program on Anxiety Levels and Metabolic Functions in Patients With Anxiety Disorders. Biological Research for Nursing, 2017. **19**(3): p. 258-268.

75. Gordon, B.R., et al., Resistance exercise training among young adults with analogue generalized anxiety disorder. J Affect Disord, 2021. **281**: p. 153-159.

76. J.Carter, J., et al., Multi-Component Yoga Breath Program for Vietnam Veteran Post Traumatic Stress Disorder: Randomized Controlled Trial. Journal of Traumatic Stress disorders & Treatment, 2013. **2**: p. 1-10.

77. Mitchell, K.S., et al., A pilot study of a randomized controlled trial of yoga as an intervention for PTSD symptoms in women. J Trauma Stress, 2014. **27**(2): p. 121-8.

78. Thordardottir, K., et al., Effects of yoga practice on stress-related symptoms in the aftermath of an earthquake: A community-based controlled trial. Complement Ther Med, 2014. **22**(2): p. 226-34.

79. van der Kolk, B.A., et al., Yoga as an adjunctive treatment for posttraumatic stress disorder: a randomized controlled trial. J Clin Psychiatry, 2014. **75**(6): p. e559-65.

80. Jindani, F., N. Turner, and S.B. Khalsa, A Yoga Intervention for Posttraumatic Stress: A Preliminary Randomized Control Trial. Evid Based Complement Alternat Med, 2015. **2015**: p. 351746.

81. Quinones, N., et al., Efficacy of a Satyananda Yoga Intervention for Reintegrating Adults Diagnosed with Posttraumatic Stress Disorder. Int J Yoga Therap, 2015. **25**(1): p. 89-99.

82. Rosenbaum, S., C. Sherrington, and A. Tiedemann, Exercise augmentation compared with usual care for post-traumatic stress disorder: a randomized controlled trial. Acta Psychiatr Scand, 2015. **131**(5): p. 350-9.

83. Goldstein, L.A., et al., Veterans Group Exercise: A randomized pilot trial of an Integrative Exercise program for veterans with posttraumatic stress. J Affect Disord, 2018. **227**: p. 345-352.

84. Whitworth, J.W., et al., High intensity resistance training improves sleep quality and anxiety in individuals who screen positive for posttraumatic stress disorder: A randomized controlled feasibility trial. MENTAL HEALTH AND PHYSICAL ACTIVITY, 2019. **16**: p. 43-49.

85. Hall, K.S., et al., Pilot randomized controlled trial of exercise training for older veterans with PTSD. J Behav Med, 2020. **43**(4): p. 648-659.

86. Nguyen-Feng, V.N., et al., Moderators of treatment efficacy in a randomized controlled trial of trauma-sensitive yoga as an adjunctive treatment for posttraumatic stress disorder. Psychol Trauma, 2020. **12**(8): p. 836-846.

87. Duraiswamy, G., et al., Yoga therapy as an add-on treatment in the management of patients with schizophrenia--a randomized controlled trial. Acta Psychiatr Scand, 2007. **116**(3): p. 226-32.

88. Acil, A.A., S. Dogan, and O. Dogan, The effects of physical exercises to mental state and quality of life in patients with schizophrenia. J Psychiatr Ment Health Nurs, 2008. **15**(10): p. 808-15.

89. Behere, R.V., et al., Effect of yoga therapy on facial emotion recognition deficits, symptoms and functioning in patients with schizophrenia. Acta Psychiatr Scand, 2011. **123**(2): p. 147-53.

90. Ho, R.T.H., et al., Tai-Chi for residential patients with schizophrenia on movement coordination, negative symptoms, and functioning: a pilot randomized controlled trial. Evidence-based complementary and alternative medicine, 2012. **2012**.

91. Takahashi, H., et al., Effects of sports participation on psychiatric symptoms and brain activations during sports observation in schizophrenia. Transl Psychiatry, 2012. **2**(3): p. e96.

92. Varambally, S., et al., Therapeutic efficacy of add-on yogasana intervention in stabilized outpatient schizophrenia: randomized controlled comparison with exercise and waitlist. Indian journal of psychiatry, 2012. **54**(3): p. 227‐232.

93. Ikai, S., et al., Effects of yoga therapy on postural stability in patients with schizophrenia-spectrum disorders: a single-blind randomized controlled trial. J Psychiatr Res, 2013. **47**(11): p. 1744-50.

94. Jayaram, N., et al., Effect of yoga therapy on plasma oxytocin and facial emotion recognition deficits in patients of schizophrenia. Indian Journal of Psychiatry, 2013. **55**(7): p. S409-S413.

95. Ikai, S., et al., Effects of weekly one-hour Hatha yoga therapy on resilience and stress levels in patients with schizophrenia-spectrum disorders: an eight-week randomized controlled trial. J Altern Complement Med, 2014. **20**(11): p. 823-30.

96. Kaltsatou, A., et al., Effects of exercise training with traditional dancing on functional capacity and quality of life in patients with schizophrenia: a randomized controlled study. Clin Rehabil, 2015. **29**(9): p. 882-91.

97. Lee, H.J., et al., Effectiveness of dance/movement therapy on affect and psychotic symptoms in patients with schizophrenia. Arts in Psychotherapy, 2015. **45**: p. 64-68.

98. Loh, S.Y., et al., Structured Walking and Chronic Institutionalized Schizophrenia Inmates: A pilot RCT Study on Quality of Life. Glob J Health Sci, 2015. **8**(1): p. 238-48.

99. Paikkatt, B., et al., Efficacy of Yoga therapy for the management of psychopathology of patients having chronic schizophrenia. Indian Journal of Psychiatry, 2015. **57**(4): p. 355-360.

100. Silva, B.A., et al., A 20-week program of resistance or concurrent exercise improves symptoms of schizophrenia: results of a blind, randomized controlled trial. Revista brasileira de psiquiatria (Sao Paulo, Brazil : 1999), 2015. **37**(4): p. 271‐279.

101. Areshtanab, H.N., et al., The effect of regular aerobic exercise on both positive and negative symptoms of male patients with chronic Schizophrenia: A double blinded study. International Journal of Medical Research & Health Sciences, 2016. **5**(11): p. 529-535.

102. Ho Rainbow, T.H., et al., A randomized controlled trial on the psychophysiological effects of physical exercise and tai-chi in patients with chronic schizophrenia. Schizophren res, 2016: p. No‐Specified.

103. Kang, R., et al., Effect of Community-Based Social Skills Training and Tai-Chi Exercise on Outcomes in Patients with Chronic Schizophrenia: A Randomized, One-Year Study. Psychopathology, 2016. **49**(5): p. 345-355.

104. Kavak, F. and M. Ekinci, The Effect of Yoga on Functional Recovery Level in Schizophrenic Patients. Archives of Psychiatric Nursing, 2016. **30**(6): p. 761-767.

105. Martin, L.A., et al., Overcoming Disembodiment: the Effect of Movement Therapy on Negative Symptoms in Schizophrenia-A Multicenter Randomized Controlled Trial. Frontiers in psychology, 2016. **7**: p. 483.

106. Su, C.-Y., et al., The effects of aerobic exercise on cognition in schizophrenia: A 3-month follow-up study. Psychiatry Research, 2016. **244**: p. 394-402.

107. Curcic, D., et al., Positive impact of prescribed physical activity on symptoms of schizophrenia: randomized clinical trial. Psychiatr Danub, 2017. **29**(4): p. 459-465.

108. Wang, P.-W., et al., Effect of aerobic exercise on improving symptoms of individuals with schizophrenia: A single blinded randomized control study. Frontiers in Psychiatry, 2018. **9**.

109. Shimada, T., et al., Aerobic exercise and cognitive functioning in schizophrenia: A pilot randomized controlled trial. Psychiatry Res, 2019. **282**: p. 112638.

110. Bryl, K., et al., The role of dance/movement therapy in the treatment of negative symptoms in schizophrenia: a mixed methods pilot study. Journal of mental health (Abingdon, England), 2020.

111. Shimada, T., et al., Aerobic exercise and cognitive functioning in schizophrenia: Results of a 1-year follow-up from a randomized controlled trial. Psychiatry Research, 2020. **286**.

112. Akbas, E., et al., Effects of Pilates-Based Exercises on Functional Capacity and Mental Health in Individuals with Schizophrenia: a Pilot Study. Physiotherapy theory and practice, 2021: p. 1‐9.

113. Gao, H., et al., The Effect of Yijinjing on the Cognitive Function of Patients With Chronic Schizophrenia. Frontiers in Psychiatry, 2021. **12**.

114. Govindaraj, R., et al., Yoga therapy for social cognition in schizophrenia: An experimental medicine-based randomized controlled trial. Asian J Psychiatr, 2021. **62**: p. 102731.

115. Lo, L.L.H., et al., Effect of high-endurance exercise intervention on sleep-dependent procedural memory consolidation in individuals with schizophrenia: A randomized controlled trial. Psychological Medicine, 2021.

116. Rao, N.P., et al., Add on yoga treatment for negative symptoms of schizophrenia: a multi-centric, randomized controlled trial. Schizophrenia research, 2021. **231**: p. 90‐97.

117. Senormanci, G., et al., Effects of Exercise on Resilience, Insight and Functionality in Patients with Chronic Schizophrenia in a Psychiatric Nursing Home Setting: A Randomized Controlled Trial. Issues in Mental Health Nursing, 2021. **42**(7): p. 690-698.
