# Supplementary material for: Comparative Effectiveness of Multiple Exercise Interventions in the Treatment of Mental Health Disorders: A Systematic Review and Network Meta-Analysis
Source: Sports Med Open. 2022 Oct 29;8:135. doi: 10.1186/s40798-022-00529-5 (PMC9617247; doi:10.1186/s40798-022-00529-5)
Supplement: Supplementary file 6 — Additional file 6: Appendix 5. Basic Information of Included Studies. [file 40798_2022_529_MOESM6_ESM.docx]

**Appendix 5. Basic Information of Included Studies**

| **No** | **Study** | **Disorder** | **Sample Size** | **Age** | **Experimental Group** | | | | | | **Comparison** | **Outcome** |
| --- | --- | --- | --- | --- | --- | --- | --- | --- | --- | --- | --- | --- |
|  |  |  |  |  | **Type** | **Frequency** | **Session Duration** | **Length** | **Intensity** | **Supervision** |  |  |
| 1 | Doyne et al., 1987 | Depression | 13 vs.13 | 28.58 ± 3.48 y vs. 27.67 ± 4.81 y | AE (running) | 4 d/wk | / | 8 weeks | 80% HRmax | Yes | RE (weight-lifting) | BDI HRSD |
| 2 | McNeil et al., 1991 | Depression | 10 vs. 10 | 72.50 ± 6.90 y | AE (walking) | 3d/wk | 40min | 6 weeks | / | No | Usual Care | BDI |
| 3 | Veale et al., 1992 | Depression | 36 vs. 29 | 19-59 y | AE (running) | 3d/wk | / | 12 weeks | / | Yes | Usual Care | BDI |
| 4 | Singh et al., 1997 | Depression | 17 vs. 15 | 70 ± 1.5 y vs. 72 ± 2.0 y | RE | 3 d/wk | 45 min | 10 weeks | / | Yes | Health Education | BDI |
| 5 | Blumenthal et al., 1999 | Depression | 44 vs. 41 | 57.00 ± 6.70 y vs. 57.00 ± 7.00 y | AE (walking + jogging) | 3 d/wk | 45 min | 16 weeks | 70-80% HRmax | Yes | Usual Care | HRSD BDI |
| 6 | Armstrong et al., 2003 | Depression | 10 vs. 10 | 21-30 y | AE (walking) | 3 d/wk | 35 min | 12 weeks | 60-75% HRmax | Yes | Usual Care | EPDS |
| 7 | Sharma et al., 2005 | Depression | 15 vs. 15 | 31.87 ± 8.78 y vs. 31.67 ± 8.46 y | MBE (Yoga) | 3 d/wk | 30 min | 8 weeks | / | Yes | Usual Care | HRSD |
| 8 | Singh et al., 2005 | Depression | 19 vs. 18 | 69.00 ± 5.00 y vs. 69.00 ± 7.00 y | RE | 3 d/wk | 65 min | 8 weeks | 80% of one repetition maximum (1RM) | Yes | Usual Care | HRSD |
| 9 | Blumenthal et al., 2007 | Depression | 51 vs. 49 | 52 ± 7 y vs. 52 ± 8y | AE (Walking) | 3 d/wk | 45 min | 16 weeks | 70-85% HRmax | Yes | Usual Care | HRSD |
| 10 | Brenes et al., 2007 | Depression | 14 vs. 12 | 73.5 ± 7.8 vs. 73.9 ± 5.8 | ME (AE + RE) | 3 d/wk | 60 min | 16 weeks | AE: 50-85% of Hrmax | Yes | Usual Care | HRSD |
| 11 | Knubben et al., 2007 | Depression | 20 vs. 18 | 49.00 ± 13.00 vs. 50.00 ± 13.00 | AE (walking) | 7 d/wk | 30 min | 10 days | / | Yes | Stretching | BRMS CES-D |
| 12 | Daley et al., 2008 | Depression | 16 vs. 15 | 21-40 y | AE | 5 d/wk | 30 min | 12 weeks | / | No | Usual Care | EPDS |
| 13 | Heh et al., 2008 | Depression | 33 vs. 30 | 20-35 y | Stretching | 3 d/wk | 60 min | 12 weeks | / | Yes | Usual Care | EPDS |
| 14 | Da Costa et al., 2009 | Depression | 46 vs. 42 | 34.3 ± 3.4 y vs. 32.7 ± 4.8 y | AE | 3 d/wk | 30 min | 12 weeks | 60-85% HRmax | No | Usual Care | HRSD EPDS |
| 15 | Krogh et al., 2009 | Depression | 48 vs. 47 | 38.1 ± 9.0 y vs. 41.9 ± 8.7 y | AE | 2 d/wk | 90 min | 16 weeks | / | Yes | RE | HRSD BDI |
| 16 | Callaghan et al., 2011 | Depression | 19 vs. 19 | 57.0 ± 9.9 y vs. 50.4 ± 15.2 y | AE | 3 d/wk | 30 min | 4 weeks | / | Yes | Usual Care | BDI |
| 17 | de la Cerda et al., 2011 | Depression | 41 vs. 41 | 33.1 ± 5.4 y vs. 31.7 ± 6.8 y | AE | 3 d/wk | 45-60 min | 8 weeks | / | Yes | Usual Care | BDI |
| 18 | Lavretsky et al., 2011 | Depression | 33 vs. 35 | 69.1 ± 7 y vs. 72.0 ± 7.4 y | MBE (Tai Chi) | 1 d/wk | 120 min | 10 weeks | / | Yes | Health Education | HRSD |
| 19 | Mota-Pereira et al., 2011 | Depression | 19 vs. 10 | 45.33 ± 3.11 y vs.  48.68 ± 2.3 y | AE | 5 d/wk | 30-45 min | 12 weeks | / | Yes | Usual Care | HRSD BDI |
| 20 | Roshan et al., 2011 | Depression | 12 vs. 12 | 16.91 ± 1.03 y vs. 16.83 ± 0.82 y | AE (walking) | 3 d/wk | 60 min | 6 weeks | 60-79% HRmax | Yes | Usual Care | HRSD |
| 21 | Schuch et al., 2011 | Depression | 15 vs. 11 | 42.8 ± 12.4 y vs. 42.5 ± 13.5 y | AE | 3 d/wk | 45 min | 2 weeks | / | Yes | Usual Care | HRSD |
| 22 | Hemat-Far et al., 2012 | Depression | 10 vs. 10 | 18-25 y | AE (running) | 3 d/wk | 40-60 min | 8 weeks | 65-60% HRmax | Yes | Usual Care | BDI |
| 23 | Krogh et al., 2012 | Depression | 56 vs. 59 | 39.7 ± 11.3 y vs. 43.4 ± 11.2 y | AE | 3 d/wk | 45 min | 12 weeks | / | Yes | Stretching | HRSD BDI |
| 24 | Mitchell et al., 2012 | Depression | 12 vs. 12 | 18-37 y | MBE (Yoga) | 2 d/wk | 20 min | 12 weeks | / | Yes | Usual Care and Parenting Education | CES-D |
| 25 | Yeung et al., 2012 | Depression | 25 vs. 13 | 54 ± 12 y vs. 58 ± 7 y | MBE (Tai Chi) | 2 d/wk | 60 min | 12 weeks | / | Yes | Usual Care | HRSD |
| 26 | Field et al., 2013a | Depression | 37 vs. 38 | 24.4 ± 4.7 y vs. 26.0 ± 5.6 y | MBE (Tai Chi/ Yoga) | 1 d/week | 20 min | 12 weeks | / | Yes | Usual Care | CES-D |
| 27 | Field et al., 2013b | Depression | 40 vs. 39 | 24.4 ± 4.7 y vs. 24.5 ± 5.02 y | MBE (Yoga) | 1 d/wk | 20 min | 12 weeks | / | Yes | Usual Care and Social Support | EPDS CES-D |
| 28 | Gangadhar et al., 2013 | Depression | 27 vs. 16 | 33.7 ± 10.46 y vs. 31.4 ± 5.87 y | MBE (Yoga) | 7 d/wk | 60 min | 4 weeks | / | Yes | Usual Care | HRSD |
| 29 | Naveen et al., 2013 | Depression | 22 vs. 21 | 33.6 ± 10.3 y vs. 32.4 ± 7 y | MBE (Yoga) | 7 d/wk | 60 min | 12 weeks | / | Yes | Usual Care | HRSD |
| 30 | Ho et al., 2014 | Depression | 26 vs. 26 | 43.62 vs. 13.3 y vs. 48.81 ± 11.30 y | AE | 5 d/wk | 40 min | 3 weeks | / | Yes | Usual Care | BDI |
| 31 | Sarubin et al., 2014 | Depression | 22 vs. 31 | 37.27 ± 11.85 y vs. 42.36 ± 12.85 y | MBE (Yoga) | 1 d/wk | 60 min | 5 weeks | / | Yes | Usual Care | HRSD |
| 32 | Belvederi Murri et al., 2015 | Depression | 42 vs. 42 | 75.0 ± 6.2 y vs. 75.6 ± 5.6 y | AE (Cycling) | 3 d/wk | 60 min | 24 weeks | 60% HRmax | Yes | Usual Care | HRSD |
| 33 | Buttner et al., 2015 | Depression | 23 vs. 27 | 29.81 ± 5.17 y vs. 32.45 ± 4.78 y | MBE (Yoga) | 2 d/wk | 60 min | 8 weeks | / | Yes | Usual Care | HRSD |
| 34 | Carter et al., 2015 | Depression | 28 vs. 29 | 15.4 ± 1.0 y vs. 15.4 ± 0.9 y | AE | 2 d/wk | 60 min | 6 weeks | / | Yes | Usual Care | CDI-2 |
| 35 | Doose et al., 2015 | Depression | 23 vs. 12 | 46.07 ± 10.32 y vs. 51.25 ± 10.22 y | AE (Walking and Running) | 3 d/wk | 60 min | 8 weeks | / | Yes | Usual Care | HRSD BDI |
| 36 | Kerling et al., 2015 | Depression | 22 vs. 20 | 44.2 ± 8.5 y vs. 40.9 ± 11.9 y | AE (Cycling) | 3 d/wk | 45 min | 6 weeks | 50% of maximum workload | Yes | Usual Care | BDI |
| 37 | Majumder et al., 2015 | Depression | 22 vs. 21 | 30.5 ± 8.9 y vs. 29.6 ± 8.2 y | AE (Running) | 7 d/wk | 30 min | 24 weeks | 60-84% HRmax | Yes | Usual Care | HRSD |
| 38 | Schuch et al., 2015 | Depression | 25 vs. 25 | 35.36 ± 2.6 y vs. 33.28 ± 2.7 y | AE | 3 d/wk | 42 min | 2 weeks | 50-70% HRmax | Yes | Usual Care | HRSD |
| 39 | Legrand et al., 2016 | Depression | 14 vs. 11 | 45.3 ± 10.6 y vs. 41.8 ± 13.2 y | AE (walking/ jogging) | 7 d/wk | 30 min | 10 consecutive days | 65-75% HRmax | Yes | Stretching | BDI |
| 40 | Schuver et al., 2016 | Depression | 16 vs. 18 | 39.8 ± 11.23 y vs. 45.55 ± 12.30 y | AE (Walking) | 2 d/wk | 65 min | 12 weeks | / | Yes | MBE (Yoga) | BDI |
| 41 | Siqueira et al., 2016 | Depression | 20 vs. 20 | 39.76 ± 11.6 y vs. 37.86 ± 9.85 y | AE | 4 d/wk | 20-60 min | 4 weeks | 60-90% HRmax | / | Usual Care | HRSD BDI |
| 42 | Toni et al., 2016 | Depression | 38 vs. 50 | 75.0 ± 6.4 y vs. 73.5 ± 6.9 y | AE | 3 d/wk | 60 min | 24 weeks | / | Yes | Usual Care | HRSD |
| 43 | Forsyth et al., 2017 | Depression | 11 vs. 11 | 27 ± 5.5 y vs. 25 ± 5.1 y | AE | 3 d/wk | 50 min | 12 weeks | 40-65% HRmax | Yes | Usual Care | EPDS |
| 44 | Olson et al., 2017 | Depression | 15 vs. 15 | 21.0 ± 1.9 y vs. 21.2 ± 2.2 y | AE | 3 d/wk | 30-45 min | 8 weeks | 40-65% HRmax | Yes | Stretching | BDI |
| 45 | Prathikanti et al., 2017 | Depression | 15 vs. 10 | 43.1 ± 15.2 y vs. 43.8 ± 14.7 y | MBE (Yoga) | 2 d/wk | 90 min | 8 weeks | / | Yes | Usual Care | BDI |
| 46 | Turner et al., 2017 | Depression | 17 vs. 25 | 14-17 y | AE | 2 d/wk | 60 min | 6 weeks | / | Yes | Usual Care | CDI-2 |
| 47 | Uebelacker et al., 2017 | Depression | 63 vs. 59 | 46.78 ± 12.27 y vs. 46.2 ± 12.13 y | MBE (Yoga) | 2 d/wk | 80 min | 10 weeks | / | Yes | Healthy Living Workshop | QIDS |
| 48 | Yeung et al., 2017 | Depression | 18 vs. 19 | 53 ± 14 y vs. 55 ± 15 y | MBE (Tai Chi) | 2 d/wk | 60 min | 12 weeks | / | Yes | Usual Care | HRSD BDI |
| 49 | Cheung et al., 2018 | Depression | 17 vs. 17 | 47.4 ± 11.2 y vs. 48.1 ± 10.8 y | AE | 3 d/wk | 30-60 min | 12 weeks | 60% HRmax | Yes | Usual Care | HRSD |
| 50 | Gerber et al., 2018 | Depression | 25 vs. 25 | 36.4 ± 12.4 y vs. 36.5 ± 10.4 y | Others (Sprint Interval Training) | 3 d/wk | 35 min | 4 weeks | 80% VO2max | Yes | AE | BDI |
| 51 | Minghetti et al., 2018 | Depression | 29 vs. 30 | 35 ± 12 y vs. 37 ± 10 y | Others (Sprint Interval Training) | 3 d/wk | 35 min | 4 weeks | 60% of Maximal Power Output | Yes | AE | BDI |
| 52 | Tolahunase et al., 2018a | Depression | 89 vs. 89 | 38 ± 9 y vs. 40 ± 8 y | MBE (Yoga) | 5 d/wk | 120 min | 12 weeks | / | Yes | Usual Care | BDI |
| 53 | Bressington et al., 2019 | Depression | 23 vs. 27 | 46.30 ± 12.84 y vs. 49.37 ± 9.13 y | MBE (Yoga) | 2 d/wk | 45 min | 4 weeks | / | Yes | Usual Care | DASS |
| 54 | Kumar et al., 2019 | Depression | 40 vs. 40 | 36.90 ± 10.17 y vs. 39.48 ± 13.29 y | MBE (Yoga) | 5 d/wk | 45 min | 4 weeks | / | Yes | Usual Care | MADRS |
| 55 | Tasci et al., 2019 | Depression | 17 vs. 16 | 37.24 ± 7.93 y vs. 39.81 ± 8.35 y | AE (Walking) | 4 d/wk | 30 min | 12 weeks | / | No | Usual Care | HRSD |
| 56 | Zhang et al., 2019 | Depression | 22 vs. 20 | 31.4 ± 7.2 y vs. 32.2 ± 7.6 y | AE (Jogging) | 3 d/wk | 15 min | 8 weeks | 110 beats/min | No | Usual Care | HRSD |
| 57 | Chau et al., 2020 | Depression | 42 vs. 42 | 47.4 ±10.6 y | ME (AE + RE) | 3 d/wk | 60 min | 12 weeks | 50-70% HRmax | Yes | Usual Care | HRSD |
| 58 | Gerber et al., 2020 | Depression | 14 vs. 11 | 39.4 ± 9.7 y vs. 36.4 ± 14.8 y | AE (Cycling) | 3 d/wk | 40-50 min | 6 weeks | 60-75% HRmax | Yes | Stretching | BDI |
| 59 | Hyvonen et al., 2020 | Depression | 52 vs. 57 | 41.8 ± 9.96 y vs. 36.5 ± 10.03 y | MBE (Dance and Movement Therapy) | 2 d/wk | 75 min | 10 weeks | / | Yes | Usual Care | BDI |
| 60 | Imboden et al., 2020 | Depression | 22 vs. 20 | 41.3 ± 9.2 y vs. 38.3 ± 13.4 y | AE (Cycling) | 3 d/wk | 45 min | 6 weeks | 60-75% HRmax | Yes | Stretching | HRSD BDI |
| 61 | Ozkan et al., 2020 | Depression | 34 vs. 31 | 28.9 ± 4.83 y | AE | 5 d/wk | 30 min | 4 weeks | / | Yes | Usual Care | EPDS |
| 62 | Rao et al., 2020 | Depression | 40 vs. 40 | 40.0 ± 10.18 y vs. 43.63 ± 11.86 y | ME (AE + MBE [Dancing]) | 3 d/wk | 35 min | 8 weeks | / | Yes | Usual Care | BDI |
| 63 | Adagide et al., 2021 | Depression | 50 vs. 50 | 18-45 y | AE | 3 d/wk | 30-45 min | 14 weeks | 55-74 HRmax | Yes | Usual Care | BDI |
| 64 | Bieber et al., 2021 | Depression | 47 vs. 36 | 48.38 ± 10.21 y vs. 51.31 ± 9.19 y | MBE (Yoga) | 3 d/wk | 90 min | 12 weeks | / | Yes | Usual Care | BDI |
| 65 | Bruchle et al., 2021 | Depression | 23 vs. 18 | 33.3 ± 3.06 y vs. 40.11 ± 3.63 y | ME (coordination + endurance + RE) | 3 d/wk | 60 min | 3 weeks | / | Yes | Usual Care | HRSD BDI |
| 66 | Kang et al., 2021 | Depression | 25 vs. 25 | 48.9 ± 5.24 y vs. 49.9 ± 4.85 y | MBE (Yoga) | 2 d/wk | 75 min | 12 weeks | / | Yes | Usual Care | BDI |
| 67 | Lavretsky et al., 2021 | Depression | 62 vs. 63 | 69.2 ± 6.9 y vs. 69.4 ± 6.2 y | MBE (Tai Chi) | 7 d/wk | 20 min | 12 weeks | / | Yes | Health Education | HRSD |
| 68 | Lewis et al., 2021 | Depression | 132 vs. 106 | 31.03 ± 4.68 y vs. 31.44 ± 5.29 y | AE | 5 d/wk | 30 min | 24 weeks | 55-85% HRmax | Yes | Usual Care | EPDS |
| 69 | Ravindran et al., 2021 | Depression | 32 vs. 11 | 39.36 ± 11.69 y vs. 40.58 ± 12.72 y | MBE (Yoga) | 2 d/wk | 90 min | 8 weeks | / | Yes | Health Education | HRSD BDI |
| 70 | Srivastava et al., 2021 | Depression | 15 vs. 16 | 30.48 ± 10.22 y vs. 33.61 ± 8.97 y | MBE (Yoga) | 7 d/wk | 30 min | 8 weeks | / | Yes | Usual Care | HRSD |
| 71 | Broocks et al., 1998 | Anxiety Disorder | 16 vs. 15 | 31.8 ± 9.5 y vs. 33.9 ± 9.2 y | AE (Running) | 3-4 d/wk | 40 min | 10 weeks | / | No | Usual Care | HARS |
| 72 | Herring et al., 2011 | Anxiety Disorder | 10 vs. 10 | 18-37 y | AE | 2 d/wk | 16 min | 6 weeks | 60% HRmax | Yes | RE | STAI-Trait |
| 73 | Song et al., 2014 | Anxiety Disorder | 16 vs. 16 | 65.3 ± 7.1 y vs. 66.1 ± 8.3 y | MBE (Tai Chi) | 7 d/wk | 35 min | 6 weeks | / | Yes | Usual Care | HARS |
| 74 | Ma et al., 2017 | Anxiety Disorder | 41 vs. 42 | 39.76 ± 11.09 y vs. 40.45 ± 11.25 y | AE | 5 d/wk | 30 min | 12 weeks | / | No | Usual Care | STAI-Trait |
| 75 | Gordon et al., 2021 | Anxiety Disorder | 12 vs. 15 | 26.5 ± 5.8 y vs. 26.7 ± 4.9 y | RE | 2 d/wk | 25 min | 8 weeks | / | Yes | Usual Care | STAI-Trait |
| 76 | Carter et al., 2013 | PTSD | 14 vs. 11 | 58.5 ± 3.8 y vs. 58.4 ± 4.8 y | MBE (Yoga) | 7 d/wk | 30 min | 24 weeks | / | Yes | Usual Care | CAPS |
| 77 | Mitchell et al., 2014 | PTSD | 20 vs. 18 | 44.37 ± 12.37 | MBE (Yoga) | 1 d/wk | 75 min | 6 weeks | / | Yes | Usual Care | PCL |
| 78 | Thordardottir et al., 2014 | PTSD | 26 vs. 32 | 20-67 y | MBE (Yoga) | 2 d/wk | 60 min | 6 weeks | / | Yes | Usual Care | PDS5 |
| 79 | van der Kolk et al., 2014 | PTSD | 32 vs. 32 | 41.5 ± 12.2 y vs. 44.3 ± 11.9 y | MBE (Yoga) | 1 d/wk | 60 min | 10 weeks | / | Yes | Usual Care | CAPS |
| 80 | Jindani et al., 2015 | PTSD | 29 vs. 21 | 41 ± 9.51y | MBE (Yoga) | 7 d/wk | 28 min | 8 weeks | / | Yes | Usual Care | PCL |
| 81 | Quinones et al., 2015 | PTSD | 48 vs. 52 | / | MBE (Yoga) | 7 d/wk | 60 min | 16 weeks | / | Yes | Usual Care | PCL |
| 82 | Rosenbaum et al., 2015 | PTSD | 30 vs. 28 | 47.1 ± 11.3 y vs. 52.0 ± 12.7 y | ME (AE [walking] + RE) | 3 d/wk | 30 min | 12 weeks | / | Yes | Usual Care | PCL |
| 83 | Goldstein et al., 2018 | PTSD | 21 vs. 26 | 47.42 ± 15.94 y vs. 46.31 ± 14.37 y | ME (AE + RE + MBE [Yoga]) | 3 d/wk | 60 min | 12 weeks | / | Yes | Usual Care | CAPS |
| 84 | Whitworth et al., 2019 | PTSD | 11 vs. 11 | 33.8 ± 11.1 y vs. 32.1 ± 15.6 y | RE | 3 d/wk | 30 min | 3 weeks | / | Yes | Usual Care | PDS5 |
| 85 | Hall et al., 2020 | PTSD | 36 vs. 18 | 67.7 ± 3.2 y vs. 66.9 ± 4.3 y | ME (AE + RE + Others [Balance, Flexibility]) | 3 d/wk | 60-90 min | 12 weeks | / | Yes | Usual Care | PCL |
| 86 | Nguyen-Feng et al., 2020 | PTSD | 31 vs. 29 | 42.9 ± 12 y | MBE (Yoga) | 1 d/wk | 60 min | 10 weeks | / | Yes | Health Education | CAPS |
| 87 | Duraiswamy et al., 2007 | Schizophrenia | 21 vs. 20 | 32.53 ± 7.9 vs. 31.30 ± 7.9 | MBE (yoga) | 5 d/wk | 60 min | 3 weeks | / | Yes | AE | PANSS  - Positive Score - Negative Score |
| 88 | Acil et al., 2008 | Schizophrenia | 15 vs. 15 | 21-45 y | AE | 3 d/wk | 40 min | 10 weeks | / | Yes | Usual Care | SAPS SANS BSI |
| 89 | Behere et al., 2011 | Schizophrenia | 27 vs. 17 | 31.3 ± 9.3 y vs. 30.2 ± 8 y | MBE (Yoga) | 7 d/wk | 60 min | 12 weeks | / | Yes | AE | PANSS  - Positive Score - Negative Score |
| 90 | Ho et al., 2012 | Schizophrenia | 15 vs. 15 | 51.87 ± 10.85 y vs. 53.47 ± 8.63 y | MBE (Tai Chi) | 2 d/wk | 60 min | 6 weeks | / | Yes | Usual Care | SANS |
| 91 | Takahashi et al., 2012 | Schizophrenia | 13 vs. 10 | 43.5 ± 11.8 y vs. 39.9 ± 13.6 y | ME (AE + Stretching) | 6 d/wk | 60-120 min | 12 weeks | / | Yes | Usual Care | PANSS  - Positive Score - Negative Score - Total Score |
| 92 | Varambally et al., 2012 | Schizophrenia | 22 vs. 39 | 32.8 ± 10.0 y vs. 30.6 ± 7.3 y | AE | 6 d/wk | 45 min | 4 weeks | / | Yes | MBE (Yoga) | PANSS  - Positive Score - Negative Score - Total Score |
| 93 | Ikai et al., 2013 | Schizophrenia | 23 vs. 21 | 54.8 ± 9.0 y vs. 51.5 ± 15.1 y | MBE (Yoga) | 1 d/wk | 60 min | 8 weeks | / | Yes | Usual Care | PANSS  - Positive Score - Negative Score - Total Score |
| 94 | Jayaram et al., 2013 | Schizophrenia | 15 vs. 12 | 28.33 ± 4.7 y vs. 29.5 ± 8.2 y | MBE (Yoga) | 7 d/wk | 60 min | 4 weeks | / | Yes | Usual Care | SAPS SANS |
| 95 | Ikai et al., 2014 | Schizophrenia | 18 vs. 18 | 53.5 ± 9.9 y vs. 48.2 ± 12.3 y | MBE (Yoga) | 1 d/wk | 60 min | 8 weeks | / | Yes | Usual Care | PANSS  - Positive Score - Negative Score - Total Score |
| 96 | Kaltsatou et al., 2015 | Schizophrenia | 16 vs. 15 | 59.5 ± 19.6 y vs. 60.4 ± 8.6 y | ME (Stretching + Dance) | 3 d/wk | 60 min | 32 weeks | 60-70% HRmax | Yes | Usual Care | PANSS  - Positive Score - Negative Score - Total Score |
| 97 | Lee et al., 2015 | Schizophrenia | 18 vs. 20 | 41.5 ± 10.5 y vs. 41.8 ± 11.1 y | MBE (Dance and Movement Therapy) | 1 d/wk | 60 min | 12 weeks | / | Yes | Usual Care | PANSS  - Positive Score - Negative Score |
| 98 | Loh et al., 2015 | Schizophrenia | 48 vs. 52 | 46 ± 14 y  vs. 53 ± 11 y | AE (Walking) | 3 d/wk | 40 min | 12 weeks | / | Yes | Usual Care | PANSS  - Positive Score - Negative Score |
| 99 | Paikkatt et al., 2015 | Schizophrenia | 15 vs. 15 | 20-50 y | MBE (Yoga) | 7 d/wk | 90 min | 4 weeks | / | Yes | Usual Care | PANSS  - Positive Score - Negative Score |
| 100 | Silva et al., 2015 | Schizophrenia | 12 vs. 9 | 32.91 ± 2.28 y vs. 33.55 ± 2.63 y | RE | 3 d/wk | 60 min | 20 weeks | / | Yes | Usual Care | PANSS  - Positive Score - Negative Score - Total Score |
| 101 | Areshtanab et al., 2016 | Schizophrenia | 34 vs. 34 | 37.29 ± 7.68 y vs. 38.35 ± 6.64 y | AE | 3 d/wk | 24 min | 8 weeks | 65-80% HRmax | Yes | Usual Care | SAPS SANS |
| 102 | Ho Rainbow et al., 2016 | Schizophrenia | 51 vs. 51 vs. 49 | 55.0 ± 7.4 y vs. 52.4 ± 9.6 y | ME (AE+RE+Stretching) | 3 d/wk | 60 min | 12 weeks | / | Yes | MBE (Tai Chi) | PANSS  - Positive Score - Negative Score |
| 103 | Kang et al., 2016 | Schizophrenia | 118 vs. 126 | 45.9 ± 12.1 y | MBE (Tai Chi) | 2 d/mth | 120 min | 48 weeks | / | Yes | Usual Care | PANSS  - Positive Score - Negative Score - Total Score |
| 104 | Kavak et al., 2016 | Schizophrenia | 50 vs. 50 | 18-55 y | MBE (Yoga) | 5 d/wk | 40 min | 6 weeks | / | Yes | Usual Care | FROGS |
| 105 | Martin et al., 2016 | Schizophrenia | 30 vs. 19 | 41.05 ± 10.84 y vs. 37.52 ± 9.14 y | MBE (Dance and Movement Therapy) | 2 d/wk | 90 min | 10 weeks | / | Yes | Usual Care | SANS |
| 106 | Su et al., 2016 | Schizophrenia | 22 vs. 22 | 37.64 ± 8.23 y vs. 36.68 ± 8.33 y | AE | 3 d/wk | 40 min | 12 weeks | 55-69% HRmax | Yes | Stretching | PANSS  - Positive Score -Negative Score |
| 107 | Curcic et al., 2017 | Schizophrenia | 40 vs. 40 | 39.95 ± 9.51 y vs. 41.75 ± 9.45 y | AE (Walking or Jogging) | 4 d/wk | 45 min | 12 weeks | 65-75% HRmax | Yes | Usual Care | PANSS  - Positive Score - Negative Score - Total Score |
| 108 | Wang et al., 2018 | Schizophrenia | 25 vs. 23 | 38.3 ± 8.34 y vs. 38.72 ± 8.62 y | AE | 5 d/wk | 40 min | 12 weeks | / | Yes | Stretching | PANSS  - Positive Score - Negative Score - Total Score |
| 109 | Shimada et al., 2019 | Schizophrenia | 16 vs. 15 | 20-65 y | AE | 2 d/wk | 60 min | 12 weeks | 60-80 % of Aerobic Capacity | Yes | Usual Care | PANSS  - Positive Score - Negative Score - Total Score |
| 110 | Bryl et al., 2020 | Schizophrenia | 18 vs. 13 | 44.67 ± 10.97 y vs. 48.38 ± 12.70 y | MBE (Dance and Movement Therapy) | 2 d/wk | 60 min | 10 weeks | / | Yes | Usual Care | PANSS  - Negative Score |
| 111 | Shimada et al., 2020 | Schizophrenia | 20 vs. 20 | 50.14 ± 7.73 y vs. 49.75 ± 7.00 y | AE | 2 d/wk | 60 min | 12 weeks | 60-80% Aerobic Capacity | Yes | Usual Care | PANSS  - Positive Score - Negative Score - Total Score |
| 112 | Akbas et al., 2021 | Schizophrenia | 10 vs. 19 | 40.40 ± 8.39 y vs. 39.12 ± 6.68 y | MBE (Yoga) | 2 d/wk | 40-50 min | 6 weeks | / | Yes | Usual Care | BPRS |
| 113 | Gao et al., 2021 | Schizophrenia | 20 vs. 20 | 53.00 ± 8.13 y vs. 53.40 ± 7.04 y | MBE (Yijinjing) | 2 d/wk | 40 min | 12 weeks | / | Yes | Usual Care | PANSS  - Positive Score - Negative Score - Total Score |
| 114 | Govindaraj et al., 2021 | Schizophrenia | 26 vs. 25 | 33.62 ± 7.22 y vs. 32.92 ± 6.40 y | MBE (Yoga) | 4-5 d/wk | 60 min | 6 weeks | / | Yes | Usual Care | SAPS SANS |
| 115 | Lo et al., 2021 | Schizophrenia | 17 vs. 16 | 33.45 ± 10.30 y vs. 31.83 ± 10.46 y | Others (HIIE) | 3 d/wk | 15 min | 12 weeks | 105% Functional Threshold Power | Yes | AE | PANSS  - Positive Score - Negative Score - Total Score |
| 116 | Rao et al., 2021 | Schizophrenia | 45 vs. 44 | 34.79 ± 7.06 y vs. 33.65 ± 8.95 y | MBE (Yoga) | 6 d/wk | 60 min | 12 weeks | / | Yes | Usual Care | SAPS SANS |
| 117 | Senormanci et al., 2021 | Schizophrenia | 20 vs. 19 | 40.5 ± 9.10 y vs. 43 ± 6.77 y | RE | 2 d/wk | 60 min | 12 weeks | / | Yes | Usual Care | SAPS SANS |

(**Notes**. AE: aerobic exercise; RE: resistant exercise; MBE: mind-body exercise; ME: multimodal exercise; RM: repetition maximum; BDI: Beck Depression Inventory; HRSD: Hamilton Rating Scale for Depression; EPDS: Edinburgh Postnatal Depression Score; BRMS: Bech-Rafaelsen Melancholy Scale; CES-D: Center for Epidemiologic Studies - Depression scale; CDI-2: Children's Depression Inventory – 2; DASS: Depression Anxiety Stress Scale; MADRS: Montgomery-Asberg Depression Rating Scale; QIDS: Quick Inventory of Depression Symptomatology; STAI-Trait: Trait Scale of the State-Trait Anxiety Inventory; HARS: Hamilton Anxiety Rating Scale; PCL: Posttraumatic Stress Disorder Checklist; CAPS: Clinician-Administered Posttraumatic Stress Disorder Scale; PDS5: Posttraumatic Diagnostic Scale for Diagnostic and Statistical Manual of Mental Disorders – 5; PANSS: Positive and Negative Syndrome Scale for Schizophrenia; SAPS: Scale for the Assessment of Positive Symptoms; SANS: Scale for the Assessment of Negative Symptoms; BSI: Brief Symptom Inventory; FROGS: Functional Remission of General Schizophrenia Scale; BPRS: Brief Psychiatric Rating Scale.)
