# Supplementary material for: Comparative Effectiveness of Multiple Exercise Interventions in the Treatment of Mental Health Disorders: A Systematic Review and Network Meta-Analysis
Source: Sports Med Open. 2022 Oct 29;8:135. doi: 10.1186/s40798-022-00529-5 (PMC9617247; doi:10.1186/s40798-022-00529-5)
Supplement: Supplementary file 7 — Additional file 7: Appendix 6. Risk of Bias Assessment. [file 40798_2022_529_MOESM7_ESM.docx]

**Appendix 6. Risk of Bias Assessment**

| **No** | **Study** | **Bias from Randomization Process** | **Bias due to Deviations from Intended Interventions** | **Bias Due to Missing Outcome Data** | **Bias in Measurement of Outcome** | **Bias in Selection of Reported Result** |
| --- | --- | --- | --- | --- | --- | --- |
| 1 | Doyne et al., 1987 | Some Concerns | High Risk | Low Risk | Some Concerns | Low Risk |
| 2 | McNeil et al., 1991 | Some Concerns | High Risk | Low Risk | Some Concerns | Low Risk |
| 3 | Veale et al., 1992 | Some Concerns | High Risk | Low Risk | Some Concerns | Low Risk |
| 4 | Singh et al., 1997 | Some Concerns | High Risk | Some Concerns | Some Concerns | Low Risk |
| 5 | Blumenthal et al., 1999 | Some Concerns | High Risk | Low Risk | Some Concerns | Low Risk |
| 6 | Armstrong et al., 2003 | Low Risk | High Risk | Low Risk | Some Concerns | Low Risk |
| 7 | Sharma et al., 2005 | Some Concerns | High Risk | Low Risk | Low Risk | Low Risk |
| 8 | Singh et al., 2005 | Low Risk | High Risk | Low Risk | Low Risk | Low Risk |
| 9 | Blumenthal et al., 2007 | Low Risk | High Risk | Low Risk | Low Risk | Low Risk |
| 10 | Brenes et al., 2007 | Some Concerns | High Risk | High Risk | Low Risk | Low Risk |
| 11 | Knubben et al., 2007 | Low Risk | High Risk | Low Risk | Low Risk | Low Risk |
| 12 | Daley et al., 2008 | Some Concerns | High Risk | High Risk | High Risk | Low Risk |
| 13 | Heh et al., 2008 | Some Concerns | High Risk | Some Concerns | Low Risk | Low Risk |
| 14 | Da Costa et al., 2009 | Low Risk | High Risk | Low Risk | Some Concerns | Low Risk |
| 15 | Krogh et al., 2009 | Low Risk | High Risk | Low Risk | Low Risk | Low Risk |
| 16 | Callaghan et al., 2011 | Low Risk | High Risk | Low Risk | Some Concerns | Low Risk |
| 17 | de la Cerda et al., 2011 | High Risk | High Risk | Low Risk | Some Concerns | Low Risk |
| 18 | Lavretsky et al., 2011 | Low Risk | High Risk | Low Risk | Low Risk | Low Risk |
| 19 | Mota-Pereira et al., 2011 | Some Concerns | High Risk | Some Concerns | Low Risk | Low Risk |
| 20 | Roshan et al., 2011 | Some Concerns | High Risk | Low Risk | Low Risk | Low Risk |
| 21 | Schuch et al., 2011 | Some Concerns | High Risk | Low Risk | Some Concerns | Low Risk |
| 22 | Hemat-Far et al., 2012 | Some Concerns | High Risk | Low Risk | Some Concerns | Low Risk |
| 23 | Krogh et al., 2012 | Low Risk | High Risk | Some Concerns | Low Risk | Low Risk |
| 24 | Mitchell et al., 2012 | Some Concerns | High Risk | Low Risk | Some Concerns | Low Risk |
| 25 | Yeung et al., 2012 | Low Risk | High Risk | Some Concerns | Some Concerns | Low Risk |
| 26 | Field et al., 2013a | Some Concerns | High Risk | Some Concerns | Some Concerns | Low Risk |
| 27 | Field et al., 2013b | Some Concerns | High Risk | Some Concerns | Some Concerns | Low Risk |
| 28 | Gangadhar et al., 2013 | High Risk | High Risk | High Risk | Low Risk | Low Risk |
| 29 | Naveen et al., 2013 | High Risk | High Risk | High Risk | Low Risk | Low Risk |
| 30 | Ho et al., 2014 | Low Risk | High Risk | Low Risk | Low Risk | Low Risk |
| 31 | Sarubin et al., 2014 | Some Concerns | High Risk | Low Risk | Low Risk | Low Risk |
| 32 | Belvederi Murri et al., 2015 | Low Risk | High Risk | Low Risk | Low Risk | Low Risk |
| 33 | Buttner et al., 2015 | Low Risk | High Risk | Low Risk | Low Risk | Low Risk |
| 34 | Carter et al., 2015 | Low Risk | High Risk | Low Risk | Low Risk | Low Risk |
| 35 | Doose et al., 2015 | Some Concerns | High Risk | Some Concerns | Some Concerns | Low Risk |
| 36 | Kerling et al., 2015 | Some Concerns | High Risk | Low Risk | Some Concerns | Low Risk |
| 37 | Majumder et al., 2015 | Some Concerns | High Risk | High Risk | Some Concerns | Low Risk |
| 38 | Schuch et al., 2015 | Low Risk | High Risk | Some Concerns | Some Concerns | Low Risk |
| 39 | Legrand et al., 2016 | Some Concerns | High Risk | Low Risk | Some Concerns | Low Risk |
| 40 | Schuver et al., 2016 | Low Risk | High Risk | Low Risk | Some Concerns | Low Risk |
| 41 | Siqueira et al., 2016 | Low Risk | High Risk | Some Concerns | Low Risk | Low Risk |
| 42 | Toni et al., 2016 | Some Concerns | High Risk | High Risk | Low Risk | Low Risk |
| 43 | Forsyth et al., 2017 | Some Concerns | High Risk | Low Risk | Some Concerns | Low Risk |
| 44 | Olson et al., 2017 | Some Concerns | High Risk | Low Risk | Some Concerns | Low Risk |
| 45 | Prathikanti et al., 2017 | Low Risk | High Risk | Low Risk | Some Concerns | Low Risk |
| 46 | Turner et al., 2017 | Low Risk | High Risk | Some Concerns | Some Concerns | Low Risk |
| 47 | Uebelacker et al., 2017 | Low Risk | High Risk | Some Concerns | Some Concerns | Low Risk |
| 48 | Yeung et al., 2017 | Low Risk | High Risk | Some Concerns | Some Concerns | Low Risk |
| 49 | Cheung et al., 2018 | Low Risk | High Risk | Low Risk | Some Concerns | Low Risk |
| 50 | Gerber et al., 2018 | Some Concerns | High Risk | Low Risk | Some Concerns | Low Risk |
| 51 | Minghetti et al., 2018 | Some Concerns | High Risk | Low Risk | Some Concerns | Low Risk |
| 52 | Tolahunase et al., 2018a | Low Risk | High Risk | Some Concerns | Low Risk | Low Risk |
| 53 | Bressington et al., 2019 | Low Risk | High Risk | Low Risk | Some Concerns | Low Risk |
| 54 | Kumar et al., 2019 | Low Risk | High Risk | Low Risk | High Risk | Low Risk |
| 55 | Tasci et al., 2019 | Some Concerns | High Risk | Low Risk | Low Risk | Low Risk |
| 56 | Zhang et al., 2019 | Some Concerns | High Risk | Low Risk | Low Risk | Low Risk |
| 57 | Chau et al., 2020 | Some Concerns | High Risk | Low Risk | Some Concerns | Low Risk |
| 58 | Gerber et al., 2020 | Low Risk | High Risk | Some Concerns | Low Risk | Low Risk |
| 59 | Hyvonen et al., 2020 | Some Concerns | High Risk | Low Risk | Some Concerns | Low Risk |
| 60 | Imboden et al., 2020 | Low Risk | High Risk | Low Risk | Some Concerns | Low Risk |
| 61 | Ozkan et al., 2020 | Some Concerns | High Risk | Some Concerns | High Risk | Low Risk |
| 62 | Rao et al., 2020 | High Risk | High Risk | Low Risk | High Risk | Low Risk |
| 63 | Adagide et al., 2021 | Some Concerns | High Risk | Some Concerns | Some Concerns | Low Risk |
| 64 | Bieber et al., 2021 | Low Risk | High Risk | Low Risk | Some Concerns | Low Risk |
| 65 | Bruchle et al., 2021 | High Risk | High Risk | Some Concerns | Low Risk | Low Risk |
| 66 | Kang et al., 2021 | Some Concerns | High Risk | High Risk | Some Concerns | Low Risk |
| 67 | Lavretsky et al., 2021 | Some Concerns | High Risk | Low Risk | Low Risk | Low Risk |
| 68 | Lewis et al., 2021 | Some Concerns | High Risk | High Risk | Some Concerns | Low Risk |
| 69 | Ravindran et al., 2021 | Low Risk | High Risk | Some Concerns | Low Risk | Low Risk |
| 70 | Srivastava et al., 2021 | High Risk | High Risk | Low Risk | Low Risk | Low Risk |
| 71 | Broocks et al., 1998 | Some Concerns | High Risk | High Risk | Some Concerns | Low Risk |
| 72 | Herring et al., 2011 | Some Concerns | High Risk | Low Risk | Some Concerns | Low Risk |
| 73 | Song et al., 2014 | Some Concerns | High Risk | Low Risk | Low Risk | Low Risk |
| 74 | Ma et al., 2017 | Low Risk | High Risk | Low Risk | Some Concerns | Low Risk |
| 75 | Gordon et al., 2021 | Some Concerns | High Risk | Low Risk | Some Concerns | Low Risk |
| 76 | Carter et al., 2013 | Some Concerns | High Risk | Some Concerns | Some Concerns | Low Risk |
| 77 | Mitchell et al., 2014 | Some Concerns | High Risk | Low Risk | Some Concerns | Low Risk |
| 78 | Thordardottir et al., 2014 | High Risk | High Risk | Some Concerns | Some Concerns | Low Risk |
| 79 | van der Kolk et al., 2014 | Some Concerns | High Risk | Low Risk | Some Concerns | Low Risk |
| 80 | Jindani et al., 2015 | Some Concerns | High Risk | Low Risk | Some Concerns | Low Risk |
| 81 | Quinones et al., 2015 | Some Concerns | High Risk | Low Risk | Some Concerns | Low Risk |
| 82 | Rosenbaum et al., 2015 | Low Risk | High Risk | Some Concerns | Some Concerns | Low Risk |
| 83 | Goldstein et al., 2018 | Some Concerns | High Risk | Low Risk | Some Concerns | Low Risk |
| 84 | Whitworth et al., 2019 | Low Risk | High Risk | Low Risk | Some Concerns | Low Risk |
| 85 | Hall et al., 2020 | Low Risk | High Risk | Low Risk | Some Concerns | Low Risk |
| 86 | Nguyen-Feng et al., 2020 | Some Concerns | High Risk | Low Risk | Some Concerns | Low Risk |
| 87 | Duraiswamy et al., 2007 | Some Concerns | High Risk | High Risk | Low Risk | Low Risk |
| 88 | Acil et al., 2008 | Some Concerns | High Risk | Low Risk | Some Concerns | Low Risk |
| 89 | Behere et al., 2011 | High Risk | High Risk | Some Concerns | Some Concerns | Low Risk |
| 90 | Ho et al., 2012 | Some Concerns | High Risk | Low Risk | Low Risk | Low Risk |
| 91 | Takahashi et al., 2012 | High Risk | High Risk | Low Risk | Low Risk | Low Risk |
| 92 | Varambally et al., 2012 | Low Risk | High Risk | Some Concerns | Low Risk | Low Risk |
| 93 | Ikai et al., 2013 | Low Risk | High Risk | Low Risk | Low Risk | Low Risk |
| 94 | Jayaram et al., 2013 | Some Concerns | High Risk | Low Risk | Low Risk | Low Risk |
| 95 | Ikai et al., 2014 | Low Risk | High Risk | Some Concerns | Low Risk | Low Risk |
| 96 | Kaltsatou et al., 2015 | Low Risk | High Risk | Low Risk | Low Risk | Low Risk |
| 97 | Lee et al., 2015 | Some Concerns | High Risk | Low Risk | Some Concerns | Low Risk |
| 98 | Loh et al., 2015 | Some Concerns | High Risk | Low Risk | Some Concerns | Low Risk |
| 99 | Paikkatt et al., 2015 | Some Concerns | High Risk | Some Concerns | Some Concerns | Low Risk |
| 100 | Silva et al., 2015 | Some Concerns | High Risk | Some Concerns | Low Risk | Low Risk |
| 101 | Areshtanab et al., 2016 | Some Concerns | High Risk | Low Risk | Low Risk | Low Risk |
| 102 | Ho Rainbow et al., 2016 | Some Concerns | High Risk | Low Risk | Some Concerns | Low Risk |
| 103 | Kang et al., 2016 | Low Risk | High Risk | Low Risk | Low Risk | Low Risk |
| 104 | Kavak et al., 2016 | High Risk | High Risk | Low Risk | Some Concerns | Low Risk |
| 105 | Martin et al., 2016 | Some Concerns | High Risk | Some Concerns | Low Risk | Low Risk |
| 106 | Su et al., 2016 | Some Concerns | High Risk | Low Risk | Low Risk | Low Risk |
| 107 | Curcic et al., 2017 | Some Concerns | High Risk | Low Risk | Low Risk | Low Risk |
| 108 | Wang et al., 2018 | Some Concerns | High Risk | Low Risk | Low Risk | Low Risk |
| 109 | Shimada et al., 2019 | Some Concerns | High Risk | Low Risk | Low Risk | Low Risk |
| 110 | Bryl et al., 2020 | Low Risk | High Risk | Low Risk | Some Concerns | Low Risk |
| 111 | Shimada et al., 2020 | Some Concerns | High Risk | Low Risk | Low Risk | Low Risk |
| 112 | Akbas et al., 2021 | High Risk | High Risk | Low Risk | Some Concerns | Low Risk |
| 113 | Gao et al., 2021 | Some Concerns | High Risk | Low Risk | Some Concerns | Low Risk |
| 114 | Govindaraj et al., 2021 | Low Risk | High Risk | Low Risk | Low Risk | Low Risk |
| 115 | Lo et al., 2021 | Some Concerns | High Risk | Low Risk | Some Concerns | Low Risk |
| 116 | Rao et al., 2021 | Some Concerns | High Risk | Low Risk | Some Concerns | Low Risk |
| 117 | Senormanci et al., 2021 | Some Concerns | High Risk | Low Risk | Low Risk | Low Risk |
