# Supplementary material for: Comparative Effectiveness of Multiple Exercise Interventions in the Treatment of Mental Health Disorders: A Systematic Review and Network Meta-Analysis
Source: Sports Med Open. 2022 Oct 29;8:135. doi: 10.1186/s40798-022-00529-5 (PMC9617247; doi:10.1186/s40798-022-00529-5)
Supplement: Supplementary file 8 — Additional file 8: Appendix 7. Grading of Recommendations, Assessment, Development and Evaluations (GRADE). [file 40798_2022_529_MOESM8_ESM.docx]

**Appendix 7. Grading of Recommendations, Assessment, Development and Evaluations (GRADE)**

| **Mental Health Disorders in General** | | | | | | |
| --- | --- | --- | --- | --- | --- | --- |
| **Comparison** | **Direct Evidence** | | **Indirect Evidence** | | **Network Meta-Analysis** | |
|  | **SMD (95% CrI)** | **Certainty of Evidence** | **SMD (95% CrI)** | **Certainty of Evidence** | **SMD (95% CrI)** | **Certainty of Evidence** |
| AE vs. Control | 3.17 (1.71, 4.65) | ⊕⊕OO *† | 3.14 (-1.14, 7.23) | ⊕OOO *†§ | 3.17 (1.77, 4.56) | ⊕⊕OO *† |
| AE vs. MBE | -1.67 (-8.38, 4.69) | ⊕OOO *†§ | -1.54 (-3.62, 0.60) | ⊕OOO *†§ | -1.54 (-3.51, 0.41) | ⊕OOO *†§ |
| AE vs. RE | -1.59 (-6.61, 3.41) | ⊕OOO *†§ | -2.19 (-6.46, 2.22) | ⊕OOO *†§ | -1.90 (-5.08, 1.39) | ⊕OOO *†§ |
| AE vs. Stretching | 4.50 (0.87, 8.16) | ⊕⊕OO *† | 5.65 (-2.81, 14.02) | ⊕OOO *†§ | 4.68 (1.25, 8.03) | ⊕⊕OO *† |
| Control vs. MBE | -4.71 (-6.24, -3.18) | ⊕⊕OO *† | -4.82 (-11.48, 1.82) | ⊕OOO *†§ | -4.72 (-6.22, -3.21) | ⊕⊕OO *† |
| Control vs. RE | -5.25 (-9.38, -1.10) | ⊕⊕OO *† | -4.75 (-10.03, 0.56) | ⊕OOO *†§ | -5.07 (-8.25, -1.90) | ⊕⊕OO *† |
| Control vs. Stretching | 2.62 (-5.96, 11.10) | ⊕OOO *†§ | 1.26 (-2.64, 5.25) | ⊕OOO *†§ | 1.53 (-2.07, 5.03) | ⊕OOO *†§ |
| **Depression** | | | | | | |
| **Comparison** | **Direct Evidence** | | **Indirect Evidence** | | **Network Meta-Analysis** | |
|  | **SMD (95% CrI)** | **Certainty of Evidence** | **SMD (95% CrI)** | **Certainty of Evidence** | **SMD (95% CrI)** | **Certainty of Evidence** |
| AE vs. Control | 3.09 (1.58, 4.65) | ⊕⊕OO *† | 3.65 (-1.33, 8.67) | ⊕OOO *†§ | 3.14 (1.69, 4.59) | ⊕⊕OO *† |
| AE vs. MBE | 2.60 (-6.30, 11.41) | ⊕⊕OO *§ | -0.70 (-3.01, 1.70) | ⊕⊕OO *§ | -0.43 (-2.67, 1.80) | ⊕⊕OO *§ |
| AE vs. RE | -1.95 (-7.86, 3.76) | ⊕OOO *§# | -2.43 (-8.44, 3.64) | ⊕⊕OO *§ | -2.26 (-6.35, 1.84) | ⊕OOO *§# |
| AE vs. Stretching | 3.57 (-0.17, 7.35) | ⊕⊕OO *§ | 5.83 (-2.64, 14.16) | ⊕⊕OO *§ | 3.92 (0.53, 7.34) | ⊕⊕⊕O * |
| Control vs. MBE | -3.72 (-5.53, -1.95) | ⊕⊕OO *† | -0.37 (-9.31, 9.05) | ⊕OOO *†§ | -3.58 (-5.34, -1.82) | ⊕⊕OO *† |
| Control vs. RE | -5.55 (-11.35, 0.09) | ⊕OOO *†§ | -5.22 (-11.08, 0.75) | ⊕OOO *†§ | -5.40 (-9.45, -1.32) | ⊕OOO *†# |
| Control vs. Stretching | 2.50 (-5.79, 10.92) | ⊕⊕OO *§ | 0.29 (-3.76, 4.47) | ⊕⊕OO *§ | 0.78 (-2.80, 4.46) | ⊕⊕OO *§ |
| **Anxiety Disorder** | | | | | | |
| **Comparison** | **Direct Evidence** | | **Indirect Evidence** | | **Network Meta-Analysis** | |
|  | **SMD (95% CrI)** | **Certainty of Evidence** | **SMD (95% CrI)** | **Certainty of Evidence** | **SMD (95% CrI)** | **Certainty of Evidence** |
| AE vs. Control | 1.53 (-5.42, 7.66) | ⊕⊕OO *§ | 6.72 (-6.13, 19.69) | ⊕OOO *‡§ | 2.33 (-3.58, 7.76) | ⊕OOO *‡§ |
| AE vs. RE | -0.29 (-9.74, 9.17) | ⊕⊕OO *§ | -5.90 (-17.13, 4.65) | ⊕OOO *‡§ | -2.82 (-9.93, 4.22) | ⊕OOO *‡§ |
| Control vs. RE | -7.20 (-16.51, 2.06) | ⊕⊕OO *§ | -1.87 (-13.17, 10.23) | ⊕OOO *‡§ | -5.17 (-11.88, 2.00) | ⊕OOO *‡§ |
| **Post-Traumatic Stress Disorder** | | | | | | |
| **Comparison** | **Direct Evidence** | | **Indirect Evidence** | | **Network Meta-Analysis** | |
|  | **SMD (95% CrI)** | **Certainty of Evidence** | **SMD (95% CrI)** | **Certainty of Evidence** | **SMD (95% CrI)** | **Certainty of Evidence** |
| Control vs. MBE | 9.07 (2.60, 15.82) | ⊕OOO *† | / | / | 9.07 (2.60, 15.82) | ⊕OOO *‡‡ |
| Control vs. ME | 7.84 (-1.59, 18.02) | ⊕OOO *†§ | / | / | 7.84 (-1.59, 18.02) | ⊕OOO *‡§ |
| Control vs. RE | -0.08 (-17.96, 18.15) | ⊕OOO *†§ | / | / | -0.08 (-17.96, 18.15) | ⊕OOO *‡§ |
| **Overall Symptom of Schizophrenia** | | | | | | |
| **Comparison** | **Direct Evidence** | | **Indirect Evidence** | | **Network Meta-Analysis** | |
|  | **SMD (95% CrI)** | **Certainty of Evidence** | **SMD (95% CrI)** | **Certainty of Evidence** | **SMD (95% CrI)** | **Certainty of Evidence** |
| AE vs. Control | 6.08 (0.42, 12.61) | ⊕⊕⊕O * | -0.44 (-13.10, 12.39) | ⊕⊕OO *§ | 4.90 (0.07, 10.35) | ⊕⊕OO *§ |
| AE vs. MBE | -6.16 (-17.90, 5.52) | ⊕⊕OO *§ | 0.36 (-6.99, 8.31) | ⊕⊕OO *§ | -1.62 (-7.62, 4.93) | ⊕⊕OO *§ |
| Control vs. MBE | -5.68 (-10.45, -0.85) | ⊕⊕OO *† | -12.30 (-25.71, 0.14) | ⊕OOO *†§ | -6.55 (-11.10, -2.08) | ⊕⊕OO *† |
| **Positive Symptom of Schizophrenia** | | | | | | |
| **Comparison** | **Direct Evidence** | | **Indirect Evidence** | | **Network Meta-Analysis** | |
|  | **SMD (95% CrI)** | **Certainty of Evidence** | **SMD (95% CrI)** | **Certainty of Evidence** | **SMD (95% CrI)** | **Certainty of Evidence** |
| AE vs. Control | 2.41 (0.29, 4.70) | ⊕⊕⊕O * | 0.59 (-2.99, 4.24) | ⊕⊕OO *§ | 1.93 (0.10, 3.88) | ⊕⊕⊕O * |
| AE vs. MBE | -1.93 (-5.14, 1.31) | ⊕⊕OO *§ | -0.05 (-2.78, 2.74) | ⊕⊕OO *§ | -0.82 (-2.93, 1.30) | ⊕⊕OO *§ |
| Control vs. MBE | -2.47 (-4.32, -0.83) | ⊕⊕OO *† | -4.33 (-8.34, -0.55) | ⊕⊕OO *† | -2.75 (-4.41, -1.25) | ⊕⊕OO *† |
| **Negative Symptom of Schizophrenia** | | | | | | |
| **Comparison** | **Direct Evidence** | | **Indirect Evidence** | | **Network Meta-Analysis** | |
|  | **SMD (95% CrI)** | **Certainty of Evidence** | **SMD (95% CrI)** | **Certainty of Evidence** | **SMD (95% CrI)** | **Certainty of Evidence** |
| AE vs. Control | 3.20 (-1.95, 8.35) | ⊕OOO *†§ | 3.32 (-4.11, 10.80) | ⊕OOO *†§ | 3.28 (-0.81, 7.46) | ⊕OOO *†§ |
| AE vs. MBE | -2.34 (-8.84, 4.28) | ⊕⊕OO *§ | -2.32 (-8.53, 3.78) | ⊕⊕OO *§ | -2.26 (-6.65, 2.15) | ⊕⊕OO *§ |
| Control vs. MBE | -6.32 (-9.66, -3.06) | ⊕⊕OO *† | -2.40 (-9.58, 4.59) | ⊕OOO *†§ | -5.55 (-8.51, -2.65) | ⊕⊕OO *† |
| Control vs. ME | -1.67 (-9.44, 5.95) | ⊕OOO *§# | -14.54 (-25.23, -3.83) | ⊕⊕⊕O * | -6.07 (-12.88, 0.47) | ⊕⊕OO *# |
| MBE vs. ME | -8.38 (-18.60, 1.98) | ⊕⊕OO *§ | 4.44 (-3.47, 12.42) | ⊕⊕OO *§ | -0.52 (-7.45, 6.33) | ⊕⊕OO *§ |

(Notes. 95% CrI: 95% credibility interval.⊕⊕⊕O: moderate certainty;⊕⊕OO: low certainty;⊕OOO: very low certainty. *: down rating for risk of bias; †: down rating for inconsistency; ‡: down rating for indirectness; §: down rating for imprecision; #: down rating for publication bias.)
