# Supplementary material for: Comparative Effectiveness of Multiple Exercise Interventions in the Treatment of Mental Health Disorders: A Systematic Review and Network Meta-Analysis
Source: Sports Med Open. 2022 Oct 29;8:135. doi: 10.1186/s40798-022-00529-5 (PMC9617247; doi:10.1186/s40798-022-00529-5)
Supplement: Supplementary file 9 — Additional file 9: Appendix 8. Outcomes of Pairwise Meta-Analysis, Network Meta-Analysis and Meta Regression. [file 40798_2022_529_MOESM9_ESM.docx]

**Appendix 8. Outcomes of Pairwise Meta-Analysis, Network Meta-Analysis and Meta Regression**

**8.1 Pairwise Meta-Analysis, Network Meta-Analysis and Meta Regression for Mental Health Disorders in General**

**8.1.1 Pairwise Meta-Analysis for Mental Health Disorders in General**

**[1] AE vs. Control (n =36)**


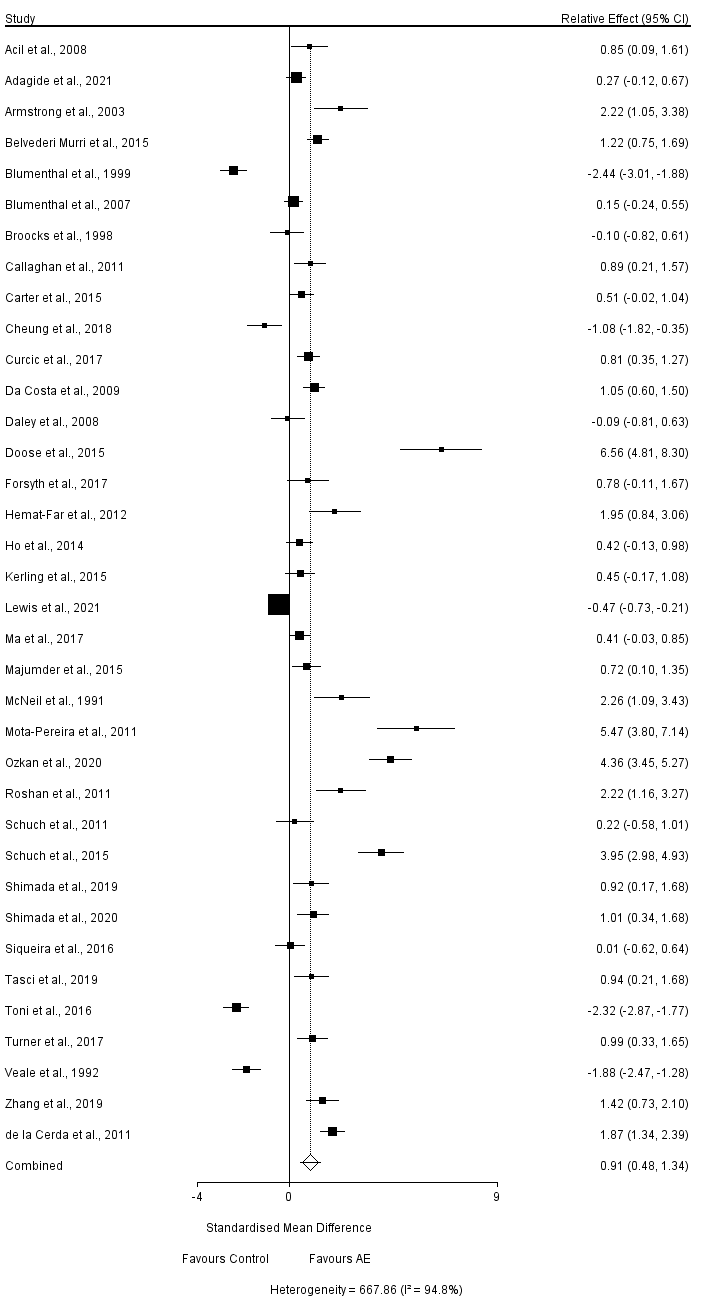


**[2] MBE vs. Control (n = 36)**


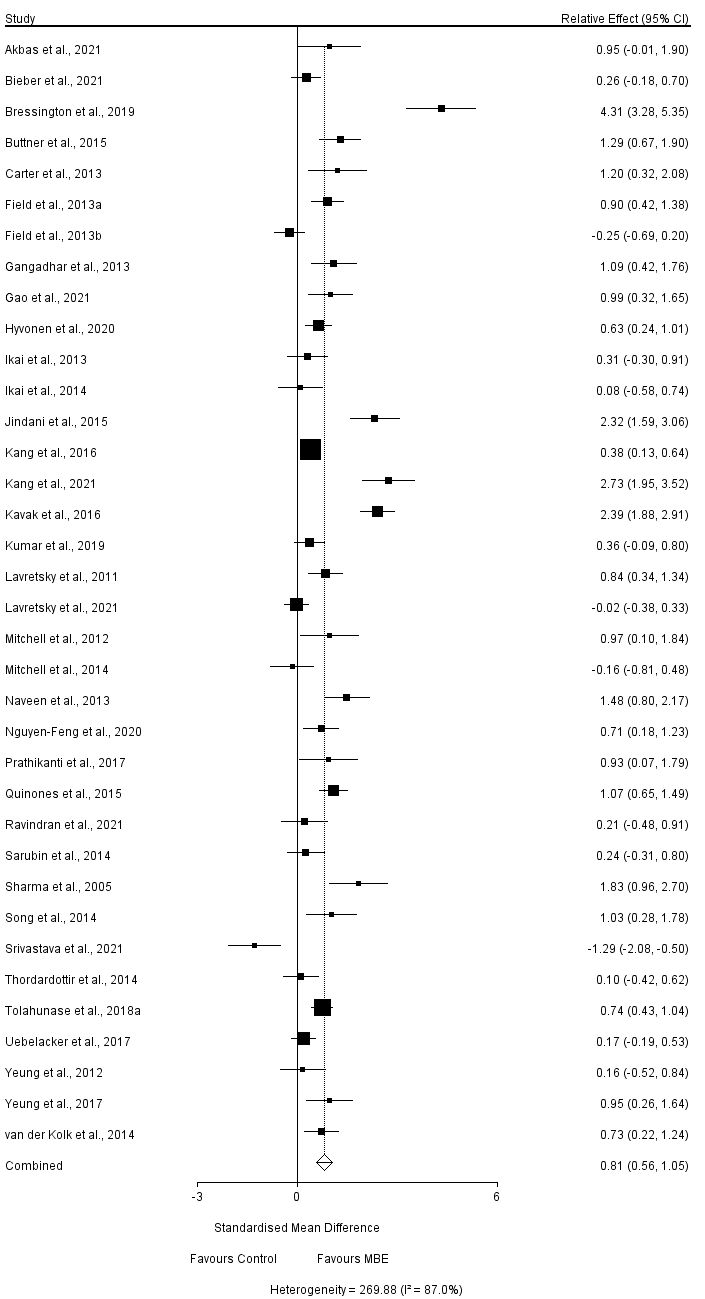


**[3] RE vs. Control (n = 5)**


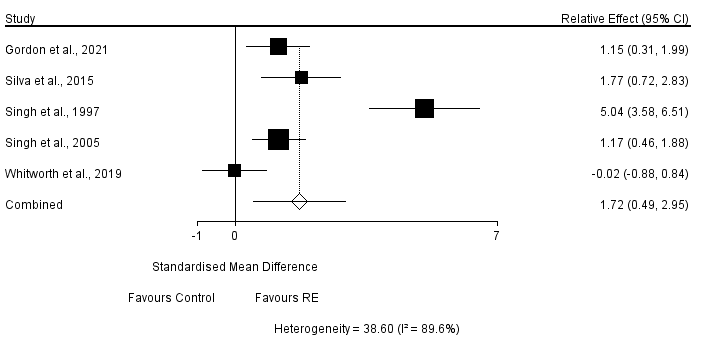


**[4] ME vs. Control (n = 9)**


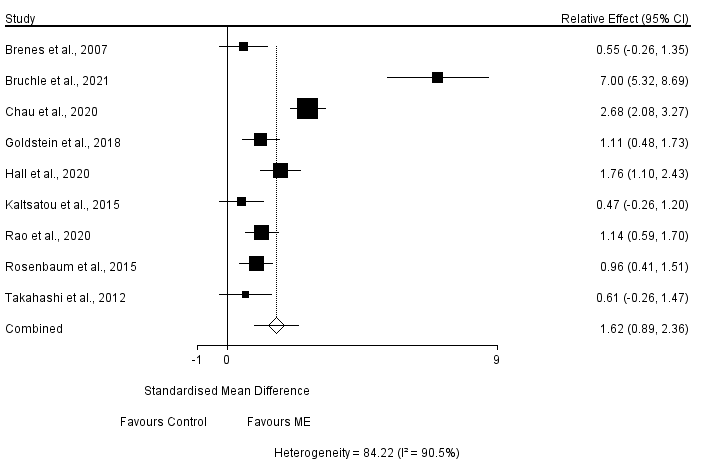


**[5] AE vs. RE (n = 3)**


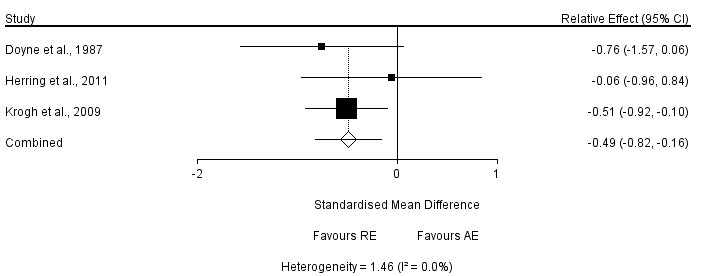


**[6] AE vs. MBE (n = 2)**


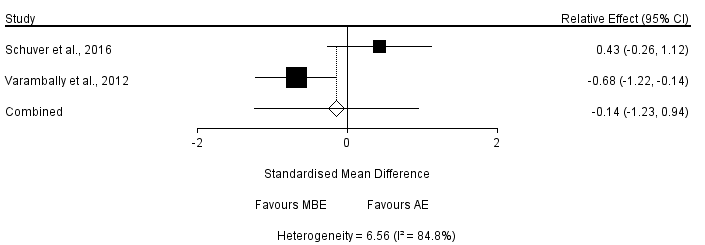


**[7] AE vs. Stretching (n = 7)**


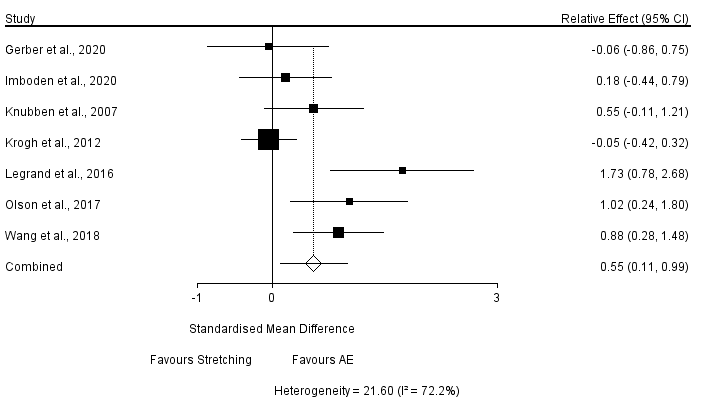


**[8] AE vs. Others (n = 3)**


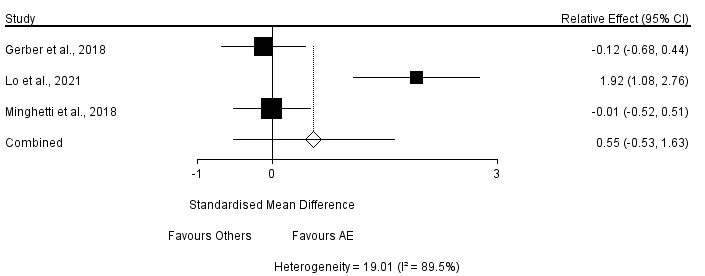


**8.1.2 Network Meta-Analysis for Mental Health Disorders in General**

**[1] Node-Splitting Analysis**

|  | **Direct Effect** | **Indirect Effect** | **Overall** | **P-Value** |
| --- | --- | --- | --- | --- |
| AE, Control | 3.17 (1.71, 4.65) | 3.14 (-1.14, 7.23) | 3.17 (1.77, 4.56) | 0.99 |
| AE, MBE | -1.67 (-8.38, 4.69) | -1.54 (-3.62, 0.60) | -1.54 (-3.51, 0.41) | 0.97 |
| AE, RE | -1.59 (-6.61, 3.41) | -2.19 (-6.46, 2.22) | -1.90 (-5.08, 1.39) | 0.86 |
| AE, Stretching | 4.50 (0.87, 8.16) | 5.65 (-2.81, 14.02) | 4.68 (1.25, 8.03) | 0.81 |
| Control, MBE | -4.71 (-6.24, -3.18) | -4.82 (-11.48, 1.82) | -4.72 (-6.22, -3.21) | 0.98 |
| Control, RE | -5.25 (-9.38, -1.10) | -4.75 (-10.03, 0.56) | -5.07 (-8.25, -1.90) | 0.88 |
| Control, Stretching | 2.62 (-5.96, 11.10) | 1.26 (-2.64, 5.25) | 1.53 (-2.07, 5.03) | 0.78 |

**[2] Convergence Diagnostics**

| **Parameter** | **Potential Scale Reduction Factor** |
| --- | --- |
| d.AE.Control | 1.00 |
| d.AE.MBE | 1.00 |
| d.AE.Others | 1.00 |
| d.AE.RE | 1.00 |
| d.AE.Stretching | 1.00 |
| d.Control.ME | 1.00 |
| sd.d | 1.00 |
| Number of chains: 4  Tuning iterations: 20,000  Simulation iterations: 50,000  Thinning interval: 10  Inference samples: 10,000  Variance scaling factor: 2.5 | |

**[3] Consistency Model**

| AE | 3.17 (1.77, 4.56) | -1.54 (-3.51, 0.41) | -4.14 (-7.59, -0.77) | 1.32 (-4.07, 6.51) | -1.90 (-5.08, 1.39) | 4.68 (1.25, 8.03) |
| --- | --- | --- | --- | --- | --- | --- |
| -3.17 (-4.56, -1.77) | Control | -4.72 (-6.22, -3.21) | -7.33 (-10.47, -4.28) | -1.81 (-7.33, 3.48) | -5.07 (-8.25, -1.90) | 1.53 (-2.07, 5.03) |
| 1.54 (-0.41, 3.51) | 4.72 (3.21, 6.22) | MBE | -2.61 (-6.08, 0.85) | 2.91 (-2.81, 8.39) | -0.35 (-3.82, 3.23) | 6.23 (2.34, 10.11) |
| 4.14 (0.77, 7.59) | 7.33 (4.28, 10.47) | 2.61 (-0.85, 6.08) | ME | 5.52 (-0.80, 11.60) | 2.26 (-2.14, 6.79) | 8.82 (4.21, 13.57) |
| -1.32 (-6.51, 4.07) | 1.81 (-3.48, 7.33) | -2.91 (-8.39, 2.81) | -5.52 (-11.60, 0.80) | Others | -3.25 (-9.30, 3.10) | 3.35 (-2.85, 9.73) |
| 1.90 (-1.39, 5.08) | 5.07 (1.90, 8.25) | 0.35 (-3.23, 3.82) | -2.26 (-6.79, 2.14) | 3.25 (-3.10, 9.30) | RE | 6.58 (1.96, 11.21) |
| -4.68 (-8.03, -1.25) | -1.53 (-5.03, 2.07) | -6.23 (-10.11, -2.34) | -8.82 (-13.57, -4.21) | -3.35 (-9.73, 2.85) | -6.58 (-11.21, -1.96) | Stretching |

**[4] Rank Probability**

| **Rank Probability for Mental Health Disorders Treatment** | | | | | | | |
| --- | --- | --- | --- | --- | --- | --- | --- |
|  | **Rank 1** | **Rank 2** | **Rank 3** | **Rank 4** | **Rank 5** | **Rank 6** | **Rank 7** |
| AE | 0.00 | 0.00 | 0.25 | 0.62 | 0.11 | 0.01 | 0.00 |
| Control | 0.16 | 0.63 | 0.21 | 0.00 | 0.00 | 0.00 | 0.00 |
| MBE | 0.00 | 0.00 | 0.02 | 0.12 | 0.47 | 0.36 | 0.04 |
| ME | 0.00 | 0.00 | 0.00 | 0.01 | 0.04 | 0.15 | 0.80 |
| Others | 0.12 | 0.16 | 0.40 | 0.13 | 0.09 | 0.07 | 0.03 |
| RE | 0.00 | 0.00 | 0.05 | 0.12 | 0.28 | 0.41 | 0.14 |
| Stretching | 0.73 | 0.20 | 0.07 | 0.00 | 0.00 | 0.00 | 0.00 |

(Notes. Rank 1 is worst, rank 7 is best.)

**8.1.3 Meta Regression for Mental Health Disorders in General**

| **Covariate** | **Coefficient** | **Standard**  **Error** | **95% CrI**  **Lower** | **95% CrI**  **Upper** | **Z-value** | **2-Sided**  **P-value** |
| --- | --- | --- | --- | --- | --- | --- |
| **Aerobic Exercise (n = 35)** | | | | | | |
| **Age** | -0.0202 | 0.0135 | -0.0467 | 0.0062 | -1.5 | 0.1333 |
| **Exercise Frequency** | -0.2674 | 0.2266 | -0.7116 | 0.1769 | -1.18 | 0.2382 |
| **Session Duration** | -0.0302 | 0.02 | -0.0694 | 0.009 | -1.51 | 0.1306 |
| **Length of Intervention** | 0.07 | 0.0342 | 0.003 | 0.1369 | 2.05 | 0.0405 |
| **Mind-Body Exercise (n = 35)** | | | | | | |
| **Age** | 0.0144 | 0.0127 | -0.0105 | 0.0393 | 1.13 | 0.2575 |
| **Exercise Frequency** | -0.1076 | 0.0659 | -0.2368 | 0.0216 | -1.63 | 0.1025 |
| **Session Duration** | 0.0051 | 0.0056 | -0.006 | 0.0161 | 0.9 | 0.3666 |
| **Length of Intervention** | 0.0063 | 0.019 | -0.0309 | 0.0435 | 0.33 | 0.7392 |

**8.2 Pairwise Meta-Analysis, Network Meta-Analysis and Meta Regression for Depression**

**8.2.1 Pairwise Meta-Analysis for Depression**

**[1] AE vs. Control (n = 30)**


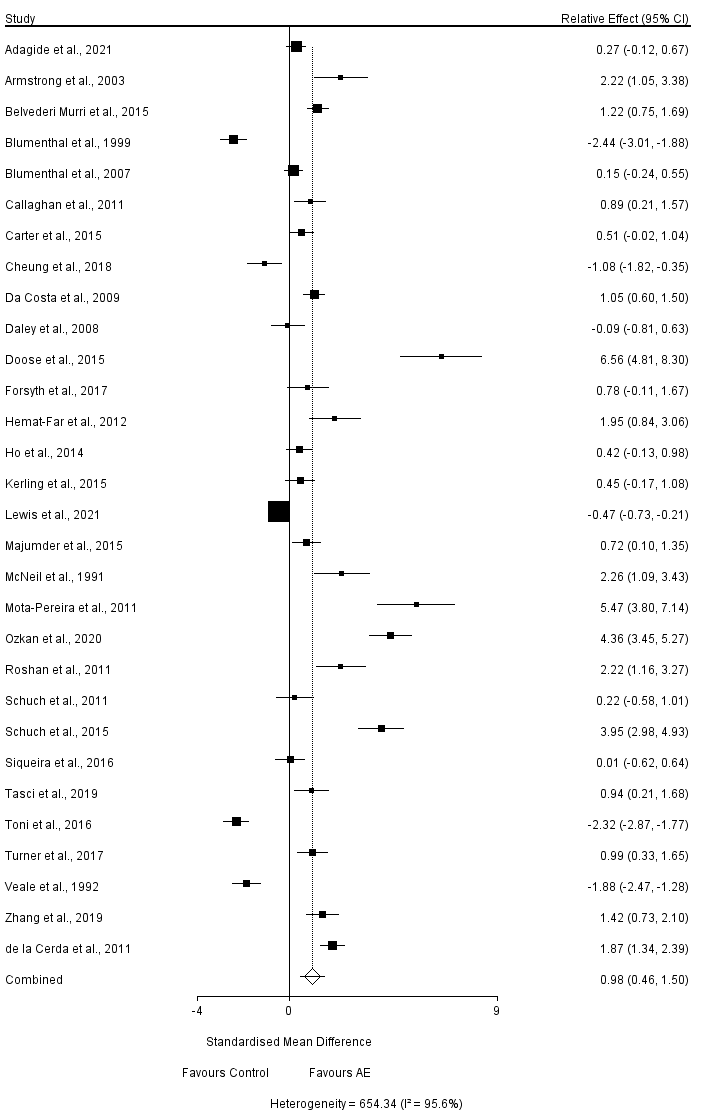


**[2] RE vs. Control (n = 2)**


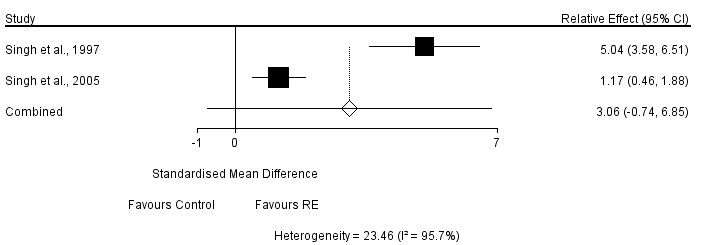


**[3] MBE vs. Control (n = 22)**


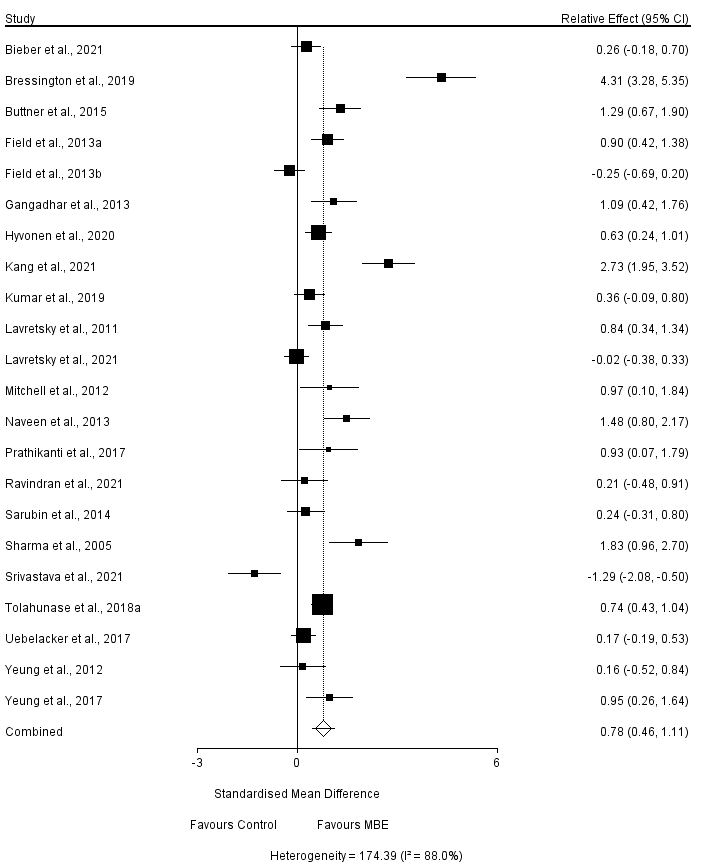


**[4] ME vs. Control (n = 4)**


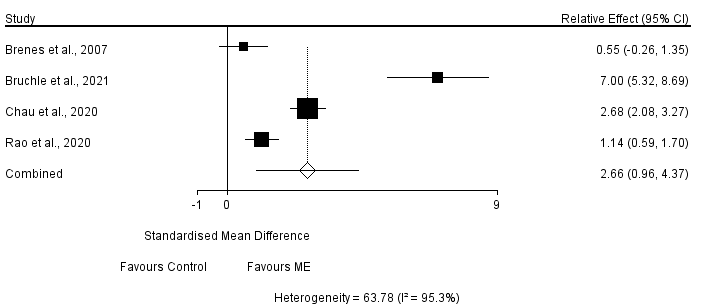


**[5] AE vs. RE (n = 2)**


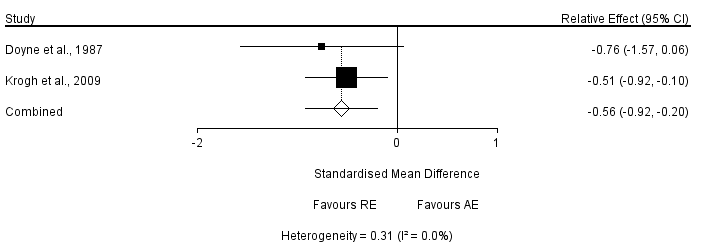


**[6] AE vs. Stretching (n = 6)**


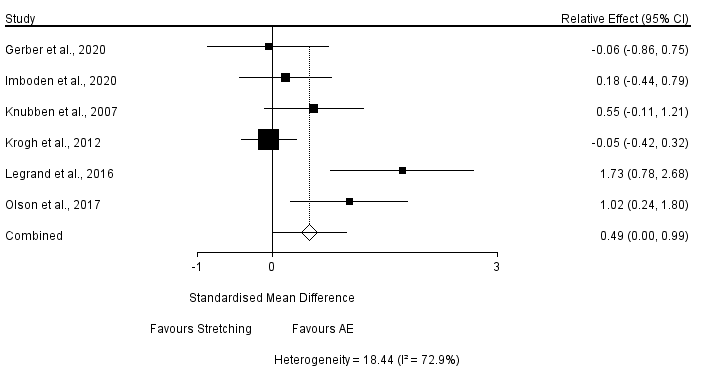


**[7] AE vs. Others (n = 2)**


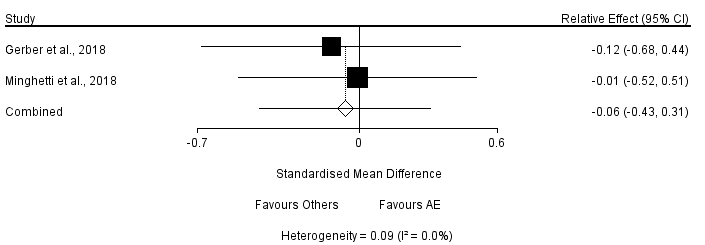


**8.2.2 Network Meta-Analysis for Depression**

**[1] Node-Splitting Analysis**

| **Name** | **Direct Effect** | **Indirect Effect** | **Overall** | **P-Value** |
| --- | --- | --- | --- | --- |
| AE, Control | 3.09 (1.58, 4.65) | 3.65 (-1.33, 8.67) | 3.14 (1.69, 4.59) | 0.83 |
| AE, MBE | 2.60 (-6.30, 11.41) | -0.70 (-3.01, 1.70) | -0.43 (-2.67, 1.80) | 0.48 |
| AE, RE | -1.95 (-7.86, 3.76) | -2.43 (-8.44, 3.64) | -2.26 (-6.35, 1.84) | 0.91 |
| AE, Stretching | 3.57 (-0.17, 7.35) | 5.83 (-2.64, 14.16) | 3.92 (0.53, 7.34) | 0.61 |
| Control, MBE | -3.72 (-5.53, -1.95) | -0.37 (-9.31, 9.05) | -3.58 (-5.34, -1.82) | 0.47 |
| Control, RE | -5.55 (-11.35, 0.09) | -5.22 (-11.08, 0.75) | -5.40 (-9.45, -1.32) | 0.93 |
| Control, Stretching | 2.50 (-5.79, 10.92) | 0.29 (-3.76, 4.47) | 0.78 (-2.80, 4.46) | 0.63 |

**[2] Convergence Diagnostics**

| **Parameter** | **Potential Scale Reduction Factor** |
| --- | --- |
| d.AE.Control | 1.00 |
| d.AE.MBE | 1.00 |
| d.AE.Others | 1.00 |
| d.AE.RE | 1.00 |
| d.AE.Stretching | 1.00 |
| d.Control.ME | 1.00 |
| sd.d | 1.00 |
| Number of chains: 4  Tuning iterations: 20,000  Simulation iterations: 50,000  Thinning interval: 10  Inference samples: 10,000  Variance scaling factor: 2.5 | |

**[3] Consistency Model**

| AE | 3.14 (1.69, 4.59) | -0.43 (-2.67, 1.80) | -4.75 (-9.08, -0.30) | -0.49 (-7.10, 6.09) | -2.26 (-6.35, 1.84) | 3.92 (0.53, 7.34) |
| --- | --- | --- | --- | --- | --- | --- |
| -3.14 (-4.59, -1.69) | Control | -3.58 (-5.34, -1.82) | -7.91 (-12.07, -3.66) | -3.66 (-10.42, 3.00) | -5.40 (-9.45, -1.32) | 0.78 (-2.80, 4.46) |
| 0.43 (-1.80, 2.67) | 3.58 (1.82, 5.34) | MBE | -4.32 (-8.75, 0.23) | -0.05 (-7.06, 6.91) | -1.82 (-6.21, 2.62) | 4.33 (0.47, 8.36) |
| 4.75 (0.30, 9.08) | 7.91 (3.66, 12.07) | 4.32 (-0.23, 8.75) | ME | 4.23 (-3.66, 12.08) | 2.49 (-3.40, 8.27) | 8.66 (3.24, 14.18) |
| 0.49 (-6.09, 7.10) | 3.66 (-3.00, 10.42) | 0.05 (-6.91, 7.06) | -4.23 (-12.08, 3.66) | Others | -1.72 (-9.35, 6.00) | 4.41 (-2.96, 11.96) |
| 2.26 (-1.84, 6.35) | 5.40 (1.32, 9.45) | 1.82 (-2.62, 6.21) | -2.49 (-8.27, 3.40) | 1.72 (-6.00, 9.35) | RE | 6.19 (0.90, 11.33) |
| -3.92 (-7.34, -0.53) | -0.78 (-4.46, 2.80) | -4.33 (-8.36, -0.47) | -8.66 (-14.18, -3.24) | -4.41 (-11.96, 2.96) | -6.19 (-11.33, -0.90) | Stretching |

**[4] Rank Probability**

| **Rank Probability for Depression Treatment** | | | | | | | |
| --- | --- | --- | --- | --- | --- | --- | --- |
|  | **Rank 1** | **Rank 2** | **Rank 3** | **Rank 4** | **Rank 5** | **Rank 6** | **Rank 7** |
| AE | 0.00 | 0.00 | 0.31 | 0.45 | 0.20 | 0.03 | 0.00 |
| Control | 0.29 | 0.60 | 0.11 | 0.00 | 0.00 | 0.00 | 0.00 |
| MBE | 0.00 | 0.01 | 0.20 | 0.34 | 0.34 | 0.11 | 0.01 |
| ME | 0.00 | 0.00 | 0.01 | 0.01 | 0.06 | 0.21 | 0.71 |
| Others | 0.09 | 0.08 | 0.23 | 0.10 | 0.18 | 0.20 | 0.12 |
| RE | 0.00 | 0.01 | 0.07 | 0.09 | 0.22 | 0.45 | 0.16 |
| Stretching | 0.62 | 0.29 | 0.07 | 0.01 | 0.00 | 0.00 | 0.00 |

(Notes. Rank 1 is worst, rank 7 is best.)

**8.2.3 Meta Regression for Depression**

| **Covariate** | **Coefficient** | **Standard**  **Error** | **95% CrI**  **Lower** | **95% CrI**  **Upper** | **Z-value** | **2-sided**  **P-value** |
| --- | --- | --- | --- | --- | --- | --- |
| **Aerobic Exercise (n = 29)** | | | | | | |
| **Age** | -0.0205 | 0.0152 | -0.0503 | 0.0093 | -1.35 | 0.1775 |
| **Exercise Frequency** | -0.3021 | 0.2672 | -0.8259 | 0.2217 | -1.13 | 0.2583 |
| **Session Duration** | -0.0339 | 0.0231 | -0.0791 | 0.0113 | -1.47 | 0.1414 |
| **Length of Intervention** | 0.0709 | 0.0386 | -0.0047 | 0.1465 | 1.84 | 0.066 |
| **Mind-Body Exercise (n = 22)** | | | | | | |
| **Age** | 0.0078 | 0.0166 | -0.0248 | 0.0404 | 0.47 | 0.6387 |
| **Exercise Frequency** | -0.0182 | 0.0911 | -0.1967 | 0.1602 | -0.2 | 0.8411 |
| **Session Duration** | 0.0045 | 0.0069 | -0.009 | 0.0181 | 0.66 | 0.5121 |
| **Length of Intervention** | 0.1423 | 0.0671 | 0.0108 | 0.2738 | 2.12 | 0.034 |

**8.3 Pairwise Meta-Analysis and Network Meta-Analysis for Anxiety Disorder**

**8.3.1 Pairwise Meta-Analysis for Anxiety Disorder**

**[1] AE vs. Control (n = 2)**

**
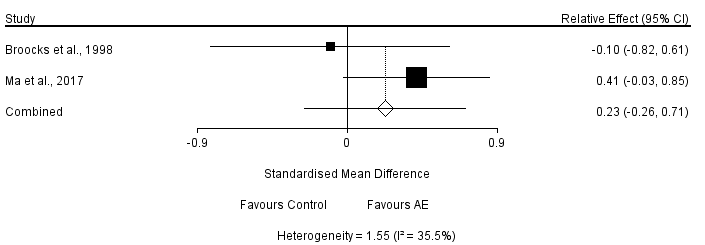
**

**8.3.2 Network Meta-Analysis for Anxiety Disorder**

**[1] Node-Splitting Analysis**

| **Name** | **Direct Effect** | **Indirect Effect** | **Overall** | **P-Value** |
| --- | --- | --- | --- | --- |
| AE, Control | 1.53 (-5.42, 7.66) | 6.72 (-6.13, 19.69) | 2.33 (-3.58, 7.76) | 0.37 |
| AE, RE | -0.29 (-9.74, 9.17) | -5.90 (-17.13, 4.65) | -2.82 (-9.93, 4.22) | 0.37 |
| Control, RE | -7.20 (-16.51, 2.06) | -1.87 (-13.17, 10.23) | -5.17 (-11.88, 2.00) | 0.39 |

**[2] Convergence Diagnostics**

| **Parameter** | **Potential Scale Reduction Factor** |
| --- | --- |
| d.Control.AE | 1.00 |
| d.Control.MBE | 1.00 |
| d.Control.RE | 1.00 |
| sd.d | 1.00 |
| Number of chains: 4  Tuning iterations: 20,000  Simulation iterations: 50,000  Thinning interval: 10  Inference samples: 10,000  Variance scaling factor: 2.5 | |

**[3] Consistency Model**

| AE | 2.33 (-3.58, 7.76) | -0.98 (-11.02, 8.42) | -2.82 (-9.93, 4.22) |
| --- | --- | --- | --- |
| -2.33 (-7.76, 3.58) | Control | -3.37 (-11.46, 4.54) | -5.17 (-11.88, 2.00) |
| 0.98 (-8.42, 11.02) | 3.37 (-4.54, 11.46) | MBE | -1.68 (-12.40, 8.85) |
| 2.82 (-4.22, 9.93) | 5.17 (-2.00, 11.88) | 1.68 (-8.85, 12.40) | RE |

**[4] Rank Probability**

| **Rank Probability for Anxiety Disorder Treatment** | | | | |
| --- | --- | --- | --- | --- |
|  | **Rank 1** | **Rank 2** | **Rank 3** | **Rank 4** |
| AE | 0.13 | 0.44 | 0.35 | 0.09 |
| Control | 0.72 | 0.22 | 0.05 | 0.01 |
| MBE | 0.12 | 0.25 | 0.33 | 0.31 |
| RE | 0.04 | 0.09 | 0.27 | 0.60 |

(Notes. Rank 1 is worst, rank 4 is best.)

**8.4 Pairwise Meta-Analysis and Network Meta-Analysis for Post‐Traumatic Stress Disorder**

**8.4.1 Pairwise Meta-Analysis for Post‐Traumatic Stress Disorder**

**[1] MBE vs. Control (n =7)**


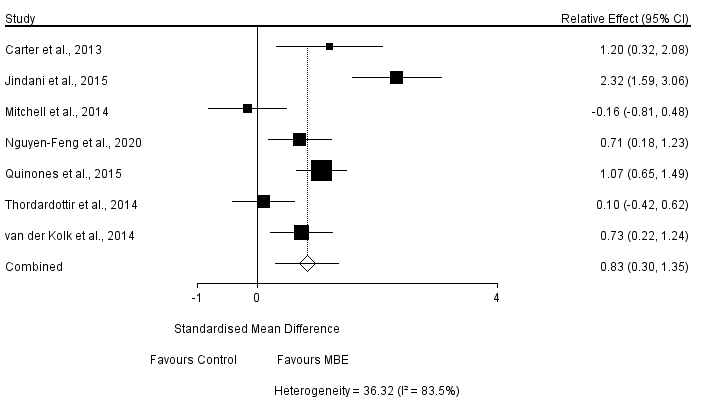


***Sensitivity Analysis*** *(Only including studies Using the Clinician Administered PTSD Scale; n =3)*


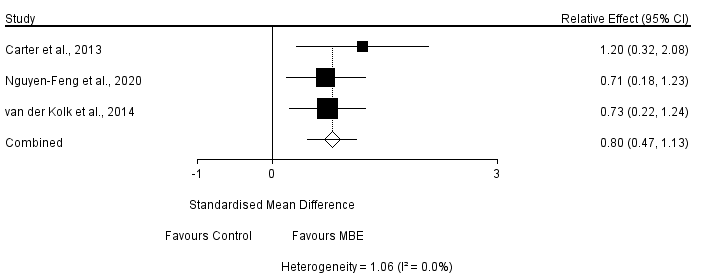


**[2] ME vs. Control (n = 3)**


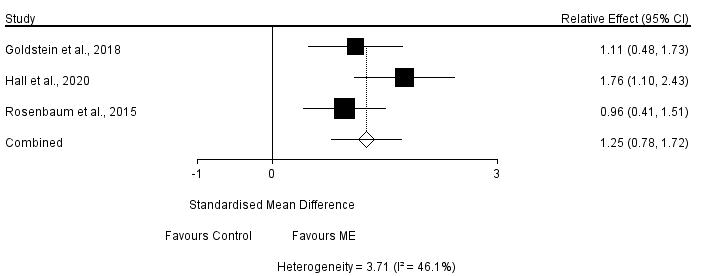


**8.4.2 Network Meta-Analysis for Post‐Traumatic Stress Disorder**

**[1] Convergence Diagnostics**

| **Parameter** | **Potential Scale Reduction Factor** |
| --- | --- |
| d.Control.MBE | 1.00 |
| d.Control.ME | 1.00 |
| d.Control.RE | 1.00 |
| sd.d | 1.00 |
| Number of chains: 4  Tuning iterations: 20,000  Simulation iterations: 50,000  Thinning interval: 10  Inference samples: 10,000  Variance scaling factor: 2.5 | |

**[2] Consistency Model**

| Control | -9.07 (-15.82, -2.60) | -7.84 (-18.02, 1.59) | 0.08 (-18.15, 17.96) |
| --- | --- | --- | --- |
| 9.07 (2.60, 15.82) | MBE | 1.30 (-10.96, 12.69) | 9.16 (-9.95, 28.27) |
| 7.84 (-1.59, 18.02) | -1.30 (-12.69, 10.96) | ME | 7.93 (-12.55, 28.83) |
| -0.08 (-17.96, 18.15) | -9.16 (-28.27, 9.95) | -7.93 (-28.83, 12.55) | RE |

**[3] Rank Probability**

| **Rank Probability for PTSD Treatment** | | | | |
| --- | --- | --- | --- | --- |
|  | **Rank 1** | **Rank 2** | **Rank 3** | **Rank 4** |
| Control | 0.47 | 0.51 | 0.03 | 0.00 |
| MBE | 0.00 | 0.08 | 0.39 | 0.52 |
| ME | 0.03 | 0.15 | 0.47 | 0.36 |
| RE | 0.50 | 0.27 | 0.12 | 0.12 |

(Notes. Rank 1 is worst, rank 4 is best.)

**8.5 Pairwise Meta-Analysis and Network Meta-Analysis for Overall Symptom of Schizophrenia**

**8.5.1 Pairwise Meta-Analysis for Overall Symptom of Schizophrenia**

**[1] AE vs. Control (n = 4)**


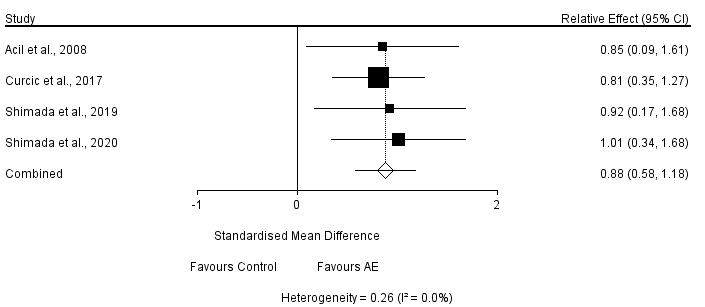


**[2] MBE vs. Control (n = 6)**


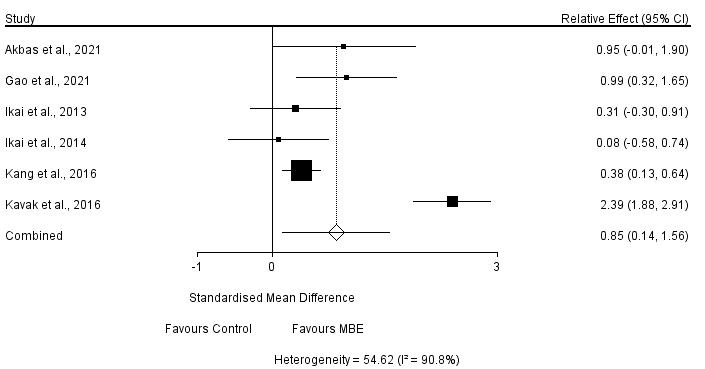


***Sensitivity Analysis*** *(Removing Kavak et al., 2016 measured via Functional Remission of General Schizophrenia Scale)*


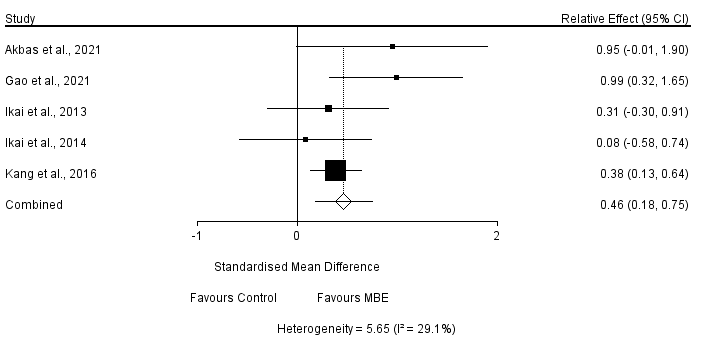


**[3] ME vs. Control (n = 2)**


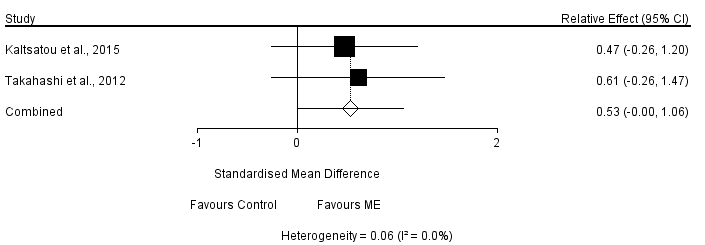


**8.5.2 Network Meta-Analysis for Overall Symptom of Schizophrenia**

**[1] Node-Splitting Analysis**

| **Name** | **Direct Effect** | **Indirect Effect** | **Overall** | **P-Value** |
| --- | --- | --- | --- | --- |
| AE, Control | 6.08 (0.42, 12.61) | -0.44 (-13.10, 12.39) | 4.90 (0.07, 10.35) | 0.31 |
| AE, MBE | -6.16 (-17.90, 5.52) | 0.36 (-6.99, 8.31) | -1.62 (-7.62, 4.93) | 0.31 |
| Control, MBE | -5.68 (-10.45, -0.85) | -12.30 (-25.71, 0.14) | -6.55 (-11.10, -2.08) | 0.30 |

**[2] Convergence Diagnostics**

| **Parameter** | **Potential Scale Reduction Factor** |
| --- | --- |
| d.AE.Control | 1.00 |
| d.AE.MBE | 1.00 |
| d.AE.Others | 1.00 |
| d.AE.Stretching | 1.00 |
| d.Control.ME | 1.00 |
| d.Control.RE | 1.00 |
| sd.d | 1.00 |
| Number of chains: 4  Tuning iterations: 20,000  Simulation iterations: 50,000  Thinning interval: 10  Inference samples: 10,000  Variance scaling factor: 2.5 | |

**[3] Consistency Model**

| AE | 4.90 (0.07, 10.35) | -1.62 (-7.62, 4.93) | -0.85 (-11.40, 10.17) | 4.18 (-6.75, 14.68) | -1.08 (-13.01, 11.12) | 12.32 (-0.93, 25.57) |
| --- | --- | --- | --- | --- | --- | --- |
| -4.90 (-10.35, -0.07) | Control | -6.55 (-11.10, -2.08) | -5.86 (-15.27, 3.55) | -0.75 (-12.87, 10.68) | -6.02 (-16.82, 4.79) | 7.33 (-6.74, 21.17) |
| 1.62 (-4.93, 7.62) | 6.55 (2.08, 11.10) | MBE | 0.67 (-9.65, 10.95) | 5.78 (-6.89, 17.53) | 0.50 (-10.99, 12.13) | 13.96 (-0.63, 28.13) |
| 0.85 (-10.17, 11.40) | 5.86 (-3.55, 15.27) | -0.67 (-10.95, 9.65) | ME | 5.15 (-10.43, 19.61) | -0.08 (-14.32, 13.78) | 13.18 (-3.62, 29.93) |
| -4.18 (-14.68, 6.75) | 0.75 (-10.68, 12.87) | -5.78 (-17.53, 6.89) | -5.15 (-19.61, 10.43) | Others | -5.28 (-21.24, 11.15) | 8.12 (-8.60, 24.64) |
| 1.08 (-11.12, 13.01) | 6.02 (-4.79, 16.82) | -0.50 (-12.13, 10.99) | 0.08 (-13.78, 14.32) | 5.28 (-11.15, 21.24) | RE | 13.30 (-4.49, 30.86) |
| -12.32 (-25.57, 0.93) | -7.33 (-21.17, 6.74) | -13.96 (-28.13, 0.63) | -13.18 (-29.93, 3.62) | -8.12 (-24.64, 8.60) | -13.30 (-30.86, 4.49) | Stretching |

**[4] Rank Probability**

| **Rank Probability for Treatment in Overall Symptom of Schizophrenia** | | | | | | | |
| --- | --- | --- | --- | --- | --- | --- | --- |
|  | **Rank 1** | **Rank 2** | **Rank 3** | **Rank 4** | **Rank 5** | **Rank 6** | **Rank 7** |
| AE | 0.00 | 0.01 | 0.07 | 0.31 | 0.33 | 0.20 | 0.08 |
| Control | 0.08 | 0.42 | 0.40 | 0.09 | 0.01 | 0.00 | 0.00 |
| MBE | 0.00 | 0.01 | 0.04 | 0.12 | 0.25 | 0.35 | 0.24 |
| ME | 0.02 | 0.06 | 0.11 | 0.17 | 0.17 | 0.19 | 0.28 |
| Others | 0.11 | 0.33 | 0.21 | 0.13 | 0.08 | 0.07 | 0.07 |
| RE | 0.02 | 0.07 | 0.11 | 0.15 | 0.15 | 0.18 | 0.31 |
| Stretching | 0.77 | 0.11 | 0.06 | 0.03 | 0.02 | 0.01 | 0.01 |

(Notes. Rank 1 is worst, rank 7 is best.)

**8.6 Pairwise Meta-Analysis and Network Meta-Analysis for Positive Symptom of Schizophrenia**

**8.6.1 Pairwise Meta-Analysis for Positive Symptom of Schizophrenia**

**[1] AE vs. Control (n = 6)**


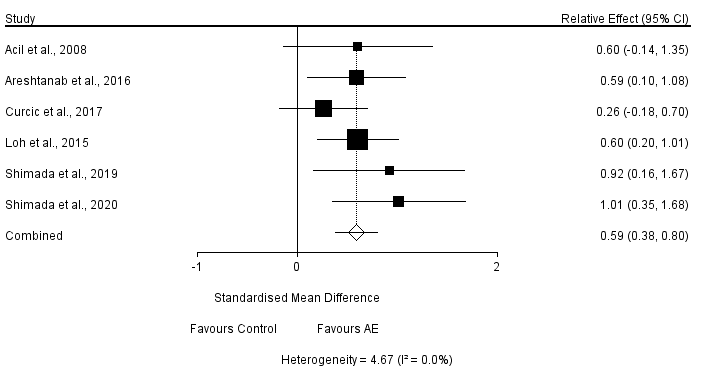


**[2] RE vs. Control (n = 2)**


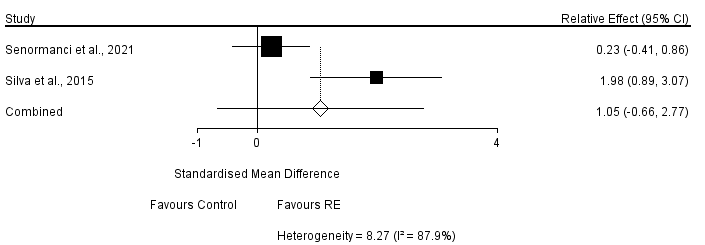


**[3] MBE vs. Control (n = 9)**


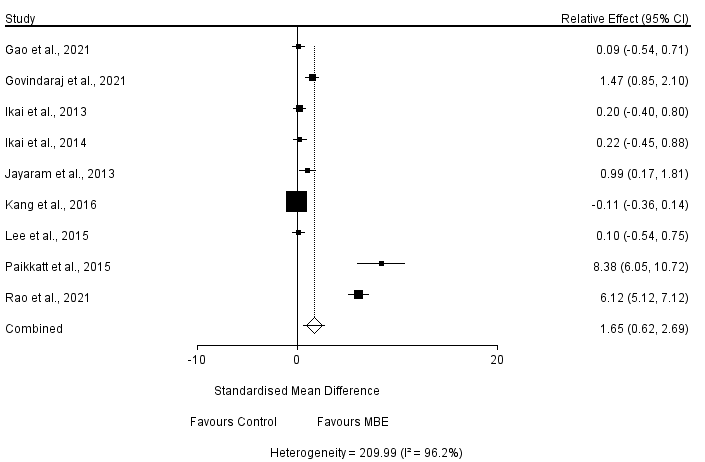


(Notes. As the heterogeneity existed broadly in assessment and exercise characteristics, the further subgroup analysis and sensitivity were not applicable after testing.)

**[4] ME vs. Control (n = 2)**


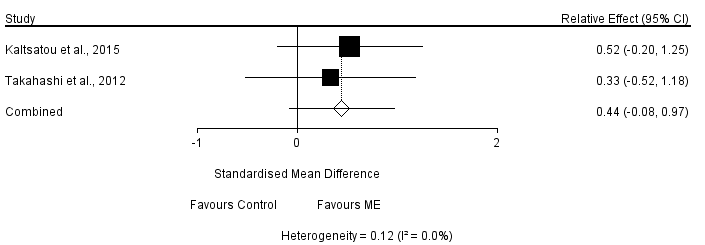


**[5] AE vs. MBE (n = 3)**


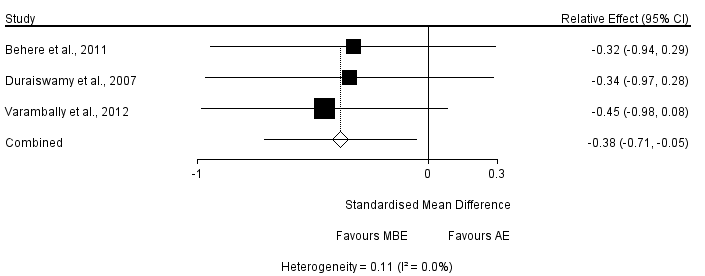


**[6] AE vs. Stretching (n = 2)**


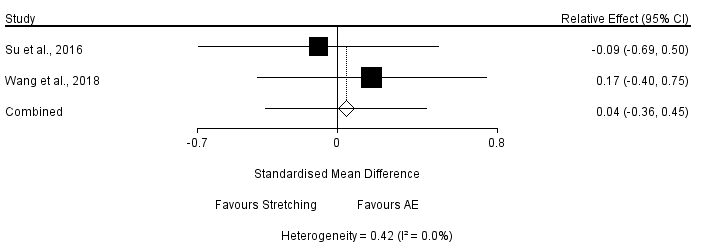


**8.6.2 Network Meta-Analysis for Positive Symptom of Schizophrenia (Adjusted)**

**[1]Node-Splitting Analysis**

| **Name** | **Direct Effect** | **Indirect Effect** | **Overall** | **P-Value** |
| --- | --- | --- | --- | --- |
| AE, Control | 2.41 (0.29, 4.70) | 0.59 (-2.99, 4.24) | 1.93 (0.10, 3.88) | 0.36 |
| AE, MBE | -1.93 (-5.14, 1.31) | -0.05 (-2.78, 2.74) | -0.82 (-2.93, 1.30) | 0.36 |
| Control, MBE | -2.47 (-4.32, -0.83) | -4.33 (-8.34, -0.55) | -2.75 (-4.41, -1.25) | 0.36 |

**[1] Convergence Diagnostics**

| **Parameter** | **Potential Scale Reduction Factor** |
| --- | --- |
| d.AE.Control | 1.00 |
| d.AE.MBE | 1.00 |
| d.AE.Others | 1.00 |
| d.AE.Stretching | 1.00 |
| d.Control.RE | 1.00 |
| sd.d | 1.00 |
| Number of chains: 4  Tuning iterations: 20,000  Simulation iterations: 50,000  Thinning interval: 10  Inference samples: 10,000  Variance scaling factor: 2.5 | |

**[2] Consistency Model**

| AE | 1.93 (0.10, 3.88) | -0.82 (-2.93, 1.30) | 0.77 (-4.09, 5.57) | -0.41 (-4.98, 4.28) | 0.17 (-3.55, 3.88) |
| --- | --- | --- | --- | --- | --- |
| -1.93 (-3.88, -0.10) | Control | -2.75 (-4.41, -1.25) | -1.15 (-6.42, 3.87) | -2.33 (-6.71, 1.89) | -1.74 (-6.00, 2.33) |
| 0.82 (-1.30, 2.93) | 2.75 (1.25, 4.41) | MBE | 1.61 (-3.64, 6.82) | 0.42 (-4.10, 4.97) | 1.00 (-3.36, 5.22) |
| -0.77 (-5.57, 4.09) | 1.15 (-3.87, 6.42) | -1.61 (-6.82, 3.64) | Others | -1.18 (-7.58, 5.44) | -0.55 (-6.63, 5.58) |
| 0.41 (-4.28, 4.98) | 2.33 (-1.89, 6.71) | -0.42 (-4.97, 4.10) | 1.18 (-5.44, 7.58) | RE | 0.57 (-5.26, 6.43) |
| -0.17 (-3.88, 3.55) | 1.74 (-2.33, 6.00) | -1.00 (-5.22, 3.36) | 0.55 (-5.58, 6.63) | -0.57 (-6.43, 5.26) | Stretching |

**[3] Rank Probability**

| **Rank Probability for Treatment in Positive Symptom of Schizophrenia** | | | | | | |
| --- | --- | --- | --- | --- | --- | --- |
|  | **Rank 1** | **Rank 2** | **Rank 3** | **Rank 4** | **Rank 5** | **Rank 6** |
| AE | 0.00 | 0.09 | 0.30 | 0.38 | 0.19 | 0.04 |
| Control | 0.51 | 0.36 | 0.11 | 0.02 | 0.00 | 0.00 |
| MBE | 0.00 | 0.03 | 0.10 | 0.21 | 0.36 | 0.30 |
| Others | 0.26 | 0.20 | 0.15 | 0.11 | 0.12 | 0.16 |
| RE | 0.09 | 0.14 | 0.15 | 0.13 | 0.18 | 0.31 |
| Stretching | 0.14 | 0.18 | 0.19 | 0.15 | 0.16 | 0.19 |

(Notes. Rank 1 is worst, rank 6 is best.)

**8.7 Pairwise Meta-Analysis, Network Meta-Analysis and Meta-Regression for Negative Symptom of Schizophrenia**

**8.7.1 Pairwise Meta-Analysis for Negative Symptom of Schizophrenia**

**[1] AE vs. Control (n = 5)**


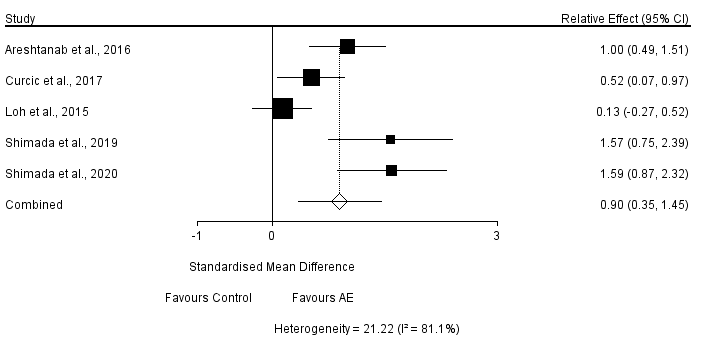


**Sensitivity Analysis (After removing Loh et al., 2015)**


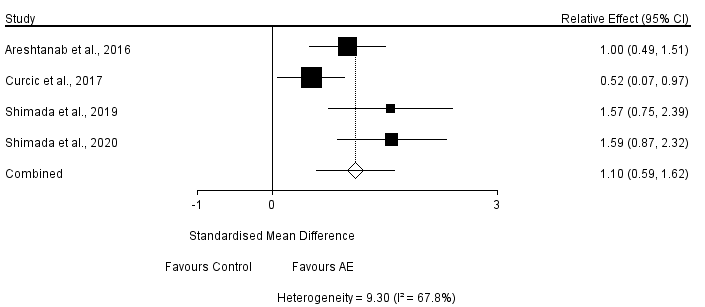


**[2] RE vs. Control (n = 2)**


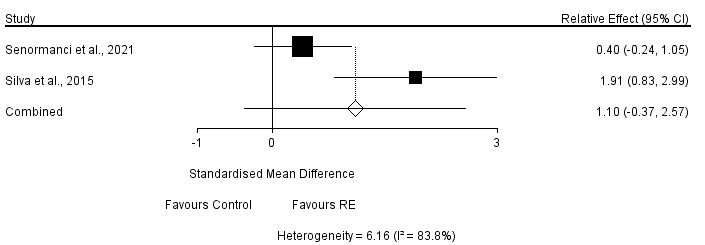


**[3] MBE vs. Control (n = 12)**


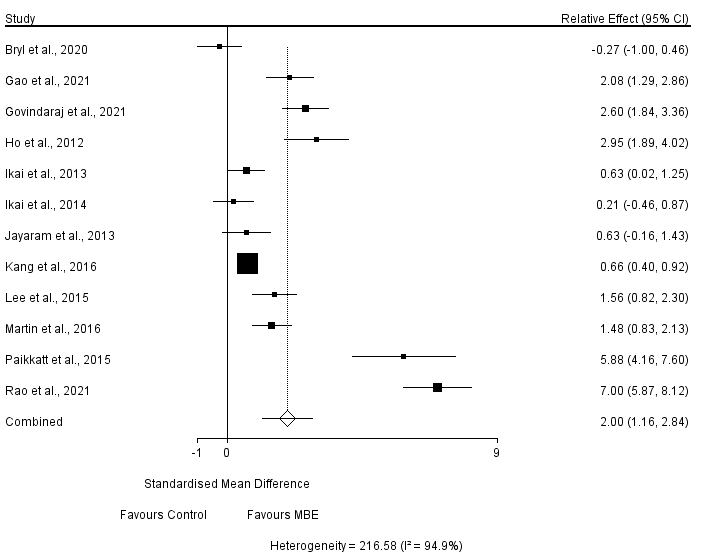


**[4] ME vs. Control (n = 2)**


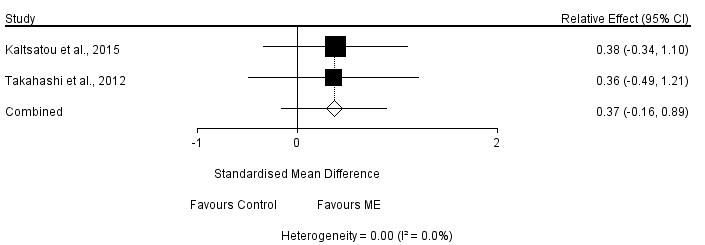


**[5] AE vs. MBE (n = 3)**


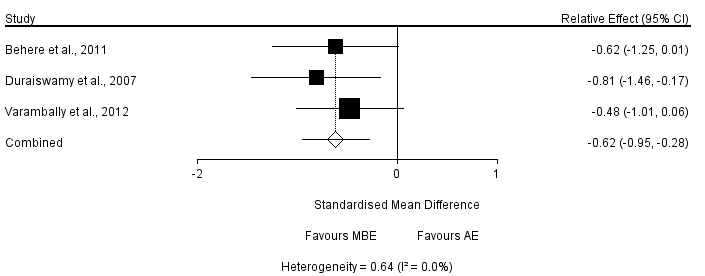


**[6] AE vs. Stretching (n = 2)**


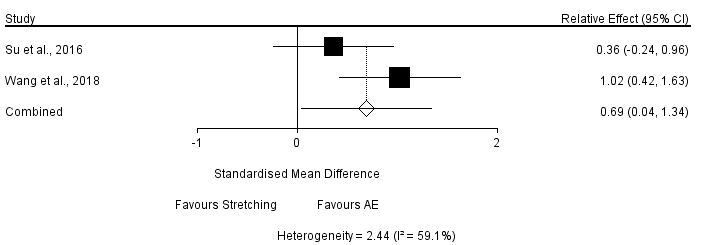


**8.7.2 Network Meta-Analysis for Negative Symptom of Schizophrenia**

**[1] Node-Splitting Analysis**

|  | **Direct Effect** | **Indirect Effect** | **Overall** | **P-Value** |
| --- | --- | --- | --- | --- |
| AE, Control | 3.20 (-1.95, 8.35) | 3.32 (-4.11, 10.80) | 3.28 (-0.81, 7.46) | 0.99 |
| AE, MBE | -2.34 (-8.84, 4.28) | -2.32 (-8.53, 3.78) | -2.26 (-6.65, 2.15) | 1.00 |
| Control, MBE | -6.32 (-9.66, -3.06) | -2.40 (-9.58, 4.59) | -5.55 (-8.51, -2.65) | 0.29 |
| Control, ME | -1.67 (-9.44, 5.95) | -14.54 (-25.23, -3.83) | -6.07 (-12.88, 0.47) | 0.06 |
| MBE, ME | -8.38 (-18.60, 1.98) | 4.44 (-3.47, 12.42) | -0.52 (-7.45, 6.33) | 0.05 |

**[2] Convergence Diagnostics**

| **Parameter** | **Potential Scale Reduction Factor** |
| --- | --- |
| d.AE.Control | 1.00 |
| d.AE.MBE | 1.00 |
| d.AE.Others | 1.00 |
| d.AE.Stretching | 1.00 |
| d.Control.ME | 1.00 |
| d.Control.RE | 1.00 |
| sd.d | 1.00 |
| Number of chains: 4  Tuning iterations: 20,000  Simulation iterations: 50,000  Thinning interval: 10  Inference samples: 10,000  Variance scaling factor: 2.5 | |

**[3] Consistency Model**

| AE | 3.28 (-0.81, 7.46) | -2.26 (-6.65, 2.15) | -2.86 (-10.44, 5.06) | 0.05 (-10.70, 10.95) | -0.33 (-9.98, 9.12) | 3.76 (-4.49, 11.90) |
| --- | --- | --- | --- | --- | --- | --- |
| -3.28 (-7.46, 0.81) | Control | -5.55 (-8.51, -2.65) | -6.07 (-12.88, 0.47) | -3.27 (-15.12, 8.46) | -3.63 (-12.24, 4.96) | 0.46 (-8.72, 9.68) |
| 2.26 (-2.15, 6.65) | 5.55 (2.65, 8.51) | MBE | -0.52 (-7.45, 6.33) | 2.36 (-9.46, 14.29) | 1.95 (-7.09, 10.95) | 6.03 (-3.60, 15.30) |
| 2.86 (-5.06, 10.44) | 6.07 (-0.47, 12.88) | 0.52 (-6.33, 7.45) | ME | 2.86 (-10.44, 16.26) | 2.52 (-8.53, 13.12) | 6.66 (-4.98, 17.95) |
| -0.05 (-10.95, 10.70) | 3.27 (-8.46, 15.12) | -2.36 (-14.29, 9.46) | -2.86 (-16.26, 10.44) | Others | -0.37 (-15.28, 14.12) | 3.70 (-10.29, 17.23) |
| 0.33 (-9.12, 9.98) | 3.63 (-4.96, 12.24) | -1.95 (-10.95, 7.09) | -2.52 (-13.12, 8.53) | 0.37 (-14.12, 15.28) | RE | 4.08 (-8.45, 16.76) |
| -3.76 (-11.90, 4.49) | -0.46 (-9.68, 8.72) | -6.03 (-15.30, 3.60) | -6.66 (-17.95, 4.98) | -3.70 (-17.23, 10.29) | -4.08 (-16.76, 8.45) | Stretching |

**[4] Rank Probability**

| **Rank Probability for Treatment in Negative Symptom of Schizophrenia** | | | | | | | |
| --- | --- | --- | --- | --- | --- | --- | --- |
|  | **Rank 1** | **Rank 2** | **Rank 3** | **Rank 4** | **Rank 5** | **Rank 6** | **Rank 7** |
| AE | 0.00 | 0.05 | 0.24 | 0.36 | 0.24 | 0.08 | 0.02 |
| Control | 0.27 | 0.43 | 0.23 | 0.06 | 0.01 | 0.00 | 0.00 |
| MBE | 0.00 | 0.01 | 0.04 | 0.12 | 0.28 | 0.38 | 0.18 |
| ME | 0.01 | 0.04 | 0.08 | 0.11 | 0.16 | 0.23 | 0.37 |
| Others | 0.18 | 0.13 | 0.12 | 0.12 | 0.11 | 0.12 | 0.21 |
| RE | 0.11 | 0.13 | 0.15 | 0.14 | 0.15 | 0.15 | 0.18 |
| Stretching | 0.43 | 0.22 | 0.14 | 0.08 | 0.05 | 0.04 | 0.03 |

(Notes. Rank 1 is worst, rank 7 is best.)

**8.7.3 Meta Regression for Negative Symptom of Schizophrenia**

| **Covariate** | **Coefficient** | **Standard**  **Error** | **95%**  **Lower** | **95%**  **Upper** | **Z-value** | **2-sided**  **P-value** |
| --- | --- | --- | --- | --- | --- | --- |
| **Mind-Body Exercise (n = 12)** | | | | | | |
| **Age** | -0.1086 | 0.1246 | -0.3528 | 0.1356 | -0.87 | 0.3834 |
| **Exercise Frequency** | 0.959 | 0.4684 | 0.041 | 1.877 | -2.05 | 0.0406 |
| **Session Duration** | -0.0097 | 0.0416 | -0.0912 | 0.0718 | -0.23 | 0.8153 |
| **Length of Intervention** | -0.0202 | 0.0781 | -0.1734 | 0.1329 | -0.26 | 0.7959 |

| **Abbreviations** | |
| --- | --- |
| AE | aerobic exercise |
| RE | resistance exercise |
| MBE | mind-body exercise |
| ME | Multimodal exercise |
